# Synthesis of Novel *N*-Acylhydrazones and Their C-N/N-N Bond Conformational Characterization by NMR Spectroscopy

**DOI:** 10.3390/molecules26164908

**Published:** 2021-08-13

**Authors:** Rubina Munir, Noman Javid, Muhammad Zia-ur-Rehman, Muhammad Zaheer, Rahila Huma, Ayesha Roohi, Muhammad Makshoof Athar

**Affiliations:** 1 Institute of Chemistry, University of the Punjab, Lahore 54590, Pakistan; atharmakshoof@gmail.com; 2 Department of Chemistry, Kinnaird College for Women, Lahore 54000, Pakistan; rahila.huma@kinnaird.edu.pk (R.H.); ayesha.roohi@kinnaird.edu.pk (A.R.); 3 Department of Chemistry (C-Block), Forman Christian College, Ferozepur Road, Lahore 54600, Pakistan; noumanjavid@gmail.com; 4 Applied Chemistry Research Centre, PCSIR Laboratories Complex, Lahore 54600, Pakistan; mzaheerchem@yahoo.com

**Keywords:** quinoline, pyrazolo[3,4-*b*]quinoline, conformer, rotamer, NMR, stereoisomer, acylhydrazone

## Abstract

In this article, a synthesis of *N*’-(benzylidene)-2-(6-methyl-1*H*-pyrazolo[3,4-*b*]quinolin-1-yl)acetohydrazides and their structural interpretation by NMR experiments is described in an attempt to explain the duplication of some peaks in their ^1^H- and ^13^C-NMR spectra. Twenty new 6-methyl-1*H*-pyrazolo[3,4-*b*]quinoline substituted *N*-acylhydrazones **6**(**a**–**t**) were synthesized from 2-chloro-6-methylquinoline-3-carbaldehyde (**1**) in four steps. 2-Chloro-6-methylquinoline-3-carbaldehyde (1) afforded 6-methyl-1*H*-pyrazolo[3,4-*b*]quinoline (**2**), which upon *N*-alkylation yielded 2-(6-methyl-1*H*-pyrazolo[3,4-*b*]quinolin-1-yl)acetate (**3**). The hydrazinolysis of 3 followed by the condensation of resulting 2-(6-methyl-1*H*-pyrazolo[3,4-*b*]quinolin-1-yl)acetohydrazide (**4**) with aromatic aldehydes gave *N*-acylhydrazones **6**(**a**–**t**). Structures of the synthesized compounds were established by readily available techniques such as FT-IR, NMR and mass spectral studies. The stereochemical behavior of **6**(**a**–**t**) was studied in dimethyl sulfoxide-*d_6_* solvent by means of ^1^H NMR and ^13^C NMR techniques at room temperature. NMR spectra revealed the presence of *N*’-(benzylidene)-2-(6-methyl-1*H*-pyrazolo[3,4-*b*]quinolin-1-yl)acetohydrazides as a mixture of two conformers, i.e., *E*_(C=N)(N-N)_ *synperiplanar* and *E*_(C=N)(N-N)_ *antiperiplanar* at room temperature in DMSO-*d_6_*. The ratio of both conformers was also calculated and *E*_(C=N) (N-N)_ *syn-periplanar* conformer was established to be in higher percentage in equilibrium with the *E*_(C=N) (N-N)_ *anti-periplanar* form.

## 1. Introduction

*N*-Acylhydrazone (NAH) scaffold has been identified as a lead pharmacophore in many bioactive compounds that act against different molecular targets [1,2,3,4,5,6,7,8]. This moiety is a putative bioisostere of amide backbone [9]. Its achiral nature and higher chemical stability than peptide bond have urged the medicinal chemists to consider this privileged scaffold for structural design of new drug candidates [10,11,12,13,14,15,16]. Due to the presence of H-bonding sites and viability of adopting multiple conformational orientations, *N*-acylhydrazones have a promising prospective to interact with various bioreceptors. Thus, NAH subunit is of central importance in the process of molecular recognition by the target as well as during structure–activity relationship studies (SARs) [17,18,19].

The *N*-acyl hydrazone backbone (-C(O)-N-N=C<) is an assemblage of amide and imine functional groups which is likely to exhibit geometric as well as conformational stereoisomerism [20]. Rotation along imine C=N linkage may give rise to two geometrical stereoisomers, i.e., *E* and *Z* forms. By means of ^1^H- and ^13^C NMR data, Palla and coworkers speculated that *N*-Acyl hydrazones of aromatic aldehydes tend to exist in *E* configuration in solid form [21]. In solution too, the less hindered *E* is the preferred geometry; however, the *Z* isomer can be detected in fewer polar solvents [22,23]. The configurational interconversion in solution is quite slow due to restriction of rotation about C=N bond and the achievement of equilibrium in solution requires hours (or even days) [24]. The *E*/*Z* switching usually requires energy and can be achieved thermodynamically [20,24], photochemically [25,26,27], or chemically by base or acid catalysis [28,29,30,31].

**Figure 1:**
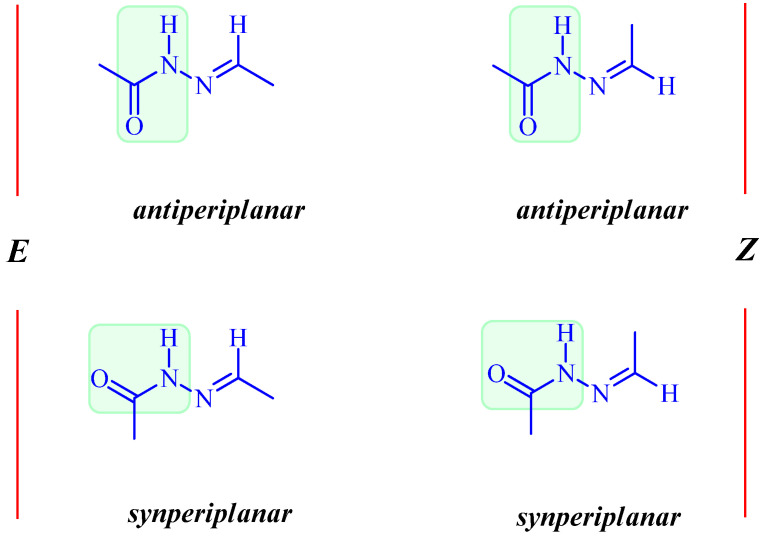
Stereoisomers of *N*-Acylhydrazones.

Other than geometrical isomerism, rotation across amide C(O)-NH bond may give rise to conformational stereoisomerism [20,32]. Referring to the arrangement of NH bond *cis* or *trans* to carbonyl bond, NAH may exist as *synperiplanar* (*sp*) and *antiperiplanar* (*ap*) conformers (Figure 1). NMR spectroscopy is a useful tool to distinguish the stereoisomers in *N*-acylhydrazones [33,34]. Though there is a general tendency of NH to undergo rapid exchange with the solvent, fortunately the C(O)-NH proton in *N*-acylhydrazones exhibits conspicuous signals for each isomer and provides a better assessment because neither this proton undergoes considerable exchange nor the signal gets superimposed by aromatic protons [35].

Until now, the researchers have focused on stereoisomers arising from C=N and C(O)-NH bond rotation; however, rotation about the N-N bond remained overlooked. In this study, we demonstrate the synthesis and investigation of the stereochemical behavior of new *N*-acylhydrazones of 2-(6-Methyl-1*H*-pyrazolo[3,4-*b*]quinolin-1-yl)acetohydrazide by interpretation of the signal duplication in their ^1^H NMR and ^13^C NMR spectra in DMSO-*d_6_* solvent. Since the comprehensive information including stereochemistry of a molecule is very important for optimization in drug design and discovery; the conformers arising from rotation around N-N bond too have been considered to depict a clear picture of the steric factors.

## 2. Results and Discussion

### 2.1. Synthetic Chemistry

**Scheme 1:**
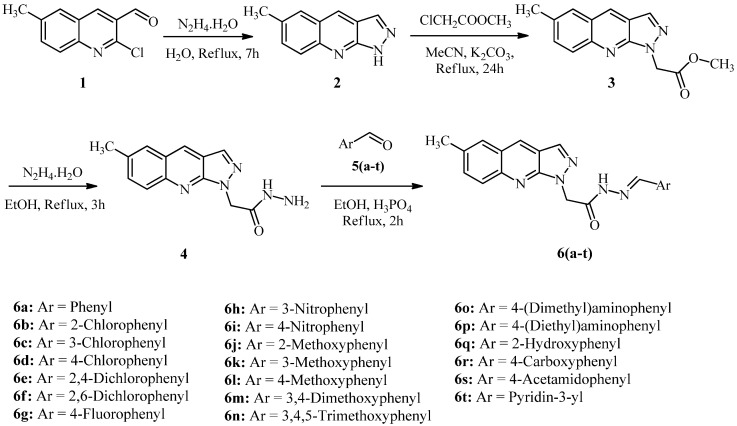
Synthesis of 6-methyl-1*H*-pyrazolo[3,4-*b*]quinoline substituted *N*-acylhydrazones **6**(**a**–**t**).

New *N*’-(benzylidene)-2-(6-methyl-1*H*-pyrazolo[3,4-*b*]quinolin-1-yl)acetohydrazides **6**(**a**–**t**) were synthesized in four steps as illustrated in Scheme 1.

2-Chloro-6-methylquinoline-3-carbaldehyde (**1**) was used as precursor for the synergism of quinoline ring with pyrazole scaffold to afford 6-methyl-1*H*-pyrazolo[3,4-*b*]quinoline (**2**) after cyclocondensation with hydrazine monohydrate. 6-Methyl-1*H*-pyrazolo[3,4-*b*]quinoline (**2**) was converted to methyl 2-(6-methyl-1*H*-pyrazolo[3,4-*b*]quinolin-1-yl)acetate (**3**) by reacting it with methyl chloroacetate under S_N_2 conditions. Hydrazinolysis of the synthesized ester (**3**) resulted in the formation of 2-(6-methyl-1*H*-pyrazolo[3,4-*b*]quinolin-1-yl)acetohydrazide (**4**), which was finally condensed with a variety of aromatic aldehydes **5**(**a**–**t**) in acidic conditions to afford titled *N*-acylhydrazones **6**(**a**–**t**) in 82–99% yield. The progress of reaction was monitored by TLC and structures of the synthesized compounds **6**(**a**–**t**) were elucidated using different techniques including FT-IR, ^1^H NMR, ^13^C NMR spectroscopies and mass spectrometry (see Supplementary Materials). The purity of the titled compounds was established from elemental analysis.

### 2.2. Spectroscopic Characterization

Primarily, the functional groups of the synthesized products were determined by recording their vibrational spectra. The IR spectra of methyl 2-(6-methyl-1*H*-pyrazolo[3,4-*b*]quinolin-1-yl)acetate (**3**) displayed a strong absorption band at 1741 cm^−1^ for C=O bond while C-O stretching vibration at 1217 cm^−1^ which confirmed the presence of ester functionality. The ^1^H NMR spectrum of the compound **3** showed a singlet peak of methylene protons at 5.44 ppm. The ester linkage was confirmed from a sharp singlet at 3.78 ppm referring to -OCH_3_ protons. Aromatic protons exhibited signals between 7.62 and 8.56 ppm among which H-4 of the pyrazolo[3,4-*b*]quinoline ring emerged as the most deshielded proton. In the ^13^C NMR spectrum of the compound **3**, signals for carbonyl carbon and -OCH_3_ group of ester were observed at 169.3 and 52.8 ppm, respectively, while methylene carbon showed up at 48.3 ppm.

The FT-IR spectrum of hydrazide **4** disclosed the absorption band of C=O at 1665 cm^−1^ which indicated the transformation of ester linkage into hydrazide. The two individual absorption bands referring to NH stretching vibrations at 3345 cm^−1^ and 3256 cm^−1^ proved the hydrazide formation. In ^1^H NMR spectrum of the compound **4**, the absence of methoxy protons at 3.78 ppm and presence of two singlet peaks of NH protons confirmed the structure of hydrazide. The broad singlet of C(O)-NH proton emerged downfield at 9.39 ppm due to significant deshielding, whereas -NH_2_ protons resonated upfield at 4.32 ppm. The signal of methylene protons appeared downfield resonating near 5.14 ppm. Aromatic ring protons exhibited peaks between 7.65 ppm and 9.39 ppm. H-3 and H-4 exhibited distinct singlets at 8.44 ppm and 8.84 ppm, respectively; however, the chemical shifts and multiplicity of the rest aromatic signals varied according to their position on the ring. Ar-CH_3_ protons showed up as singlet at 2.51 ppm though the signal was overlapped by the solvent signal. The ^13^C NMR spectrum displayed C=O carbon at 166.9 ppm.

Structures of the titled *N*-acylhydrazones **6**(**a**–**t**) were explicated by infrared spectroscopy, NMR spectroscopy and elemental analysis. The presence of C=O and C=N stretching vibrations at 1643–1658 cm^−1^ and 1596–1627 cm^−1^, but NH absorption band around 3195–3310 cm^−1^, confirmed the acyl hydrazone backbone in all the compounds. The synthesized compounds were also analyzed by mass spectrometry. The molecular masses of few representative compounds determined from mass spectra were in agreement with the given structures, hence validating the formation of the titled *N*-acylhydrazones.

**Figure 2:**
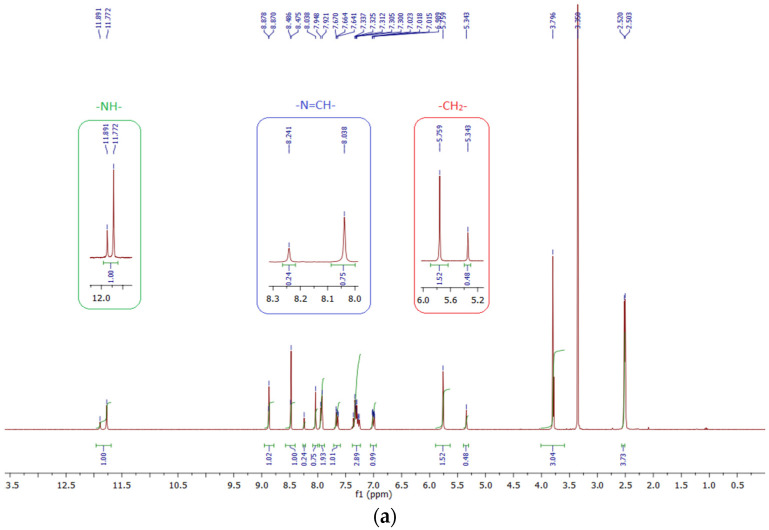

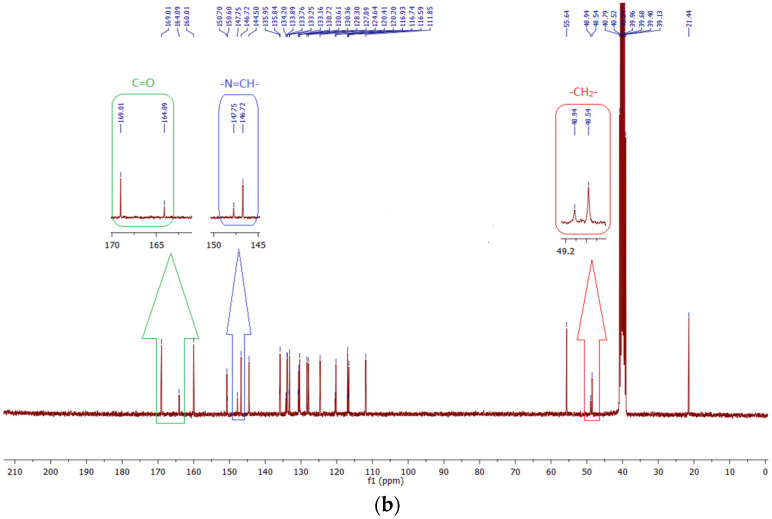
(**a**) ^1^H NMR and (**b**) ^13^C NMR spectra of *N*-Acylhydrazone **6k**.

In the ^1^H NMR spectra (recorded in DMSO-*d_6_*) of the synthesized *N*-acylhydrazones **6**(**a**–**t**), the characteristic peak for -CH_3_ appeared at 2.52 ppm, which was unfortunately disguised by the solvent signal. NH proton resonated between 11.40–12.20 ppm whereas the methylene and aromatic protons appeared in their respective region. H-4 of pyrazoloquinoline ring was eminent as the most deshielded aromatic proton which displayed a singlet resonating around 8.86–8.88 ppm. The signal of H-3 too, emerged as a singlet near 8.48 ppm. The peak for imine N=CH proton was observed around 7.95–8.50 ppm. The ^13^C NMR spectra displayed -CH_3_ carbon at 21.5 ppm and methylene carbon near 48 ppm. Carbonyl carbon and azomethine N=CH carbon exhibited peaks near 169 ppm and 146–149 ppm, respectively. In ^1^H NMR of the synthesized *N*-acylhydrazones **6**(**a**–**t**), duplicate appearance of signals was observed for NH, N=CH, -CH_2_- and aryl protons. The same behavior was witnessed in ^13^C NMR spectra (Figure 2).

### 2.3. Stereochemical Characterization of N-Acylhydrazones

In all the ^1^H NMR and ^13^C NMR spectra, a particular pattern of two explicit sets of signals for certain protons was observed. Although similar in pattern, the heights of the duplicated peaks varied from compound to compound indicating the existence of two stereoisomers in solution in dissimilar ratio (Figure 2). Structures of the two isomers in DMSO-*d_6_* are resolved by considering all the plausible configurations and conformations arising due to C=N, C(O)-NH and N-N bond rotations in order to determine whether the duplication of some peaks in their ^1^H- and ^13^C-NMR spectra was associated with a mixture of geometric (*E*/*Z*) stereoisomers or rotational stereoisomers (*synperiplanar* and *antiperiplanar*).

**Figure 3:**
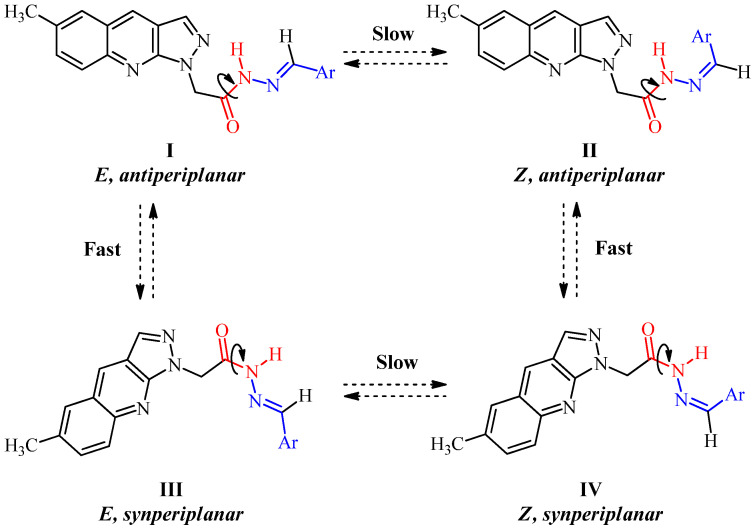
Rotational isomers arising due to C-N Bond C(O)-NH rotation.

The first possibility is the existence of compounds **6**(**a**–**t**) as two geometrical isomers, i.e., *Z* and *E* forms in solution which arise due to rotation along imine C=N bond [20]. On the other hand, the synthesized compounds are likely to exist as rotational stereoisomers (*synperiplanar* and *antiperiplanar*) due restriction of rotation about C-N bond of amide linkage C(O)-NH [20,21] (Figure 3).

**Figure 4:**
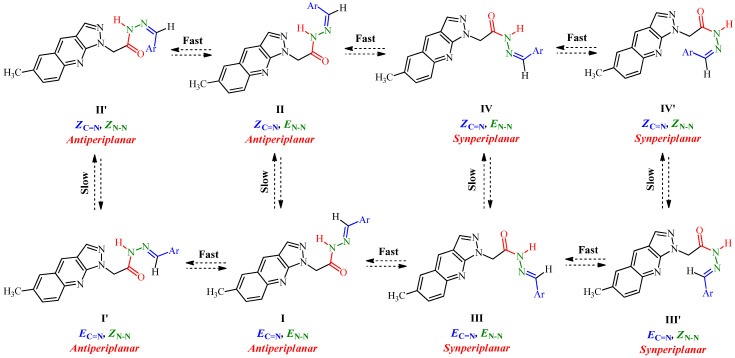
All the possible stereoisomers of *N*-Acylhydrazones **6**(**a**–**t**).

In this study, the rotational isomers **Iʹ**–**IVʹ** arising from rotation around N-N bond, are demonstrated for all the four stereoisomers **I**–**IV** to illustrate a clear picture of the steric factors (Figure 4).

Literature studies have established the *Z*_(C=N)_ configuration to be the short-lived isomeric form due to steric crowding leading towards relatively less stability than *E*_(C=N)_ forms [20,36,37]. As the illustration of representative forms **II**, **IIʹ** and **IVʹ** clearly depicts the crowding of groups (Figure 4); hence, this is not the preferred geometric configuration. The relative amounts of *E*_(C=N)_/*Z*_(C=N)_ isomers also depend upon the solvent; the *Z*_(C=N)_ form, due to intramolecular Hydrogen bonding, can be detected in less polar solvents such as chloroform. The 100% isomer in DMSO-*d_6_*, however, is the *E* isomer [22,23]. Furthermore, the chemical shifts for NH signals of *Z*_(C=N)_ isomers of *N*-acylhydrazones are reported around 14 ppm [37]; no signal in this region was observed for the synthesized compounds **6**(**a**–**t**). The geometry of the titled compounds was further confirmed from the thin layer chromatography and LC-MS techniques. *E*_(C=N)_ and *Z*_(C=N)_ isomers have significantly different R_f_ values to difference in polarity and dipole moment of the molecules [38,39,40,41]. During the characterization of the synthesized compounds **6**(**a**–**t**), single spot in TLC negated the existence of geometric isomers in the solution under study. Based on literature studies and behavior of compounds during chromatography, the presence of *Z*_(C=N)_ forms (**II**, **IIʹ**, **IV** and **IVʹ**) in the solutions of **6**(**a**–**t**) was ruled out.

The emergence of the duo signals in the ^1^H-NMR spectra of the compounds is, hence, ascribed to the existence of rotational isomers. The conformers arising due to N-N bond rotation have also been considered; however, *Z*_(N–N)_ (**Iʹ**–**IVʹ**) conformations (dihedral angle = 0°) are unlikely to exist due to steric crowding of acyl group and benzylidene part of the molecule. The co-planarity and eclipsing of C=O and C=N bonds in forms **Iʹ** and **IIʹ** while steric hindrance in forms **IIIʹ** and **IVʹ** demonstrates that *Z*_(N–N)_ forms are not likely to prevail (Figure 4). Relative prevalence of *E*_(C=N)_ geometric isomer and *E*_(N-N)_ conformer suggests that the duplicate signals in the ^1^H NMR spectra of the synthesized compounds refer to the existence of conformers about amide linkage only, hence the two isomers observed in NMR spectra are anticipated to be *E*_(C=N) (N-N)_-*antiperiplanar* and *E*_(C=N) (N-N)_-*synperiplanar* forms **I** and **III** (Figure 3 and Figure 4). Spectral studies regarding rotamers, i.e., *synperiplanar* and *antiperiplanar* conformers of *N*-acyl hydrazones have already been described by researchers [42,43,44].

The paired peaks, emerging as two discrete sets of singlets in the spectra of the synthesized compounds, are designated to amide C(O)NH, imine N=CH and methylene -CH_2_- protons. For C(O)NH and N=CH protons, the downfield signals are assigned to the *antiperiplanar* (*ap*) conformer while the upfield signals to the *synperiplanar* (*sp*) conformers, as established from literature [42,43,44]. On the contrary, for methylene protons the upfield signal is denoted as *anti*- while downfield signal as the *syn*- isomer, as is evident from the integration of the signals relative to those of imine and amide peaks (Table 1).

**Figure 5:**
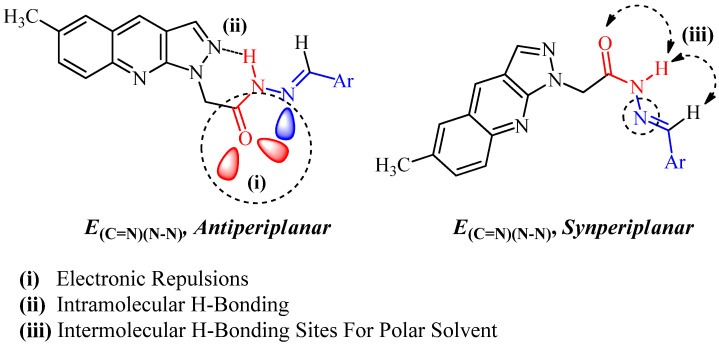
Possible interactions of *E*-Isomer in solution.

The number and ratio of stereoisomers in solution is a function of solvent and temperature [24]. Moreover, *N*-acylhydrazones of aromatic aldehydes are reported to exist in dimeric forms, arising as a result of intermolecular H-bonding [45]. For this type of H-bonding, the most equitable arrangement of the molecule is *syn*-periplanar conformation with *E*_(C=N) (N-N)_ configuration (Form **III**), as this form possesses C=O and N-N groups *trans* with respect to each other which not only allow the development of electrostatic interactions to form dimers but are also available for interaction with polar solvents (Figure 5). The dimerization of *N*-acylhydrazones may take place in DMSO according to the literature studies [24]; however, in this research work, no experimental evidence of this was observed. Due to the electronic repulsions in *anti*- form and availability of sites for intermolecular hydrogen bonding interactions between solute and solvent molecules, the ratio of *syn*- conformer is expected to be higher in DMSO, as this conformation is the most appropriate arrangement for this type of interaction.

The C(O)-NH signals of the *syn*- form appeared at around 11.41–2.04 ppm, and for the *anti*- form at 11.66–12.18 ppm. The signals of methylene protons of the *syn*- form resonated around 5.67–5.80 ppm, while those of the *anti*- form were in the range 5.29–5.39 ppm (Table 1). The C-N bond rotation has also influenced the chemical shifts of aromatic protons which have exhibited paired signals, though the multiplicity of most of the aromatic signals has not been particularly allocated due to superimposition of signals.

The percentage of both the conformers were calculated by taking the ratio of integral intensities of the paired peaks (Table 2). The higher ratio is allotted to *synperiplanar* isomer. The calculations from the ^1^H NMR spectra of compounds **6(a**–**t)** revealed that the DMSO-*d_6_* solution of these compounds contained *E*_(C=N)(N-N)_-*synperiplanar* and *E*_(C=N)(N-N)_-*antiperiplanar* conformers in an approximate ratio 3:1 (75% *syn*- isomer while 25% *anti*- conformer) with slight variation from compound to compound.

**Figure 6:**
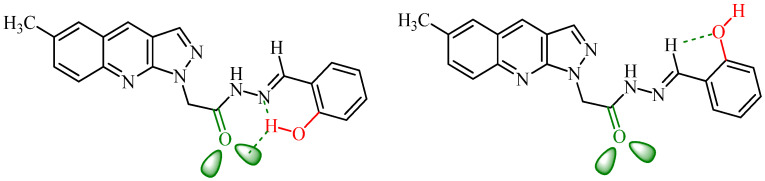
Possible intramolecular interactions in *E*_(C=N) (N-N)—_*Antiperiplanar* form of **6q**.

An exceptionally higher percentage ratio of *antiperiplanar* form is demonstrated in the spectrum of compound **6q**. In **6q**, the two isomers have shown approximately equal amounts that are attributed to the presence of -OH substituent at *ortho* position of phenyl ring, leading towards the increased stability of *anti*- conformation via intramolecular H-bonding (Figure 6).

^13^C NMR spectra of the synthesized compounds **6**(**a**–**t**) too, displayed duplicated signals which indicated the presence of a mixture of conformational stereoisomers [46]. The duplicated signals of three representative carbon (C=O, N=CH and -CH_2_-) atoms are identified and precisely interpreted in order to recognize and validate the isomers. For carbonyl carbon, two signals were observed; the upfield peak was referred to *ap* conformer while the downfield signal was assigned to *sp* conformer. Conversely, the downfield signal for azomethine stood for *ap* rotamer while upfield peak for to *sp* conformer. The methylene carbon also gave two signals, among which the signal for *sp* isomer appeared upfield (Table 3).

## 3. Materials and Methods

### 3.1. General

All the chemicals (solvents and reagents) were purchased from renowned chemical manufacturing companies (Alpha Aesar: Ward Hill, MA, USA, Fluka: Buchs, Switzerland, Merck: Darmstadt, Germany and Sigma/Aldrich: Saint Louis, MO, USA) through their indigenous suppliers. Most of the chemicals were used without further purification. Thin layer chromatography was executed on aluminum plates coated with silica gel 60 F254 (Merck) in a suitable mobile phase and spots were visualized under ultraviolet irradiation (λ = 366 and 254 nm). Melting points were noted on Gallenkamp melting point apparatus by the open capillary method and are uncorrected. FTIR spectra were recorded on an Agilent Technologies Cary 630 FTIR spectrophotometer (Santa Clara, CA, USA). ^1^H NMR spectra were recorded in CDCl_3_ and DMSO-*d_6_* on Brücker Avance NMR (300 MHz, Billerica, MA, USA) instrument using TMS as internal standard. Chemical shifts were recorded in δ (ppm) and coupling constant (*J*) is stated in Hertz. ^13^C NMR spectra were recorded on an operating radio frequency of 75 MHz. NMR spectra were recorded at room temperature. Mass spectra were recorded on an LCQ Advantage Max Thermofisher instrument (Poway, CA, USA) using ESI+ mode. Elemental analysis was achieved on a LECO 630-200-200 TRUSPEC CHNS micro analyzer (St. Joseph, MI, USA) and the values observed are within ±0.4% of the calculated results. Compounds **1** and **2** were synthesized by using literature procedures [47,48].

### 3.2. Synthesis of Methyl 2-(6-Methyl-1H-pyrazolo[3,4-b]quinolin-1-yl)acetate (**3**)

To a stirred solution of 6-methyl-1*H*-pyrazolo[3,4-*b*]quinoline (**2**) (1.83 g; 10 mmol) in acetonitrile (25 mL) was added anhydrous potassium bicarbonate (2.07 g; 15 mmol). Subsequently, methylchloroacetate (1.3 mL; 1.6 g; 15 mmol) was added to the resulting mixture followed by heating under reflux conditions for 24 h. Excess solvent was removed under vacuum and the contents were filtered after the addition of water. The crude product was dried and purified by column chromatography (Eluting solvent: *n*-Hexane-ethyl acetate (7:3)). 

Yield 71%. Light brown solid. m.p. 260–261 °C. FT-IR (ῡ cm^–1^; neat): 3021 (CH-Aromatic), 2927 (CH), 1741 (C=O), 1217 (C-O). ^1^H NMR (CDCl_3_, 300 MHz) *δ* = 2.57 (s, 3H, -CH_3_), 3.78 (s, 3H, -OCH_3_), 5.44 (s, 2H, -CH_2_-), 7.62 (dd, *J* = 8.7, 2.1 Hz, 1H, H-7), 7.75 (s, 1H, H-5), 8.01 (d, *J* = 8.7 Hz, 1H, H-8), 8.30 (s, 1H, H-3), 8.56 (s, 1H, H-4) ppm. ^13^C NMR (DMSO-*d_6_*, 75 MHz) *δ* = 21.4 (-CH_3_), 48.3 (-CH_2_-), 52.8 (-OCH_3_), 116.8 (C-13), 124.8 (C-11), 127.9 (C-5), 128.3 (C-8), 130.9 (C-7), 133.4 (C-3), 134.0 (C-4), 134.5 (C-6), 146.8 (C-10), 150.3 (C-12), 169.3 (C=O) ppm. MS *m*/*z*: 256.28 [M^+^ + 1]. Anal. Calcd. for C_14_H_13_N_3_O_2_: C, 65.87; H, 5.13; N, 16.46%. Found: C, 65.95; H, 5.31; N, 16.68%. 

### 3.3. Synthesis of 2-(6-Methyl-1H-pyrazolo[3,4-b]quinolin-1-yl)acetohydrazide (**4**) 

Hydrazine monohydrate (98%; 1.6 mL; 50 mmol) was added to the stirred solution of methyl 2-(6-methyl-1*H*-pyrazolo[3,4-*b*]quinolin-1-yl)acetate (**3**) (6.375 g; 25 mmol) in absolute ethanol (50 mL) and the reaction mixture was refluxed for 3 h. Excess solvent was removed on rotavapor and water was added to the obtained residue. Neutralization of the resulting solution with 2N HCl solution yielded precipitates which were filtered, washed with water and cold ethanol respectively, and dried. 

Yield 80%. Off-white solid. m.p. 246–248 °C. FT-IR (ῡ cm^–1^; neat): 3345 (NH_2_), 3256 (NH), 3040 (CH-Aromatic), 2928 (CH), 1665 (C=O). ^1^H NMR (DMSO-*d_6_*, 300 MHz) *δ* = 2.51 (s, 3H, -CH_3_), 4.32 (s, 2H, -NH_2_), 5.14 (s, 2H, -CH_2_-), 7.65 (d, *J* = 9.0 Hz, 1H, H-7), 7.91–7.94 (m, 2H, H-5, H-8), 8.44 (s, 1H, H-3), 8.84 (s, 1H, H-4), 9.39 (s, 1H, -NH-) ppm. ^13^C NMR (DMSO-*d_6_*, 75 MHz) *δ* = 21.4 (-CH_3_), 48.3 (-CH_2_-), 116.9 (C-13), 124.6 (C-11), 127.9 (C-5), 128.3 (C-8), 130.6 (C-7), 133.1 (C-3), 133.8 (C-4), 134.0 (C-6), 146.7 (C-10), 150.5 (C-12), 166.9 (C=O) ppm. MS *m/z*: 256.39 [M^+^ + 1]. Anal. Calcd. for C_13_H_13_N_5_O: C, 61.17; H, 5.13; N, 27.43%. Found: C, 61.49; H, 5.39; N, 27.75%. 

### 3.4. General Procedure for the Synthesis of N-Acyl hydrazones **6**(**a**–**t**)

Title compounds **6**(**a**–**t**) were synthesized by refluxing an ethanolic solution of 2-(6-Methyl-1*H*-pyrazolo[3,4-*b*]quinolin-1-yl)acetohydrazide (**4**) (1.275 g; 5 mmol) and corresponding aromatic aldehyde **5**(**a**–**u**) (5 mmol) with catalytic amount of orthophosphoric acid. The products precipitated within 0.5–2.0 h were filtered, washed with cold ethanol, and dried in the oven. Off-white or pale-yellow solids were obtained after recrystallization from absolute ethanol. 

*(E)-N’-benzylidene-2-(6-methyl-1H-pyrazolo[3,4-b]quinolin-1-yl)acetohydrazide* (**6a**) Yield 85%, pale yellow solid. m.p. 266–268 °C. FT-IR (ῡ cm^–1^; neat): 3290 (NH), 3042 (CH-Aromatic), 2932 (CH), 1658 (C=O), 1604 (C=N). ^1^H NMR (DMSO-*d_6_*, 300 MHz): *δ* = 2.52_(sp + ap)_ (s, 3H, -CH_3_), 5.35_ap_, 5.75_sp_ (2 s, 2H, -CH_2_-), 7.41–7.45_(sp__+__ap)_ (m, 3H, H-3′, H-4′, H-5′), 7.64–7.73_(sp + ap)_ (m, 3H, H-7, H-2′, H-6′), 7.9–7.96_(sp__+__ap)_ (m, 2H, H-5, H-8), 8.06_sp_, 8.28_ap_ (2 s, 1H, N = CH), 8.48_sp_, 8.49_ap_ (s, 1H, H-3), 8.87_(sp + ap)_ (s, 1H, H-4), 11.75_sp_, 11.88_ap_ (2 s, 1H, NH) ppm. ^13^C NMR (DMSO-*d_6_*, 75 MHz) *δ* = 21.5 (-CH_3_), 48.5_sp_, 48.9_ap_ (-CH_2_-), 116.9, 124.7, 127.5, 127.6, 127.9, 128.3, 129.3, 130.5, 130.6, 133.2, 133.8, 133.9, 134.4, 134.5, 144.6, 146.7_sp_, 147.9_ap_ (N=CH), 150.7, 164.1_ap_, 169.0_sp_ (C=O) ppm. Anal. Calcd. for C_20_H_17_N_5_O: C, 69.96; H, 4.99; N, 20.40%. Found: C, 70.14; H, 5.11; N, 20.62%. 

*(E)-N’-(2-chlorobenzylidene)-2-(6-methyl-1H-pyrazolo[3,4-b]quinolin-1-yl)acetohydrazide* (**6b**) Yield 84%, pale yellow solid, m.p. 256–258 °C. FT-IR (ῡ cm^–1^; neat): 3302 (NH), 3038 (CH-Aromatic), 2929 (CH), 1651 (C=O), 1615 (C=N), 771 (C-Cl). ^1^H NMR (DMSO-*d_6_*, 300 MHz) *δ* = 2.52_(sp + ap)_ (s, 3H, -CH_3_), 5.35_ap_, 5.77_sp_ (2 s, 2H, -CH_2_-), 7.33_(sp + ap)_ (t, *J* = 7.5 Hz, 1H, H-5′), 7.44_(sp + ap)_ (td, *J* = 7.8 Hz, 1.8 Hz, 1H, H-4′), 7.54_(sp + ap)_ (d, *J* = 7.8 Hz, 1H, H-6′), 7.66_(sp + ap)_ (dd, *J* = 9.0 Hz, 1.8 Hz, 1H, H-7), 7.93–7.96_(sp + ap)_ (m, 2H, H-5, H-8), 8.02_(sp + ap)_ (dd, *J* = 8.1 Hz, 1.5 Hz, 1H, H-3′), 8.45_sp_, 8.66_ap_ (2 s, 1H, N=CH), 8.48_sp_, 8.50_ap_ (s, 1H, H-3), 8.87_sp_, 8.89_ap_ (s, 1H, H-4), 11.92_sp_, 12.12_ap_ (2 s, 1H, NH) ppm. ^13^C NMR (DMSO-*d_6_*, 75 MHz) *δ* = 21.4 (-CH_3_), 48.6_sp_, 49.0_ap_ (-CH_2_-), 116.9, 124.7, 127.5, 127.9, 128.0, 128.1, 128.3, 130.3, 130.6, 131.6, 131.7, 131.9, 133.2, 133.5, 133.8, 134.0, 134.3, 140.6, 143.8, 146.7_sp_, 147.9_ap_ (N=CH), 150.7, 164.3_ap_, 169.1_sp_ (C=O) ppm. MS *m*/*z*: 378.91 [M^+^ + 1]. Anal. Calcd. for C_20_H_16_ClN_5_O: C, 63.58; H, 4.27; N, 18.54%. Found: C, 63.30; H, 4.09; N, 18.40%. 

*(E)-N’-(3-chlorobenzylidene)-2-(6-methyl-1H-pyrazolo[3,4-b]quinolin-1-yl)acetohydrazide* (**6c**) Yield 94%. Off-white solid. m.p. 257–259 °C. FT-IR (ῡ cm^–1^; neat): 3307 (NH), 3040 (CH-Aromatic), 2928 (CH), 1656 (C=O), 1610 (C=N), 774 (C-Cl). ^1^H NMR (DMSO-*d_6_*, 300 MHz) *δ* = 2.52_(sp + ap)_ (s, 3H, -CH_3_), 5.36_ap_, 5.77_sp_ (2 s, 2H, -CH_2_-), 7.41–7.50_(sp + ap)_ (m, 2H, H-5′, H-6′), 7.63–7.69_(sp + ap)_ (m, 2H, H-7, H-4′), 7.76_ap_, 7.83_sp_ (2 t, *J* = 1.5 Hz, 1H, H-2′), 7.92–7.95_(sp + ap)_ (m, 2H, H-5, H-8), 8.04_sp_, 8.26_ap_ (2 s, 1H, N=CH), 8.47_sp_, 8.49_ap_ (s, 1H, H-3), 8.87_sp_, 8.88_ap_ (s, 1H, H-4), 11.86_sp_, 12.02_ap_ (2 s, 1H, NH) ppm. ^13^C NMR (DMSO-*d_6_*, 75 MHz) *δ* = 21.4 (-CH_3_), 48.6_sp_, 48.9_ap_ (-CH_2_-), 116.9, 124.7, 126.2, 126.3, 126.6, 127.0, 128.3, 130.1, 130.6, 130.7, 131.1, 131.2, 133.2, 133.8, 133.9, 134.2, 136.6, 136.8, 143.1, 146.2, 146.7_sp_ (N=CH), 150.7, 164.3_ap_, 169.2_sp_ (C=O) ppm. Anal. Calcd. for C_20_H_16_ClN_5_O: C, 63.58; H, 4.27; N, 18.54%. Found: C, 63.65; H, 4.41; N, 18.68%.

*(E)-N’-(4-chlorobenzylidene)-2-(6-methyl-1H-pyrazolo[3,4-b]quinolin-1-yl)acetohydrazide* (**6d**) yield 92%. Off-white solid. m.p. 262–264 °C. FT-IR (ῡ cm^–1^; neat): 3296 (NH), 3021 (CH-Aromatic), 2928 (CH), 1643 (C=O), 1596 (C=N), 774 (C-Cl). ^1^H NMR (DMSO-*d_6_*, 300 MHz) *δ* = 2.52_(sp + ap)_ (s, 3H, -CH_3_), 5.35_ap_, 5.75_sp_ (2 s, 2H, -CH_2_-), 7.47_sp_, 7.51_ap_ (2 d, *J* = 8.7 Hz, 2H, H-2′, H-6′), 7.66_(sp + ap)_ (dd, *J* = 9.0 Hz, 1.5 Hz, 1H, H-7), 7.72–7.77_(sp + ap)_ (m, 2H, H-3′, H-5′), 7.92–7.95_(sp + ap)_ (m, 2H, H-5, H-8), 8.05_sp_, 8.26_ap_ (2 s, 1H, N=CH), 8.47_sp_, 8.49_ap_ (s, 1H, H-3), 8.87_sp_, 8.88_ap_ (s, 1H, H-4), 11.81_sp_, 11.95_ap_ (2 s, 1H, NH) ppm. ^13^C NMR (DMSO-*d_6_*, 75 MHz) *δ* = 21.4 (-CH_3_), 48.5_sp_, 48.8_ap_ (-CH_2_-), 116.7, 124.4, 124.7, 125.4, 127.9, 128.2, 128.3, 130.7, 133.8, 133.9, 134.1, 135.0, 144.7, 146.7_sp_, 147.5_ap_ (N=CH), 148.1, 150.3, 163.7_ap_, 169.3_sp_ (C=O) ppm. Anal. Calcd. for C_20_H_16_ClN_5_O: C, 63.58; H, 4.27; N, 18.54%. Found: C, 63.74; H, 4.59; N, 18.80%.

*(E)-N’-(2,4-dichlorobenzylidene)-2-(6-methyl-1H-pyrazolo[3,4-b]quinolin-1-yl)acetohydrazide* (**6e**) Yield 85%. Off-white solid. Decomposed at 272 °C. FT-IR (ῡ cm^–1^; neat): 3265 (NH), 3025 (CH-Aromatic), 2931 (CH), 1651 (C=O), 1601 (C=N), 775 (C-Cl). ^1^H NMR (DMSO-*d_6_*, 300 MHz) *δ* = 2.52_(sp + ap)_ (s, 3H, -CH_3_), 5.35_ap_, 5.77_sp_ (2 s, 2H, -CH_2_-), 7.39_sp_, 7.50_ap_ (dd, *J* = 8.7 Hz, 2.1 Hz, 1H, H-5′), 7.66_(sp + ap)_ (dd, *J* = 9.0 Hz, 1.8 Hz, 1H, H-7), 7.73_(sp + ap)_ (d, *J* = 2.1 Hz, 1H, H-3′), 7.92–7.95_(sp + ap)_ (m, 2H, H-5, H-8), 8.03_(sp + ap)_ (d, *J* = 8.4 Hz, 1H, H-6′), 8.38_sp_, 8.60_ap_ (2 s, 1H, N=CH), 8.48_sp_, 8.49_ap_ (s, 1H, H-3), 8.87_sp_, 8.88_ap_ (s, 1H, H-4), 11.96_sp_, 12.16_ap_ (2 s, 1H, NH) ppm. ^13^C NMR (DMSO-*d_6_*, 75 MHz) *δ* = 21.4 (-CH_3_), 48.5_sp_ (-CH_2_-), 116.9, 124.7, 127.9, 128.3, 128.5, 128.7, 129.8, 130.6, 130.8, 133.2, 133.3, 133.8, 134.0, 134.2, 134.3, 135.4, 139.6, 142.8, 146.7_sp_ (N=CH), 150.7, 164.3_ap_, 169.2_sp_ (C=O) ppm. Anal. Calcd. for C_20_H_15_C_l__2_N_5_O: C, 58.27; H, 3.67; N, 16.99%. Found: C, 58.15; H, 3.51; N, 16.75%.

*(E)-N’-(2,6-dichlorobenzylidene)-2-(6-methyl-1H-pyrazolo[3,4-b]quinolin-1-yl)acetohydrazide* (**6f**) Yield 82%. Pale yellow solid. m.p. 268–270 °C. FT-IR (ῡ cm^–1^; neat): 3233 (NH), 3030 (CH-Aromatic), 2925 (CH), 1650 (C=O), 1619 (C=N), 771 (C-Cl). _1_H NMR (DMSO-*d_6_*, 300 MHz) *δ* = 2.52_(sp + ap)_ (s, 3H, -CH_3_), 5.38_ap_, 5.67_sp_ (2 s, 2H, -CH_2_-), 7.41–7.48_(sp + ap)_ (m, 1H, H-4′), 7.55–7.68_(sp + ap)_ (m, 3H, H-7, H-3′, H-5′), 7.91–7.97_(sp + ap)_ (m, 2H, H-5, H-8), 8.35_sp_, 8.46_ap_ (s, 1H, N=CH), 8.47_sp_, 8.50_ap_ (s, 1H, H-3), 8.87_sp_, 8.88_ap_ (s, 1H, H-4), 12.04_sp_, 12.18_ap_ (2 s, 1H, NH) ppm. ^13^C NMR (DMSO-*d_6_*, 75 MHz) *δ* = 21.4 (-CH_3_), 48.3_sp_, 48.8_ap_ (-CH_2_-), 116.9, 124.7, 127.9, 128.3, 129.5, 129.9, 130.6, 131.6, 133.2, 133.8, 134.0, 134.4, 134.5, 139.8, 146.7_sp_ (N=CH), 150.7, 169.2_sp_ (C=O) ppm. Anal. Calcd. for C_20_H_15_C_l__2_N_5_O: C, 58.27; H, 3.67; N, 16.99%. Found: C, 58.41; H, 3.83; N, 17.11%.

*(E)-N’-(4-fluorobenzylidene)-2-(6-methyl-1H-pyrazolo[3,4-b]quinolin-1-yl)acetohydrazide* (**6g**) Yield 89%. Pale yellow solid. m.p. 284–286 °C. FT-IR (ῡ cm^–1^; neat): 3310 (NH), 3030 (CH-Aromatic), 2928 (CH), 1654 (C=O), 1601 (C=N). ^1^H NMR (DMSO-*d_6_*, 300 MHz) *δ* = 2.52_(sp + ap)_ (s, 3H, -CH_3_), 5.34_ap_, 5.75_sp_ (2 s, 2H, -CH_2_-), 7.22–7.32_(sp + ap)_ (m, 2H, H-2′, H-6′), 7.66_(sp + ap)_ (dd, *J* = 9.0 Hz, 1.8 Hz, 1H, H-7), 7.74–7.82_(sp + ap)_ (m, 2H, H-3′, H-5′), 7.92–7.95_(sp + ap)_ (m, 2H, H-5, H-8), 8.06_sp_, 8.27_ap_ (2 s, 1H, N=CH), 8.47_sp_, 8.49_ap_ (s, 1H, H-3), 8.87_sp_, 8.88_ap_ (s, 1H, H-4), 11.76_sp_, 11.89_ap_ (2 s, 1H, NH) ppm. ^13^C NMR (DMSO-*d_6_*, 75 MHz) *δ* = 21.4 (-CH_3_), 48.5_sp_, 48.9_ap_ (-CH_2_-), 116.1, 116.4, 116.9, 124.6, 127.9, 128.3, 129.6, 129.7, 130.6, 133.2, 133.8, 143.5, 146.7_sp_ (N=CH), 150.7, 161.8, 164.0_ap_, 169.0_sp_ (C=O) ppm. Anal. Calcd. for C_20_H_16_FN_5_O: C, 66.47; H, 4.46; N, 19.38%. Found: C, 66.71; H, 4.60; N, 19.62%. 

*(E)-2-(6-methyl-1H-pyrazolo[3,4-b]quinolin-1-yl)-N’-(3-nitrobenzylidene)acetohydrazide* (**6h**) Yield 98%. Off-white solid. m.p. 258–260 °C. FT-IR (ῡ cm^–1^; neat): 3301 (NH), 3040 (CH-Aromatic), 2929 (CH), 1651 (C=O), 1627 (C=N), 1545 (NO_2_ Antisym.), 1313 (NO_2_ Sym.). ^1^H NMR (DMSO-*d_6_*, 300 MHz) *δ* = 2.52_(sp + ap)_ (s, 3H, -CH_3_), 5.38_ap_, 5.80_sp_ (2 s, 2H, -CH_2_-), 7.63–7.73_(sp + ap)_ (m, 2H, H-7, H-5′), 7.92–7.94_(sp + ap)_ (m, 2H, H-5, H-8), 8.13–8.27 (m, 3H, H-4′_(sp + ap)_, H-6′_(sp + ap)_, N=CH_sp_), 8.40_ap_ (s, N=CH), 8.48_sp_, 8.49_ap_ (s, 1H, H-3), 8.53_ap_, 8.56_sp_ (s, 1H, H-2′), 8.87_sp_, 8.88_ap_ (s, 1H, H-4), 11.98_sp_, 12.14_ap_ (2 s, 1H, NH) ppm. ^13^C NMR (DMSO-*d_6_*, 75 MHz) *δ* = 21.4 (-CH_3_), 48.5_sp_, 48.9_ap_ (-CH_2_-), 116.9, 121.8, 124.6, 127.9, 128.3, 130.6, 130.8, 133.2, 133.3, 133.8, 134.0, 134.2, 136.3, 136.4, 142.4, 145.5, 146.7_sp_, 148.6_ap_ (N=CH), 148.7, 150.7, 164.5_ap_, 169.3_sp_ (C=O) ppm. Anal. Calcd. for C_20_H_16_N_6_O_3_: C, 61.85; H, 4.15; N, 21.64%. Found: C, 61.99; H, 4.33; N, 21.80%. 

*(E)-2-(6-methyl-1H-pyrazolo[3,4-b]quinolin-1-yl)-N’-(4-nitrobenzylidene)acetohydrazide* (**6i**) Yield 99%. Off-white solid. m.p. 282–284 °C. FT-IR (ῡ cm^–1^; neat): 3306 (NH), 3031 (CH-Aromatic), 2931 (CH), 1650 (C=O), 1624 (C=N), 1543 (NO_2_ Antisym.), 1307 (NO_2_ Sym.). ^1^H NMR (DMSO-*d_6_*, 300 MHz) *δ* = 2.52_(sp + ap)_ (s, 3H, -CH_3_), 5.39_ap_, 5.79_sp_ (2 s, 2H, -CH_2_-), 7.66_(sp + ap)_ (dd, *J* = 8.4 Hz, 1.5 Hz, 1H, H-7), 7.93–7.99_(sp + ap)_ (m, 4H, H-5, H-8, H-2′, H-6′), 8.22_sp_, 8.29_ap_ (d, *J* = 8.7 Hz, 2H, H-3′, H-5′), 8.15_sp_, 8.38_ap_ (2 s, 1H, N=CH), 8.48_sp_, 8.49_ap_ (s, 1H, H-3), 8.87_sp_, 8.88_ap_ (s, 1H, H-4), 12.04_sp_, 12.19_ap_ (2 s, 1H, NH) ppm. ^13^C NMR (DMSO-*d_6_*, 75 MHz) *δ* = 26.2 (-CH_3_), 53.3_sp_, 53.7_ap_ (-CH_2_-), 121.7, 129.4, 132.6, 133.0, 133.9, 134.1, 134.4, 135.4, 137.9, 138.1, 138.2, 138.5, 138.7, 139.0, 139.6, 139.8, 148.1_sp_, 151.3_ap_ (N=CH), 151.5, 155.4, 168.9_ap_, 173.8_sp_ (C=O) ppm. Anal. Calcd. for C_20_H_16_N_6_O_3_: C, 61.85; H, 4.15; N, 21.64%. Found: C, 61.93; H, 4.39; N, 21.78%.

*(E)-N’-(2-methoxybenzylidene)-2-(6-methyl-1H-pyrazolo[3,4-b]quinolin-1-yl)acetohydrazide* (**6j**) Yield 83%. Off-white solid. m.p. 238–240 °C. FT-IR (ῡ cm^–1^; neat): 3205 (NH), 3005 (CH-Aromatic), 2923 (CH), 1648 (C=O), 1596 (C=N), 1228 (C-O-C). ^1^H NMR (DMSO-*d_6_*, 300 MHz) *δ* = 2.52_(sp + ap)_ (s, 3H, -CH_3_), 3.86_(sp + ap)_ (s, 3H, -OCH_3_), 5.31_ap_, 5.73_sp_ (2 s, 2H, -CH_2_-), 6.93_sp_, 6.96_ap_ (t, *J* = 7.5 Hz, 1H, H-5′), 7.11_(sp + ap)_ (d, *J* = 8.4 Hz, 1H, H-3′), 7.41_(sp + ap)_ (t, *J* = 8.1 Hz, 1H, H-4′), 7.66_(sp + ap)_ (d, *J* = 9.0 Hz, 1H, H-7), 7.78_ap_, 7.85_sp_ (d, *J* = 8.1 Hz, 1H, H-6′), 7.93–7.95_(sp + ap)_ (m, 2H, H-5, H-8), 8.47_sp_, 8.48_ap_ (s, 1H, H-3), 8.40_sp_, 8.61_ap_ (2 s, 1H, N=CH), 8.87_sp_, 8.88_ap_ (s, 1H, H-4), 11.70_sp_, 11.86_ap_ (2 s, 1H, NH) ppm. ^13^C NMR (DMSO-*d_6_*, 75 MHz) *δ* = 21.4 (-CH_3_), 48.5_sp_, 48.9_ap_ (-CH_2_-), 56.2, 112.2, 116.9, 121.1, 122.4, 124.7, 126.0, 127.9, 128.3, 130.6, 132.0, 133.1, 133.8, 133.9, 140.2, 146.7_sp_ (N=CH), 150.7, 158.1, 168.8_sp_ (C=O) ppm. Anal. Calcd. for C_21_H_19_N_5_O_2_: C, 67.55; H, 5.13; N, 18.76%. Found: C, 67.71; H, 5.25; N, 18.88%.

*(E)-N’-(3-methoxybenzylidene)-2-(6-methyl-1H-pyrazolo[3,4-b]quinolin-1-yl)acetohydrazide* (**6k**) Yield 92%. Off-white solid. m.p. 236–238 °C. FT-IR (ῡ cm-1; neat): 3216 (NH), 3010 (CH-Aromatic), 2928 (CH), 1645 (C=O), 1599 (C=N), 1225 (C-O-C). ^1^H NMR (DMSO-*d_6_*, 300 MHz) *δ* = 2.52_(sp + ap)_ (s, 3H, -CH_3_), 3.80_(sp + ap)_ (s, 3H, -OCH_3_), 5.34_ap_, 5.76_sp_ (2 s, 2H, -CH_2_-), 7.00_(sp + ap)_ (ddd, *J* = 7.8 Hz, 2.7 Hz, 1.8 Hz, 1H, H-4′), 7.25–7.36_(sp + ap)_ (m, 3H, H-2′, H-5′, H-6′), 7.65_(sp + ap)_ (dd, *J* = 8.7 Hz, 2.1 Hz, 1H, H-7), 7.92–7.95_(sp + ap)_ (m, 2H, H-5, H-8), 8.04_sp_, 8.24_ap_ (2 s, 1H, N=CH), 8.48_sp_, 8.49_ap_ (s, 1H, H-3), 8.87_sp_, 8.88_ap_ (s, 1H, H-4), 11.77_sp_, 11.89_ap_ (2 s, 1H, NH) ppm. ^13^C NMR (DMSO-*d_6_*, 75 MHz) *δ* = 21.4 (-CH_3_), 48.5_sp_, 48.9_ap_ (-CH_2_-), 55.6 (-OCH_3_), 111.9, 116.6, 116.7, 116.9, 120.2, 120.4, 124.6, 127.9, 128.3, 130.4, 130.6, 130.7, 133.2, 133.3, 133.8, 133.9, 134.2, 135.8, 136.0, 144.5, 146.7_sp_, 147.8_ap_ (N=CH), 150.6, 150.7, 160.0, 164.1_ap_, 169.0_sp_ (C=O) ppm. Anal. Calcd. for C_21_H_19_N_5_O_2_: C, 67.55; H, 5.13; N, 18.76%. Found: C, 67.81; H, 5.37; N, 18.88%.

*(E)-N’-(4-methoxybenzylidene)-2-(6-methyl-1H-pyrazolo[3,4-b]quinolin-1-yl)acetohydrazide* (**6l**) Yield 80%. Off-white solid. m.p. 238–240 °C. FT-IR (ῡ cm^–1^; neat): 3195 (NH), 3019 (CH-Aromatic), 2930 (CH), 1645 (C=O), 1598 (C=N), 1227 (C-O-C). ^1^H NMR (DMSO-*d_6_*, 300 MHz) *δ* = 2.52_(sp + ap)_ (s, 3H, -CH_3_), 3.80_(sp + ap)_ (s, 3H, -OCH_3_), 5.32_ap_, 5.72_sp_ (2 s, 2H, -CH_2_-), 6.96_sp_, 7.00_ap_ (d, *J* = 8.7 Hz, 2H, H-3′, H-5′), 7.65_(sp + ap)_ (d, *J* = 8.7 Hz, 3H, H-7, H-2′, H-6′), 7.93–7.96_(sp + ap)_ (m, 2H, H-5, H-8), 8.00_sp_, 8.21_ap_ (2 s, 1H, N=CH), 8.47_sp_, 8.48_ap_ (s, 1H, H-3), 8.87_sp_, 8.88_ap_ (s, 1H, H-4), 11.61_sp_, 11.73_ap_ (2 s, 1H, NH) ppm. ^13^C NMR (DMSO-*d_6_*, 75 MHz) *δ* = 21.4 (-CH_3_), 48.5_sp_, 48.9_ap_ (-CH_2_-), 55.8 (-OCH_3_), 114.7, 114.8, 116.9, 124.6, 127.0, 127.9, 128.3, 129.0, 129.2, 130.6, 130.7, 133.2, 133.8, 133.9, 134.1, 144.5, 146.7_sp_, 147.7_ap_ (N=CH), 150.6, 150.7, 161.2, 161.4, 163.8_ap_, 168.7_sp_ (C=O) ppm. MS *m*/*z*: 374.26 [M^+^ + 1]. Anal. Calcd. for C_21_H_19_N_5_O_2_: C, 67.55; H, 5.13; N, 18.76 %. Found: C, 67.29; H, 5.00; N, 18.54 %.

*(E)-N’-(3,4-dimethoxybenzylidene)-2-(6-methyl-1H-pyrazolo[3,4-b]quinolin-1-yl)acetohydrazide* (**6m**) Yield 80%. Pale yellow solid. m.p. 232–234 °C. FT-IR (ῡ cm^–1^; neat): 3210 (NH), 3010 (CH-Aromatic), 2929 (CH), 1647 (C=O), 1596 (C=N), 1225 (C-O-C). ^1^H NMR (DMSO-*d_6_*, 300 MHz) *δ* = 2.52_(sp + ap)_ (s, 3H, -CH_3_), 3.78_(sp + ap)_, 3.80_(sp + ap)_ (2 overlapping s, 6H, -OCH_3_), 5.33ap, 5.76sp (2 s, 2H, -CH_2_-), 6.99sp, 7.02ap (d, *J* = 8.4 Hz, 1H, H-5′), 7.21_(sp + ap)_ (dd, *J* = 8.4 Hz, 1.8 Hz, 1H, H-6′), 7.29_ap_, 7.41_sp_ (d, *J* = 1.8 Hz, 1H, H-2′), 7.65_(sp + ap)_ (dd, *J* = 8.7 Hz, 2.1 Hz, 1H, H-7), 7.92–7.95_(sp + ap)_ (m, 2H, H-5, H-8), 7.99_sp_, 8.19_ap_ (2 s, 1H, N=CH), 8.47_sp_, 8.48_ap_ (s, 1H, H-3), 8.87_(sp + ap)_ (s, 1H, H-4), 11.66_sp_, 11.76_ap_ (2 s, 1H, NH) ppm. ^13^C NMR (DMSO-*d_6_*, 75 MHz) *δ* = 21.4 (-CH_3_), 48.6_sp_, 48.9_ap_ (-CH_2_-), 55.9 (-OCH_3_), 56.0 (-OCH_3_), 108.9, 111.9, 116.9, 121.9, 122.3, 124.6, 127.2, 127.9, 128.3, 130.6, 133.2, 133.8, 144.7, 146.7_sp_, 148.0_ap_ (N=CH), 149.5, 150.6, 150.7, 151.1, 151.2, 163.8_ap_, 168.8_sp_ (C=O) ppm. Anal. Calcd. for C_22_H_21_N_5_O_3_: C, 65.50; H, 5.25; N, 17.36%. Found: C, 65.76; H, 5.51; N, 17.60%.

*(E)-2-(6-methyl-1H-pyrazolo[3,4-b]quinolin-1-yl)-N’-(3,4,5-trimethoxybenzylidene)acetohydrazide* (**6n**) Yield 85%. Off-white solid. Decomposes at 254 °C. FT-IR (ῡ cm^–1^; neat): 3209 (NH), 3005 (CH-Aromatic), 2927 (CH), 1643 (C=O), 1596 (C=N), 1227 (C-O-C). ^1^H NMR (DMSO-*d_6_*, 300 MHz) *δ* = 2.52_(sp + ap)_ (s, 3H, -CH_3_), 3.70_ap_, 3.71_sp_ (s, 3H, -OCH_3_), 3.81_ap_, 3.83_sp_ (s, 6H, -OCH_3_), 5.35_ap_, 5.78_sp_ (2 s, 2H, -CH_2_-), 7.01_ap_, 7.10_sp_ (2 s, 2H, H-2′, H-6′), 7.65_(sp + ap)_ (dd, *J* = 9.0 Hz, 1.8 Hz, 1H, H-7), 7.92–7.94_(sp + ap)_ (m, 2H, H-5, H-8), 8.00_sp_, 8.20_ap_ (s, 1H, N=CH), 8.48_(sp + ap)_ (s, 1H, H-3), 8.88_(sp + ap)_ (s, 1H, H-4), 11.79_sp_, 11.86_ap_ (2 s, 1H, NH) ppm. ^13^C NMR (DMSO-*d_6_*, 75 MHz) *δ* = 21.4 (-CH_3_), 48.6_sp_, 49.0_ap_ (-CH_2_-), 56.4 (-OCH_3_), 60.6 (-OCH_3_), 104.7, 104.8, 116.9, 124.6, 127.9, 128.3, 130.0, 130.6, 133.2, 133.8, 133.9, 139.5, 144.5, 146.7_sp_, 147.8_ap_ (N=CH), 150.7, 153.6, 164.0_ap_, 169.0_sp_ (C=O) ppm. Anal. Calcd. for C_23_H_23_N_5_O_4_: C, 63.73; H, 5.35; N, 16.16%. Found: C, 63.65; H, 5.27; N, 16.06%.

*(E)-N’-(4-(dimethylamino)benzylidene)-2-(6-methyl-1H-pyrazolo[3,4-b]quinolin-1-yl)acetohydrazide* (**6o**) Yield 80%. Pale yellow solid. m.p. 260–262 °C. FT-IR (ῡ cm^–1^; neat): 3198 (NH), 3030 (CH-Aromatic), 2927 (CH), 1651 (C=O), 1601 (C=N). ^1^H NMR (DMSO-*d_6_*, 300 MHz) *δ* = 2.52_(sp + ap)_ (s, 3H, -CH_3_), 2.96_(sp + ap)_ (s, 6H, N-CH_3_), 5.29_ap_, 5.69_sp_ (2 s, 2H, -CH_2_-), 6.68_sp_, 6.74_ap_ (d, *J* = 9.0 Hz, 2H, H-3′, H-5′), 7.47–7.52_(sp + ap)_ (m, 2H, H-2′, H-6′), 7.66_(sp + ap)_ (dd, *J* = 8.7 Hz, 1.8 Hz, 1H, H-7), 7.91–7.93_(sp + ap)_ (m, 2H, H-5, H-8), 7.96_sp_, 8.12_ap_ (2 s, 1H, N=CH), 8.46_sp_, 8.48_ap_ (s, 1H, H-3), 8.86_sp_, 8.87_ap_ (s, 1H, H-4), 11.44_sp_, 11.55_ap_ (2 s, 1H, NH) ppm. ^13^C NMR (DMSO-*d_6_*, 75 MHz) *δ* = 21.4 (-CH_3_), 48.5_sp_, 48.9_ap_ (-CH_2_-), 112.2, 116.9, 121.8, 124.6, 127.9, 128.3, 128.7, 128.9, 130.6, 133.1, 133.2, 133.7, 133.8, 145.4, 146.7_sp_, 148.6_ap_ (N=CH), 150.7, 151.9, 163.4_ap_, 168.3_sp_ (C=O) ppm. MS *m*/*z*: 387.34 [M^+^ + 1]. Anal. Calcd. for C_22_H_22_N_6_O: C, 68.38; H, 5.74; N, 21.75%. Found: C, 68.50; H, 5.92; N, 21.91%.

*(E)-N’-(4-(diethylamino)benzylidene)-2-(6-methyl-1H-pyrazolo[3,4-b]quinolin-1-yl)acetohydrazide* (**6p**) Yield 80%. Pale yellow solid. m.p. 208–210 °C. FT-IR (ῡ cm^–1^; neat): 3216 (NH), 3032 (CH-Aromatic), 2969, 2928 (CH), 1661 (C=O), 1603 (C=N). ^1^H NMR (DMSO-*d_6_*, 300 MHz) *δ* = 1.09_(sp + ap)_ (t, *J* = 6.9 Hz, 6H, -CH_2_-CH_3_), 2.52(sp + ap) (s, 3H, -CH_3_), 3.36_(sp + ap)_ (q, *J* = 6.9 Hz, 4H, -CH_2_-CH_3_), 5.29_ap_, 5.67_sp_ (2 s, 2H, -CH_2_-), 6.59_sp_, 6.68_ap_ (d, *J* = 8.7 Hz, 2H, H-3′, H-5′), 7.40_sp_, 7.47_ap_ (d, *J* = 8.7 Hz, 2H, H-2′, H-6′), 7.66_(sp + ap)_ (dd, *J* = 8.7 Hz, 1.8 Hz, 1H, H-7), 7.94–7.97_(sp + ap)_ (m, 2H, H-5, H-8), 7.86_sp_, 8.08_ap_ (2 s, 1H, N=CH), 8.46_sp_, 8.48_ap_ (s, 1H, H-3), 8.86_sp_, 8.87_ap_ (s, 1H, H-4), 11.41_sp_, 11.53_ap_ (2 s, 1H, NH) ppm. ^13^C NMR (DMSO-*d_6_*, 75 MHz) *δ* = 12.9 (-CH_2_-CH_3_), 21.5 (-CH_3_), 44.2 (-CH_2_-CH_3_), 48.6_sp_, 48.9_ap_ (-CH_2_-), 111.4, 116.9, 120.8, 124.7, 127.9, 128.3, 129.0, 129.3, 130.6, 133.1, 133.7, 133.8, 145.3, 146.7_sp_, 148.6_ap_ (N=CH), 149.1, 150.7, 164.2_ap_, 168.3_sp_ (C=O) ppm. Anal. Calcd. for C_24_H_26_N_6_O: C, 69.54; H, 6.32; N, 20.27%. Found: C, 69.78; H, 6.50; N, 20.43%.

*(E)-N’-(2-hydroxybenzylidene)-2-(6-methyl-1H-pyrazolo[3,4-b]quinolin-1-yl)acetohydrazide* (**6q**) Yield 87%. Off-white solid. m.p. 262–264 °C. FT-IR (ῡ cm^–1^; neat): 3416 (-OH), 3276 (NH), 3040 (CH-Aromatic), 2921 (CH), 1658 (C=O), 1601 (C=N). ^1^H NMR (DMSO-*d_6_*, 300 MHz) *δ* = 2.52_(sp + ap)_ (s, 3H, -CH_3_), 5.36_ap_, 5.72_sp_ (2 s, 2H, -CH_2_-), 6.91_sp_ (t, *J* = 7.5 Hz, 1H, H-5′), 6.88–6.93 (m, 1H, H-3′_(sp + ap)_, H-5′_ap_), 7.22–7.32_(sp + ap)_ (m, 1H, H-4′), 7.55_ap_, 7.73_sp_ (2 dd, *J* = 7.8 Hz, 1.5 Hz, 1H, H-6′), 7.64–7.68_(sp + ap)_ (m, 1H, H-7), 7.93–7.96_(sp + ap)_ (m, 2H, H-5, H-8), 8.38_sp_, 8.48_ap_ (2 s, 1H, N=CH), 8.47_sp_, 8.49_ap_ (s, 1H, H-3), 8.86_sp_, 8.88_ap_ (s, 1H, H-4), 10.05_sp_, 10.95_ap_ (2 s, 1H, -OH), 11.65_sp_, 12.06_ap_ (2 s, 1H, NH) ppm. ^13^C NMR (DMSO-*d_6_*, 75 MHz) *δ* = 21.4 (-CH_3_), 48.5_sp_, 48.8_ap_ (-CH_2_-), 116.6, 116.8, 116.9, 119.1, 119.8, 120.6, 124.6, 124.7, 126.7, 127.9, 128.3, 129.6, 130.6, 130.8, 131.7, 132.0, 133.2, 133.3, 133.8, 133.9, 134.3, 141.8, 146.7_sp_, 148.0_ap_ (N=CH), 150.6, 150.7, 156.9, 157.8, 164.0_ap_, 168.6_sp_ (C=O) ppm. Anal. Calcd. for C_20_H_17_N_5_O_2_: C, 66.84; H, 4.77; N, 19.49%. Found: C, 67.02; H, 4.95; N, 19.63%.

*(E)-4-((2-(2-(6-methyl-1H-pyrazolo[3,4-b]quinolin-1-yl)acetyl)hydrazono)methyl)benzoic acid* (**6r**) Yield 90%. Off-white solid. Decomposes at 302 °C. FT-IR (ῡ cm^–1^; neat): 3434 (-OH), 3223 (NH), 3053 (CH-Aromatic), 2927 (CH), 1741 (C=O, Carboxyl), 1652 (C=O), 1619 (C=N). ^1^H NMR (DMSO-*d_6_*_,_ 300 MHz) *δ* = 2.52_(sp + ap)_ (s, 3H, -CH_3_), 5.37_ap_, 5.77_sp_ (2 s, 2H, -CH_2_-), 7.66_(sp + ap)_ (dd, *J* = 9.0 Hz, 1.8 Hz, 1H, H-7), 7.82_(sp + ap)_ (d, *J* = 8.4 Hz, 2H, H-2′, H-6′), 7.93–8.01_(sp + ap)_ (m, 4H, H-5, H-8, H-3′, H-5′), 8.11_sp_, 8.32_ap_ (2 s, 1H, N=CH), 8.48_sp_, 8.49_ap_ (s, 1H, H-3), 8.87_sp_, 8.88_ap_ (s, 1H, H-4), 11.89_sp_, 12.04_ap_ (s, 1H, NH), 13.03_(sp + ap)_ (s, 1H, -COOH) ppm. ^13^C NMR (DMSO-*d6*, 75 MHz) *δ* = 21.4 (-CH_3_), 48.6_sp_, 49.0_ap_ (-CH_2_-), 116.9, 124.7, 127.5, 127.6, 127.9, 128.3, 130.2, 130.7, 132.1, 132.2, 133.2, 133.3, 133.8, 134.0, 134.2, 138.4, 138.6, 143.5, 146.7_sp_ (N=CH), 150.6, 150.7, 167.3 (C=O of COOH), 164.3_ap_, 169.2_sp_ (C=O) ppm. Anal. Calcd. for C_21_H_17_N_5_O_3_: C, 65.11; H, 4.42; N, 18.08%. Found: C, 65.19; H, 4.46; N, 18.14%.

*(E)-N-(4-((2-(2-(6-methyl-1H-pyrazolo[3,4-b]quinolin-1-yl)acetyl)hydrazono)methyl)phenyl)acetamide* (**6s**) Yield 86%. Off-white solid. m.p. 286–288 °C. FT-IR (ῡ cm^–1^; neat): 3305 (NH), 3235 (NH), 3041 (CH-Aromatic), 2929 (CH), 1651 (C=O), 1611 (C=N). ^1^H NMR (DMSO-*d_6_*, 300 MHz) *δ* = 2.06_(sp + ap)_ (s, 3H, C(O)-CH_3_), 2.52(sp + ap) (s, 3H, -CH_3_), 5.32ap, 5.73sp (2 s, 2H, -CH_2_-), 7.62–7.67_(sp + ap)_ (m, 5H, H-7, H-2′, H-3′, H-5′, H-6′), 7.93–7.95_(sp + ap)_ (m, 2H, H-5, H-8), 8.00_sp_, 8.20_ap_ (2 s, 1H, N=CH), 8.47_sp_, 8.48_ap_ (s, 1H, H-3), 8.87_(sp + ap)_ (s, 1H, H-4), 10.13_(sp + ap)_ (s, 1H, Ar-NH), 11.65_sp_, 11.78_ap_ (2 s, 1H, NH) ppm. ^13^C NMR (DMSO-*d_6_*, 75 MHz) *δ* = 21.5 (-CH_3_), 24.6 (C(O)-CH_3_), 48.5_sp_, 48.9_ap_ (-CH_2_-), 116.9, 119.3, 124.6, 127.9, 128.2, 128.3, 129.0, 130.6, 133.2, 133.8, 133.9, 141.4, 144.4, 146.7_sp_, 147.6_ap_ (N=CH), 150.7, 163.8_ap_, 168.8_sp_ (C=O), 169.0 (C=O)ppm. Anal. Calcd. for C_22_H_20_N_6_O_2_: C, 65.99; H, 5.03; N, 20.99%. Found: C, 66.17; H, 5.25; N, 21.13%.

*(E)-2-(6-methyl-1H-pyrazolo[3,4-b]quinolin-1-yl)-N’-(pyridin-3-ylmethylene)acetohydrazide* (**6t**) Yield 83%. Off-white solid. m.p. 248–250 °C. FT-IR (ῡ cm^–1^; neat): 3302 (NH), 3040 (CH-Aromatic), 2920 (CH), 1656 (C=O), 1606 (C=N), 1571 (C=N, Pyridine). ^1^H NMR (DMSO-*d_6_*, 300 MHz) *δ* = 2.52_(sp + ap)_ (s, 3H, -CH_3_), 5.36_ap_, 5.77_sp_ (2 s, 2H, -CH_2_-), 7.41–7.49_(sp + ap)_ (m, 1H, H-5′), 7.65_(sp + ap)_ (dd, *J* = 9.0, 1.8 Hz, 1H, H-7), 7.92–7.95_(sp + ap)_ (m, 2H, H-5, H-8), 8.16_(sp + ap)_ (dt, *J* = 8.1 Hz, 1.8 Hz, 1H, H-6′), 8.11_sp_, 8.33_ap_ (2 s, 1H, N=CH), 8.48_sp_, 8.49_ap_ (s, 1H, H-3), 8.61_(sp + ap)_ (dd, *J* = 4.8 Hz, 1.8 Hz, 1H, H-4′), 8.87_sp_, 8.88_ap_ (s, 1H, H-4), 8.84_ap_, 8.91_sp_ (d, *J* = 1.8 Hz, 1H, H-2′), 11.93_(sp + ap)_ (s, 1H, NH) ppm. ^13^C NMR (DMSO-*d_6_*, 75 MHz) *δ* = 21.4 (-CH_3_), 48.5_sp_, 48.9_ap_ (-CH_2_-), 116.9, 124.3, 124.4, 124.7, 127.9, 128.3, 130.4, 130.5, 130.6, 130.7, 133.2, 133.3, 133.8, 133.9, 134.0, 134.2, 141.9, 145.2, 146.7_sp_, 149.2_ap_ (N=CH), 149.3, 150.6, 150.7, 151.1, 151.3, 164.3_ap_, 169.2_sp_ (C=O) ppm. Anal. Calcd. for C_19_H_16_N_6_O: C, 66.27; H, 4.68; N, 24.40%. Found: C, 66.41; H, 4.80; N, 24.56%.

## 4. Conclusions

In summary, we have described the synthesis and characterization of novel *N’*-(benzylidene)-2-(6-methyl-1*H*-pyrazolo[3,4-*b*]quinolin-1-yl)acetohydrazides. The compounds were synthesized by a facile route using multistep synthesis approach and their structures were established by different spectroscopic techniques. This study illustrates that duplicated signals observed in NMR spectra of *N’*-(benzylidene)-2-(6-methyl-1*H*-pyrazolo[3,4-*b*]quinolin-1-yl)acetohydrazides correspond to the presence of two amide bond-related conformers. The consistency of the combined results of the obtained ^1^H NMR and ^13^C NMR spectra to the previously reported literature has revealed that the *E*_(C=N)_ configurational isomers of **6**(**a**–**t**) rapidly establish a *synperiplanar*/*antiperiplanar* equilibrium about amide bond in DMSO-*d_6_* solution. The ratio of *sp*/*ap* rotational stereoisomers of **6**(**a**–**t**) is determined on the basis of the investigation of ^1^H as well as ^13^C NMR spectra at room temperature. The *synperiplanar* conformer, being more stable, predominates the *antiperiplanar* isomer due to its ability to develop intermolecular interactions with the polar solvent, i.e., DMSO. The rotational isomers arise from the increase in rotational barriers among them, which results from the steric and electronic factors in the molecules.

## Figures and Tables

**Table 1:** Chemical shifts of duplicated peaks in ^1^H NMR spectra of compounds **6**(**a–t**) in DMSO-*d_6_*.

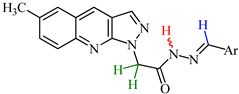							
Compound	Chemical Shift *δ* (ppm)						
	C(O)-NH		N=CH		CH_2_-CO		Ar-H ^1^ Range
	*syn-*	*anti-*	*syn-*	*anti-*	*syn-*	*anti-*	
**6a**	11.75	11.88	8.06	8.28	5.75	5.35	7.64–8.56
**6b**	11.92	12.11	8.45	8.66	5.77	5.35	7.30–8.50
**6c**	11.86	12.02	8.04	8.26	5.77	5.36	7.41–7.95
**6d**	11.81	11.95	8.05	8.26	5.75	5.35	7.45–7.95
**6e**	11.96	12.16	8.38	8.60	5.77	5.35	7.37–8.04
**6f**	12.04	12.18	8.35	8.46 ^2^	5.67	5.38	7.41–7.97
**6g**	11.76	11.89	8.06	8.27	5.75	5.34	7.22–7.95
**6h**	11.99	12.15	--- ^2^	8.40 ^2^	5.80	5.38	7.63–8.56
**6i**	12.04	12.19	8.15	8.38	5.79	5.39	7.65–8.22
**6j**	11.70	11.86	8.40	8.61	5.73	5.31	6.91–7.95
**6k**	11.77	11.89	8.04	8.24	5.76	5.34	6.98–7.95
**6l**	11.61	11.73	8.00	8.21	5.72	5.32	6.94–7.96
**6m**	11.66	11.76	7.99	8.19	5.76	5.33	6.98–7.95
**6n**	11.79	11.86	8.00	8.20	5.78	5.35	7.01–7.94
**6o**	11.44	11.55	7.96 ^2^	8.12	5.69	5.29	6.66–7.96
**6p**	11.41	11.53	7.86	8.08	5.67	5.29	6.57–7.97
**6q**	11.65	12.06	8.38	8.48 ^2^	5.72	5.36	6.78–8.00
**6r**	11.89	12.04	8.11	8.33	5.77	5.37	7.64–8.01
**6s**	11.65	11.78	8.00	8.20	5.73	5.32	7.62–7.95
**6t**	11.93	--- ^3^	8.11 ^2^	8.33	5.77	5.36	7.39–8.27

^1^ Aryl protons excluding H-3 and H-4 of pyrazoloquinoline. ^2^ Signal overlapped by aromatic signals. ^3^ Signal not detected.

**Table 2:** Ratio of conformers of **6**(**a**–**t**) in DMSO-*d_6_* *.

Compound	*syn-* Conformer		*anti-* Conformer		Percentage Isomeric Ratio (*syn*-:*anti*-)
	*δ* (ppm)	Height	*δ* (ppm)	Height	
**6a**	5.75	1.92	5.35	0.62	75.6:24.4
**6b**	5.77	1.96	5.35	0.62	76.0:24.0
**6c**	5.77	2.06	5.36	0.66	75.7:24.3
**6d**	5.75	2.02	5.35	0.65	75.7:24.3
**6e**	5.77	1.89	5.35	0.66	74.1:25.9
**6f**	5.67	2.03	5.38	0.50	80.2:19.8
**6g**	5.75	1.91	5.34	0.63	75.2:24.8
**6h**	5.80	1.00	5.38	0.40	71.4:28.6
**6i**	5.79	1.50	5.39	0.45	76.9:23.1
**6j**	5.73	1.50	5.31	0.43	77.7:22.3
**6k**	5.76	1.52	5.34	0.48	76.0:24.0
**6l**	5.72	1.46	5.32	0.50	74.5:25.5
**6m**	5.76	1.99	5.33	0.69	74.3:25.7
**6n**	5.78	1.47	5.35	0.48	75.4:24.6
**6o**	5.69	1.42	5.29	0.54	72.4:27.6
**6p**	5.67	1.41	5.29	0.49	74.2:25.8
**6q**	5.72	1.26	5.36	0.98	56.2:43.8
**6r**	5.77	1.45	5.37	0.47	75.5:24.5
**6s**	5.73	1.55	5.32	0.44	77.9:22.1
**6t**	5.77	1.58	5.36	0.56	73.8:26.2

* Calculated from relative integration of -CH_2_- paired peak.

**Table 3:** Data of distinct peaks in ^13^C NMR spectra of compounds **6**(**a**–**t**) in DMSO-*d_6_*.

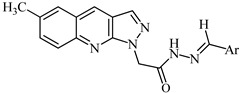							
Compound	Chemical Shift *δ* (ppm)						
	C(O)-NH		N=CH		CH_2_-CO		Ar-C Range
	*syn-*	*anti-*	*syn-*	*anti-*	*syn-*	*anti-*	
**6a**	169.0	164.1	146.7	147.9	48.5	48.9	116.9–150.7
**6b**	169.1	164.3	146.7	--	48.6	49.0	116.9–150.7
**6c**	169.2	164.3	146.7	--	48.6	48.9	116.9–150.7
**6d**	169.3	163.7	146.7	147.5	48.5	48.8	116.7–150.5
**6e**	169.2	164.3	146.7	--	48.5	--	116.9–150.7
**6f**	169.2	--	146.7	--	48.3	48.8	116.9–150.7
**6g**	169.0	164.0	146.7	--	48.5	48.9	116.1–161.8
**6h**	169.2	164.5	146.7	148.6	48.5	48.9	116.9–150.7
**6i**	173.8	168.9	148.1	151.3	53.7	53.3	121.7–155.4
**6j**	168.8	164.3	146.7	---	48.5	48.9	112.2–158.1
**6k**	169.0	164.1	146.7	147.8	48.5	48.9	111.9–150.7
**6l**	168.7	163.8	146.7	147.7	48.5	48.9	114.7–161.4
**6m**	168.8	163.8	146.7	148.0	48.6	48.9	108.9–151.2
**6n**	169.0	164.0	146.7	147.8	48.6	49.0	104.7–153.7
**6o**	168.3	163.4	146.7	148.6	48.5	48.9	112.2–151.9
**6p**	168.3	164.2	146.7	148.7	48.6	48.9	111.4–150.7
**6q**	168.6	164.0	146.7	148.0	48.5	48.8	116.6–157.8
**6r**	169.2	164.3	146.7	150.6	48.6	49.0	116.9–167.3
**6s**	169.0	163.8	146.7	147.6	48.5	48.9	116.9–150.7
**6t**	169.2	164.3	146.7	145.2	48.6	48.9	116.9–151.3

## Data Availability

The data presented in this study are available in Supplementary Material.

## Supplementary Materials

The following are available online. Experimental procedures for compounds **1** and **2**, NMR spectra (^1^H NMR and ^13^C NMR) of all the synthesized compounds and mass spectra of representative compounds.

## Sample Availability

Samples of the synthesized *N*-acylhydrazone compounds are available from the authors on justified request.
