# Supplementary material for: Synthesis of Novel N-Acylhydrazones and Their C-N/N-N Bond Conformational Characterization by NMR Spectroscopy
Source: Molecules. 2021 Aug 13;26(16):4908. doi: 10.3390/molecules26164908 (PMC8399016; doi:10.3390/molecules26164908)
Supplement: Supplementary file 1 [file molecules-26-04908-s001.zip › molecules-1335711-SI.pdf]

Supplementary Information

Synthesis of Novel *N*-Acylhydrazones and their C-N / N-N Bond Conformational Characterization by NMR Spectroscopy

Rubina Munir[1,2,*], Noman Javid[3], Muhammad Zia-ur-Rehman[4,*], Muhammad Zaheer[4], Rahila Huma[2], Ayesha Roohi[2] and Muhammad Makshoof Athar[1]

[1]Institute of Chemistry, University of the Punjab, Lahore 54590, Pakistan; organist94@gmail.com (R.M.); atharmakshoof@gmail.com (M.M.A.)

[2]Department of Chemistry, Kinnaird College for Women, Lahore 54000, Pakistan organist94@gmail.com (R.M.); rahila.huma@kinnaird.edu.pk (R.H.); ayesha.roohi@kinnaird.edu.pk (A.R.)

[3]Applied Chemistry Research Centre, PCSIR Laboratories Complex, Lahore 54600, Pakistan; rehman_pcsir@yahoo.com (M.Z.R.)

[4]Department of Chemistry (C-Block), Forman Christian College, Ferozepur Road Lahore, Pakistan; noumanjavid@gmail.com (N.J.)

***** Correspondence: organist94@gmail.com, rubina.munir@kinnaird.edu.pk (R.M.); rehman_pcsir@yahoo.com (M.Z.R.)

Supplementary Information

Procedures for the Synthesis of 1and 2

Method for Synthesis of 2-Chloro-6-methylquinoline-3-carbaldehyde (1)

2-Chloro-6-methylquinoline-3-carbaldehyde (**1**) was prepared by using method reported by Meth-Cohn and coworkers [1]. $POCl_3$ (65.3 mL, 107.45 g, 0.70 mol) was added dropwise to DMF (19.3 mL, 18.26 g, 0.25 mol) with constant stirring while maintaining the temperature of the flask at 0°C. To the resulting Vilsmeyer reagent, *N*-(*p*-tolyl)acetamide (0.10 mol) was added and the reaction mixture was heated at 70 – 80 °C for 18h. The reaction mixture was poured on crushed ice (500 g) vigilantly and stirred vigorously at 0 – 10 °C for 30 minutes. The precipitated product **1** was filtered, washed with excess water, dried and recrystallized from ethyl acetate.

Yield 75 %. Yellow solid. Mp 123 – 124 °C. ^1H NMR (300 MHz, $CDCl_3$) δ = 2.58 (s, 3H), 7.71 – 7.74 (m, 2H), 7.98 (d, *J* = 8.4 Hz, 1H), 8.68 (s, 1H), 10.56 (s, 1H) ppm.

Method for Synthesis of 6-Methyl-1H-pyrazolo[3,4-b]quinoline (2)

The synthetic method reported by Mali and coworkers was followed for the synthesis of 1*H*-pyrazolo[3,4-*b*]quinolines [2]. A mixture of 2-chloro-6-methylquinoline-3-carbaldehyde (**1**) (10 mmol) and hydrazine monohydrate (98%, 1.6 mL, 30 mmol) in water was heated at reflux temperature with stirring. After completion of the reaction, the reaction mixture was allowed to attain room temperature. The desired product was filtered, washed with water, dried and recrystallized using ethanol.

Yield 90%. Yellow solid. Mp 176 – 177 °C. ^1H NMR (300 MHz, DMSO-d$_6$) δ = 2.50 (s, 3H), 7.62 (dd, *J* = 8.7 Hz, 1.8 Hz, 1H), 7.87 – 7.91 (m, 2H), 8.39 (s, 1H), 8.80 (s, 1H), 13.50 (s, 1H) ppm. ^{13}C NMR (75 MHz, DMSO-*d*$_6$) δ = 18.45, 115.51, 123.00, 123.77, 127.64, 130.01, 130.76, 133.91, 134.84, 146.94, 151.14 ppm. Anal. Calcd. for $C_{11}H_9N_3$: C, 72.11; H, 4.95; N, 22.94 % Found: C, 72.23; H, 5.04; N, 23.01 %.

Supplementary Information

Spectra of Synthesized Compounds

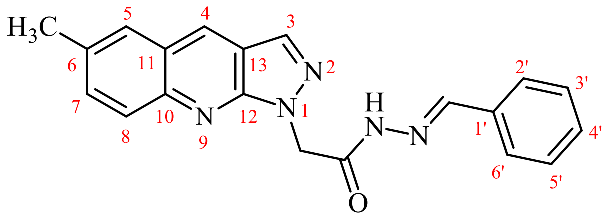

Figure S1. Numbering of Carbon atoms for Compounds **6**(**a-t**)

Supplementary Information

Spectra of 3:

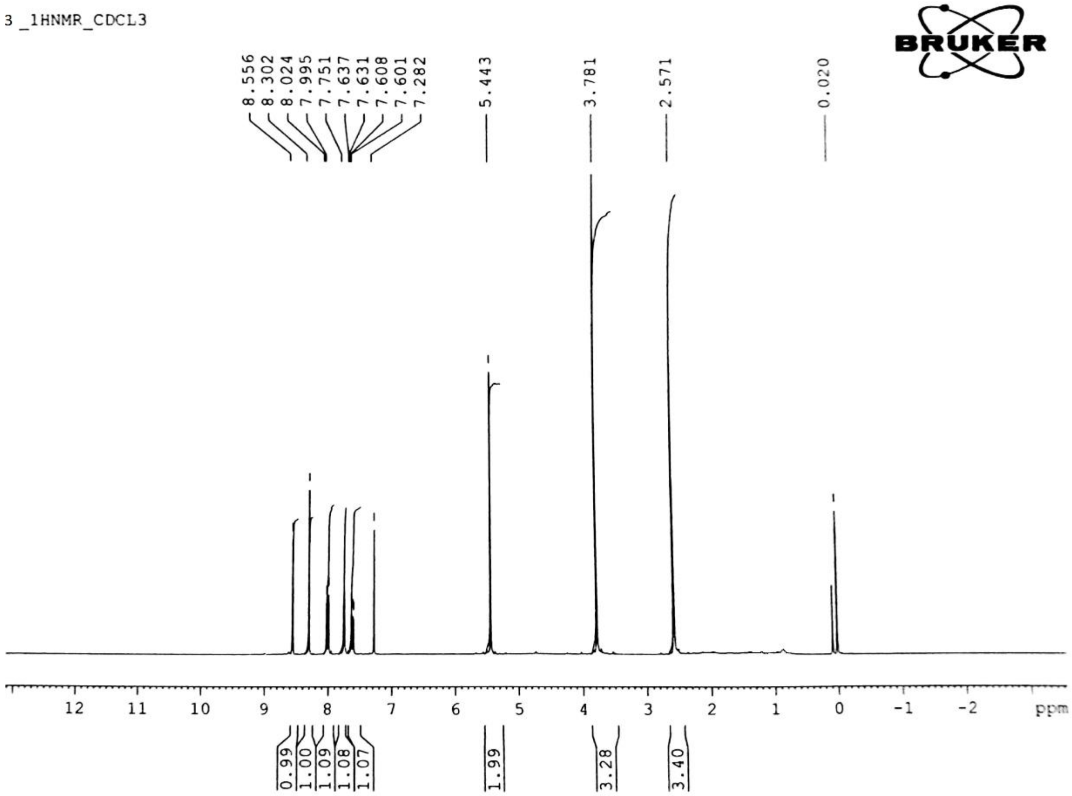

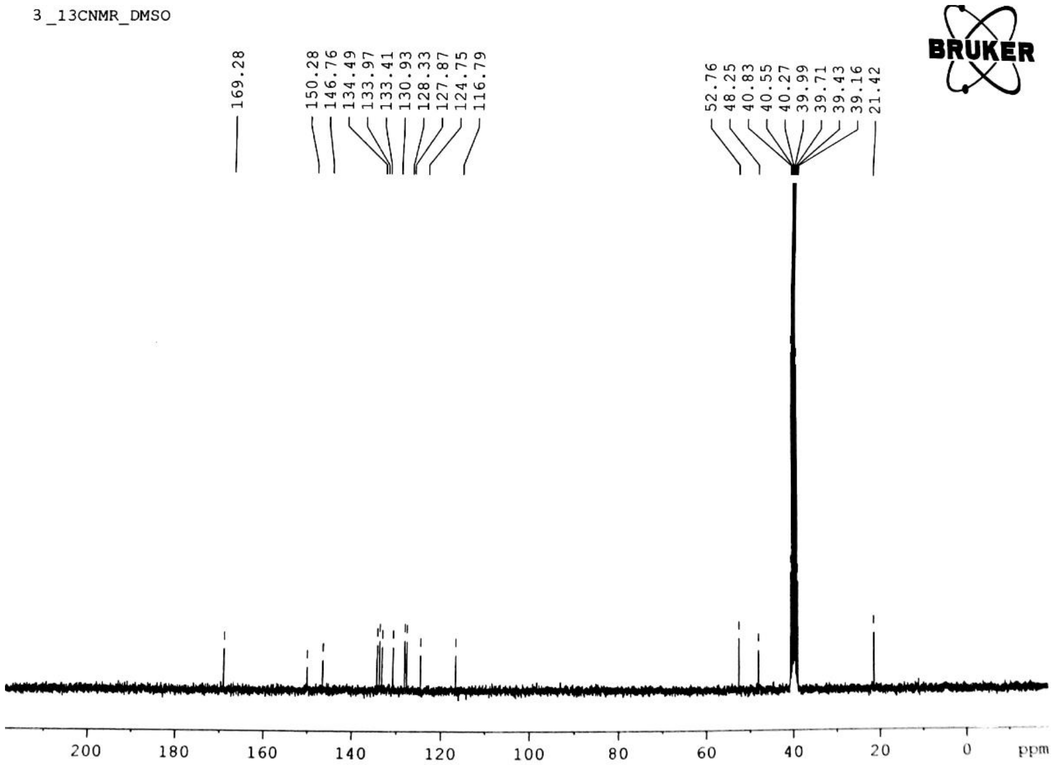

Supplementary Information

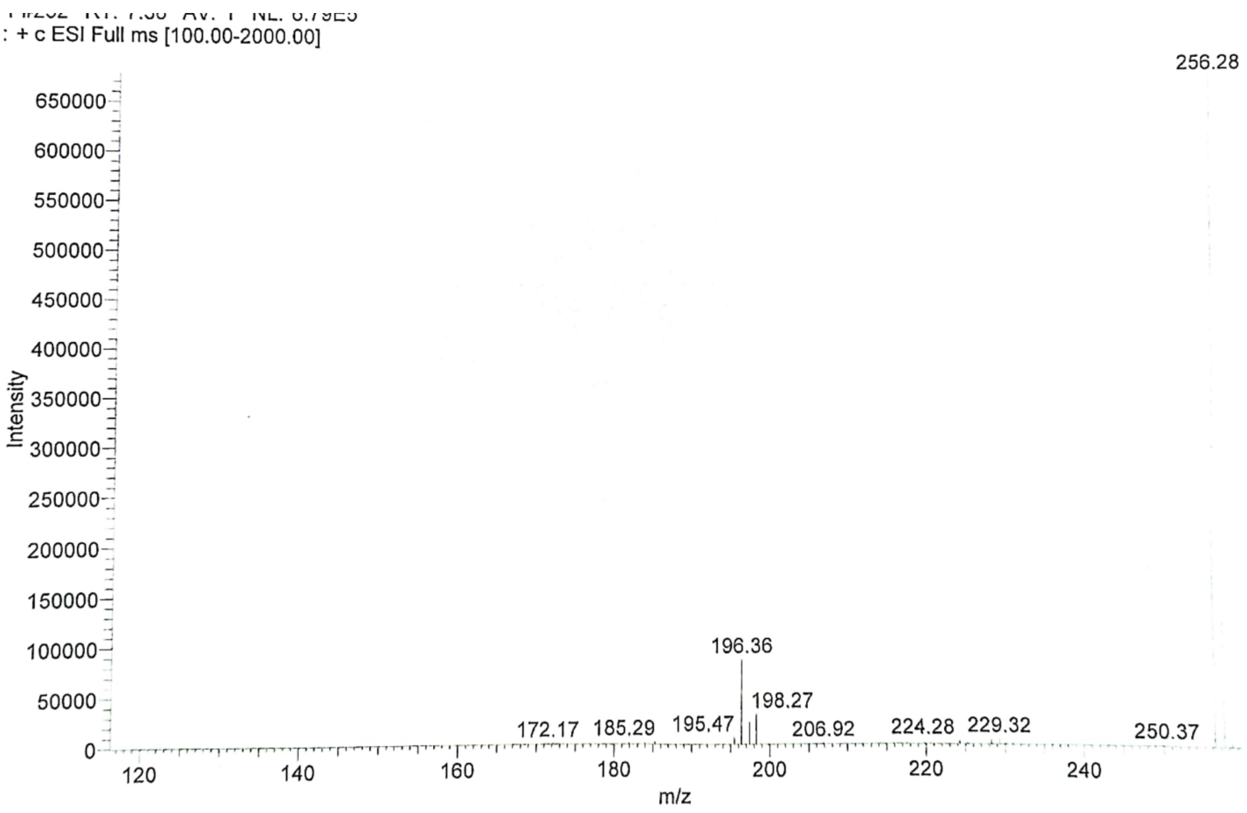

Supplementary Information

Spectra of 4:

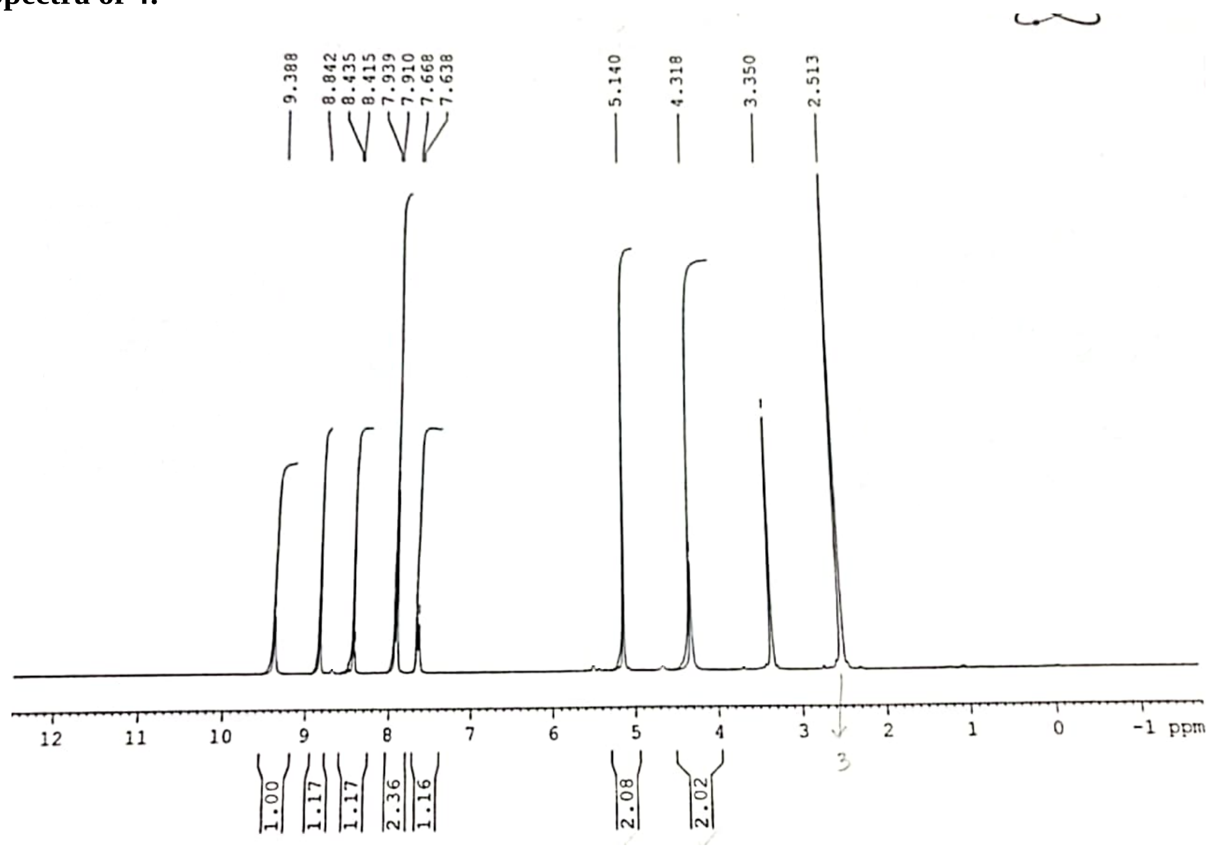

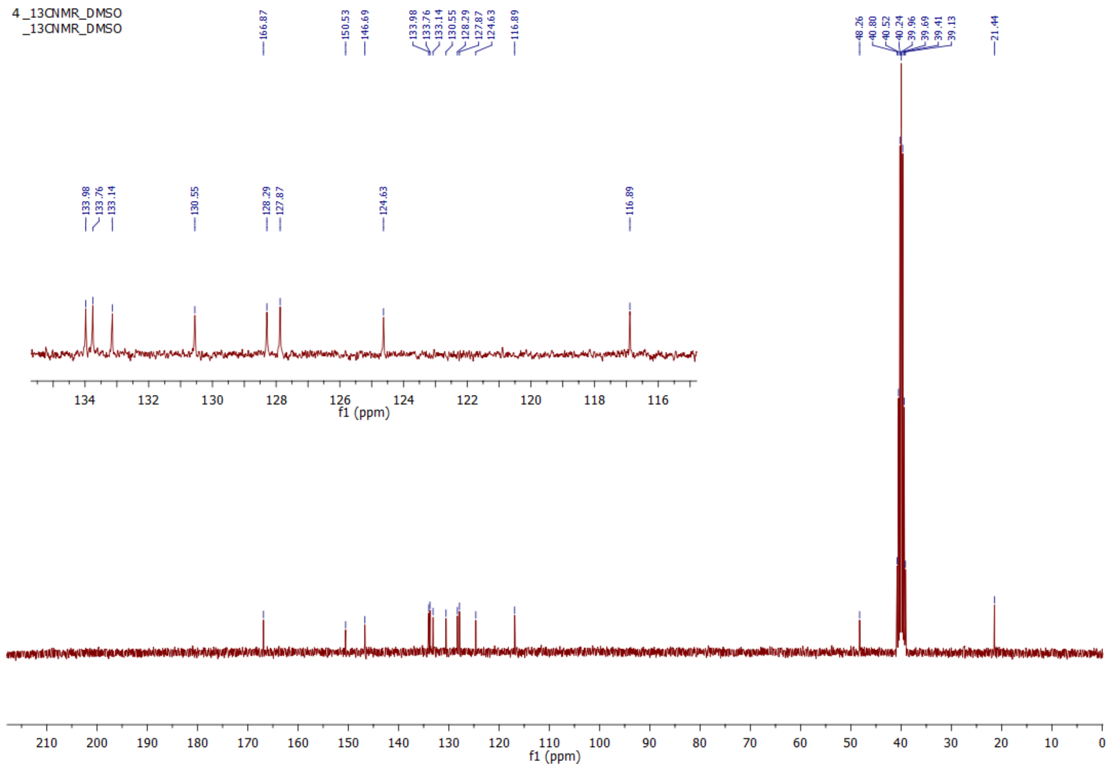

Supplementary Information

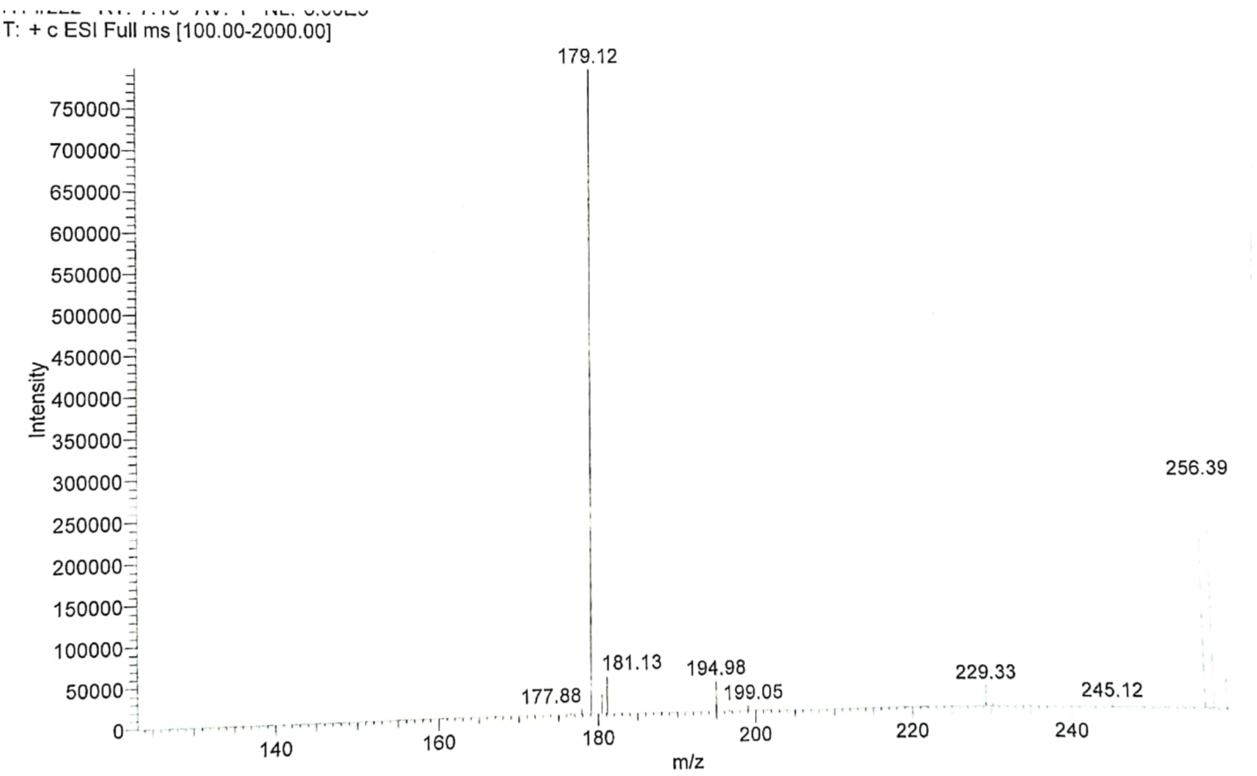

Supplementary Information

Spectra of 6a:

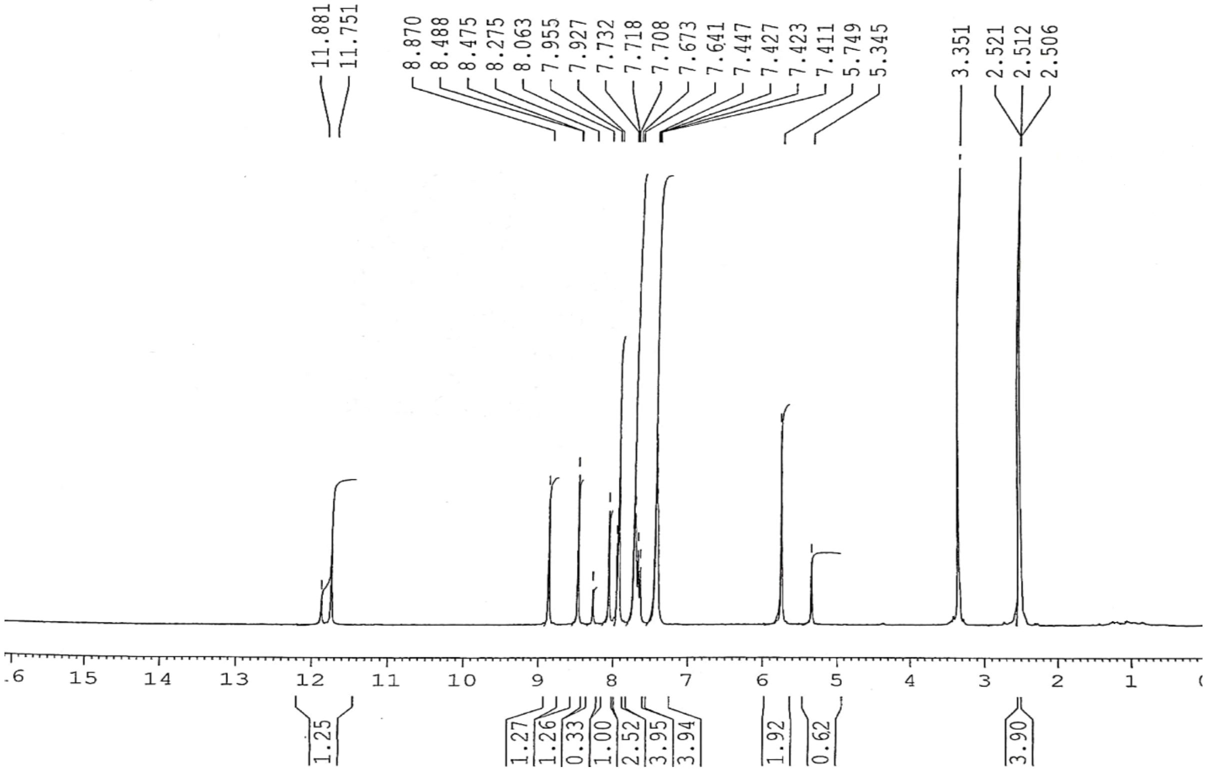

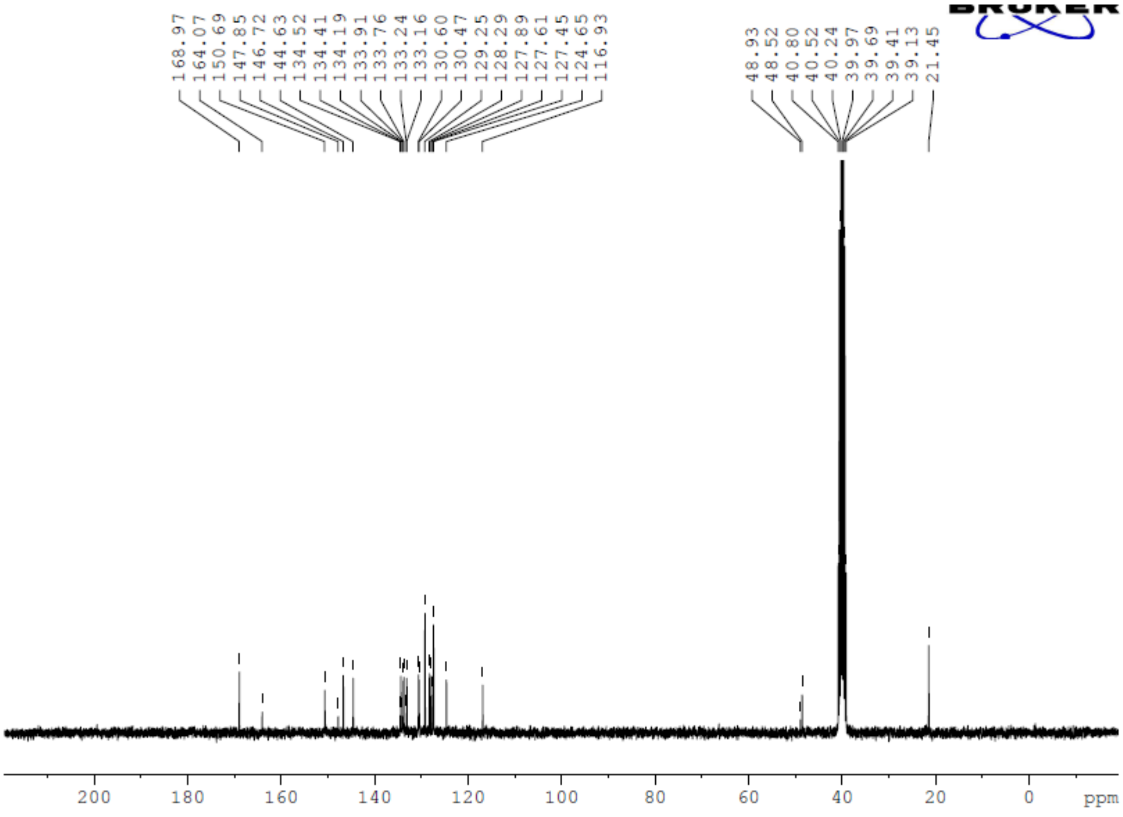

Spectra of 6b:

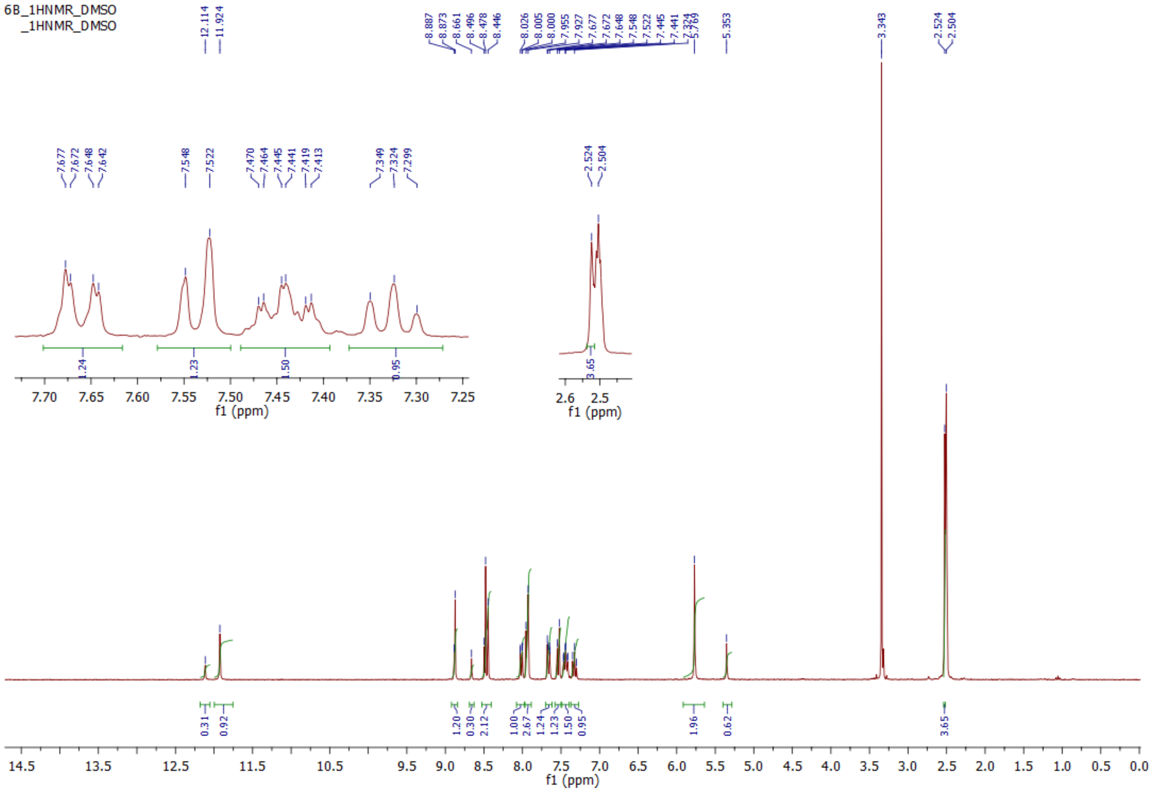

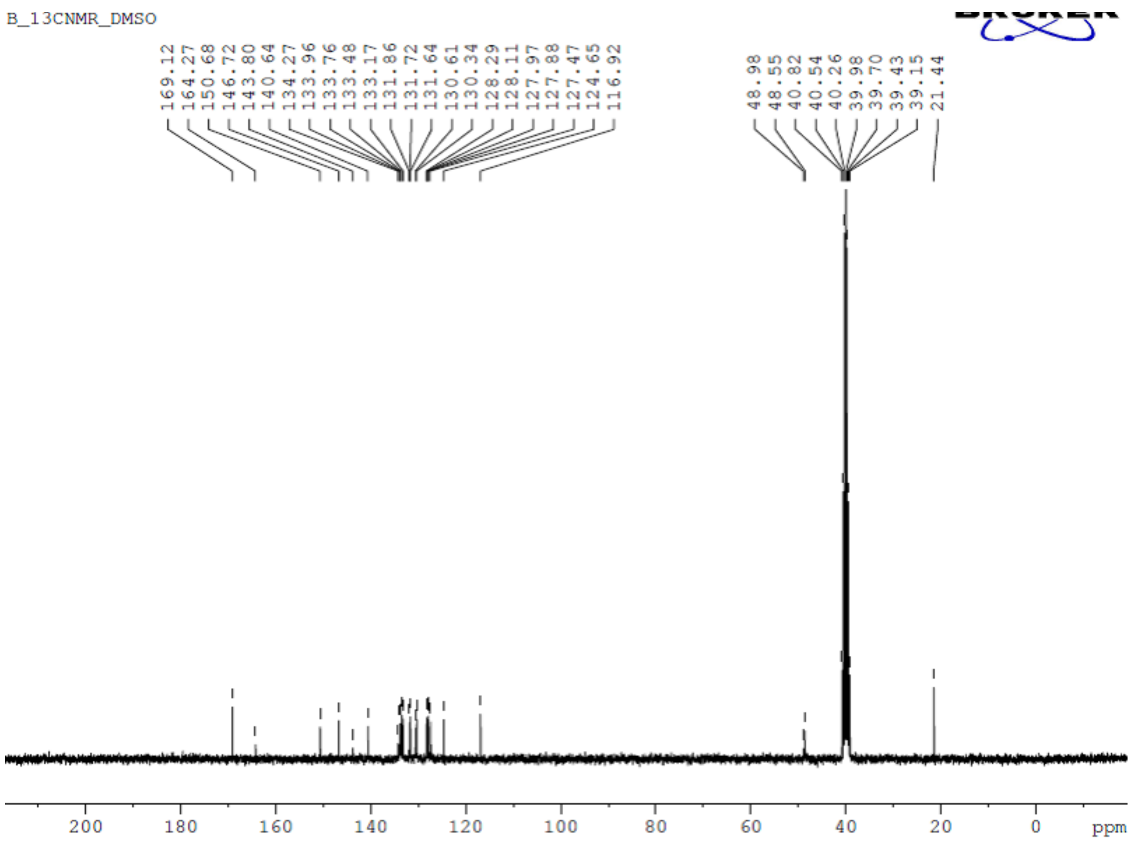

Supplementary Information

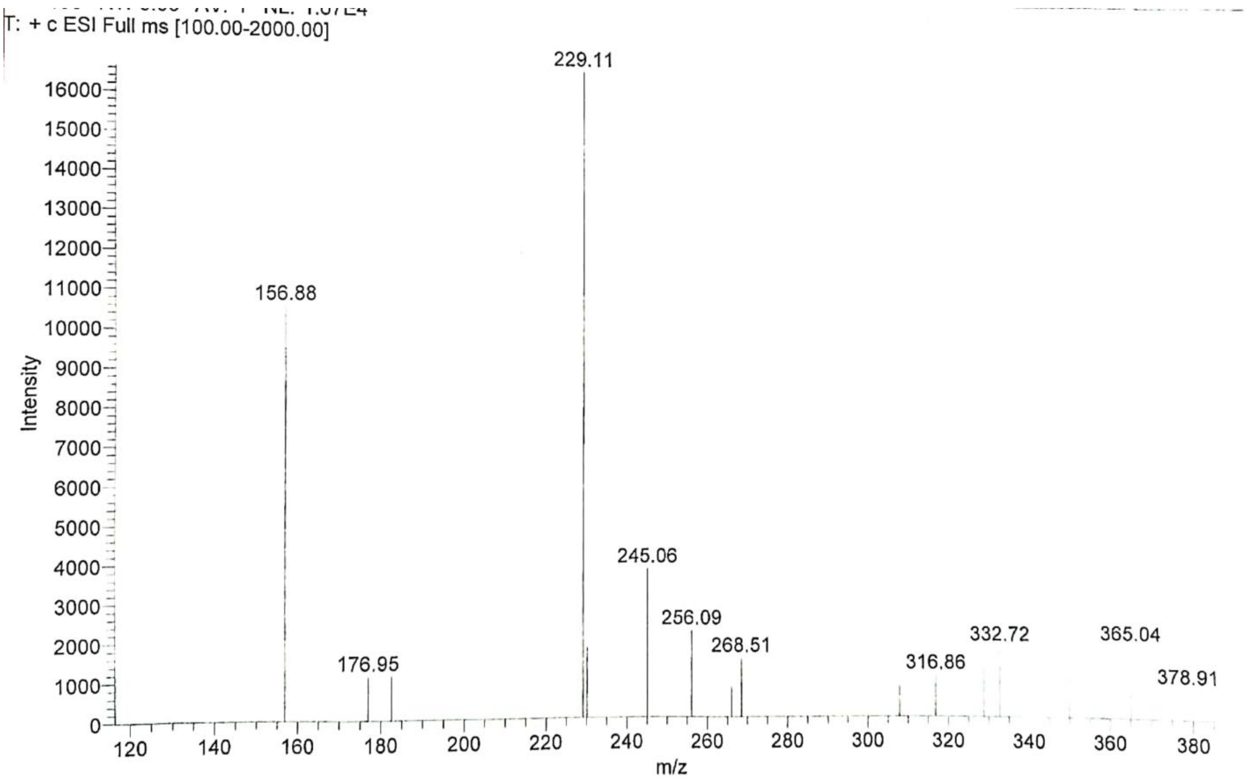

Supplementary Information

Spectra of 6c:

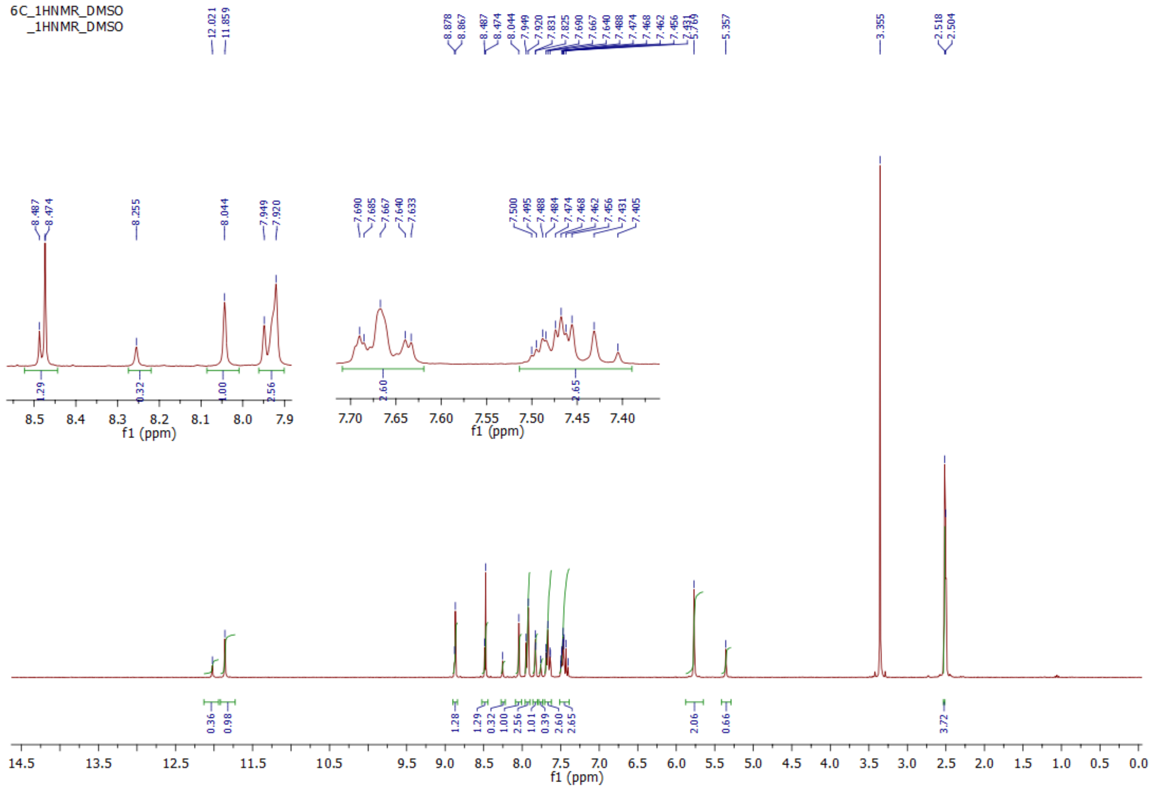

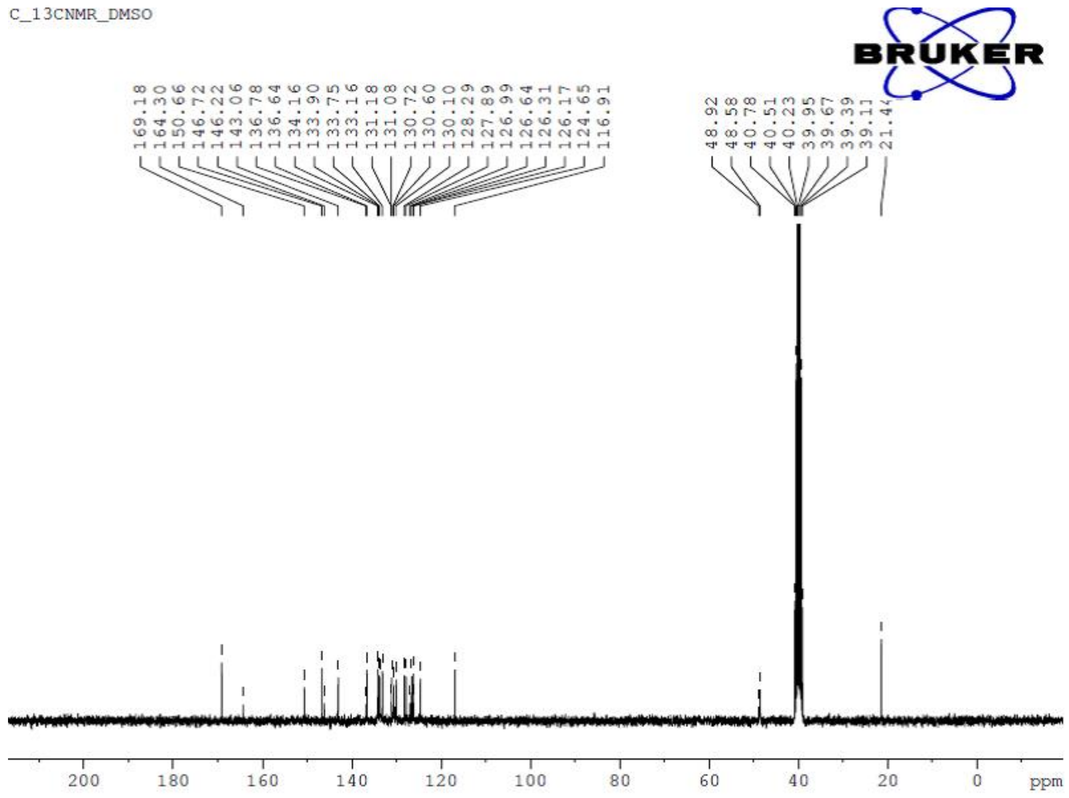

Spectra of 6d:

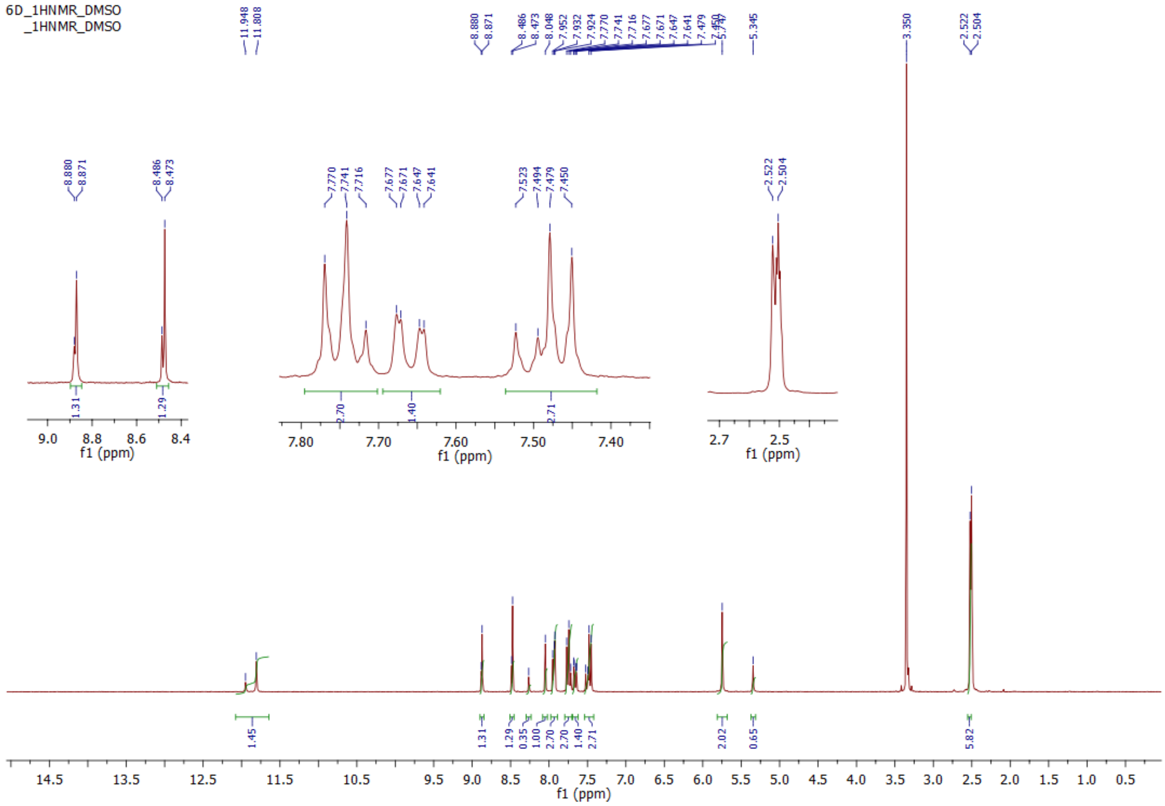

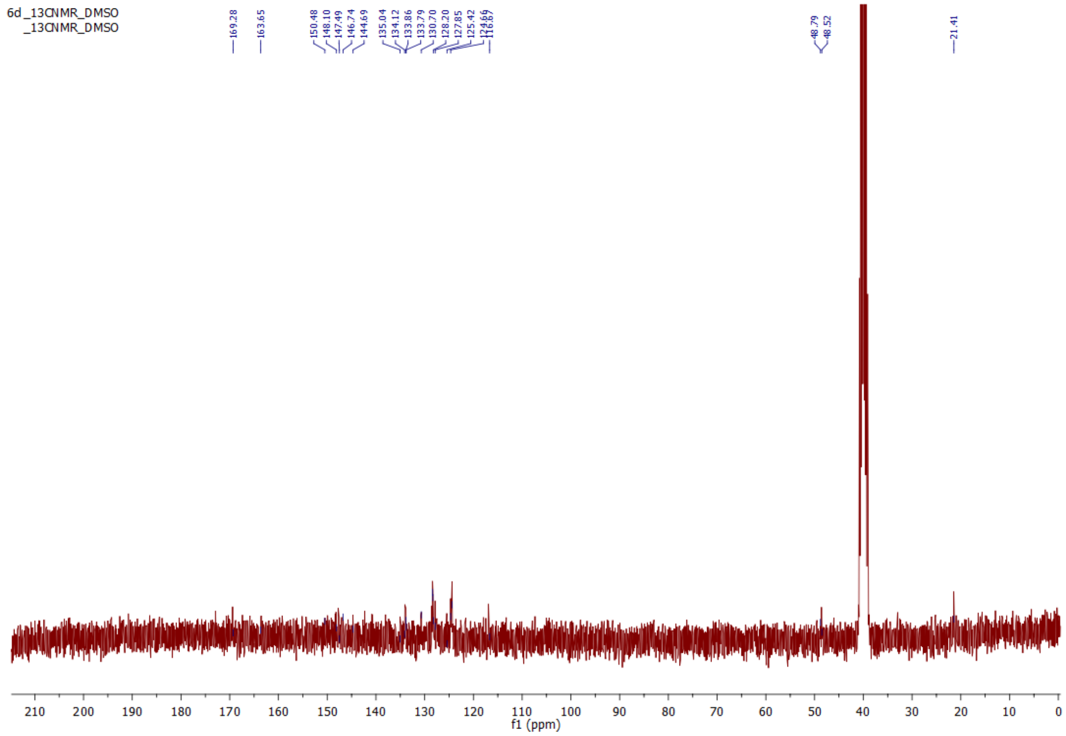

Spectra of 6e:

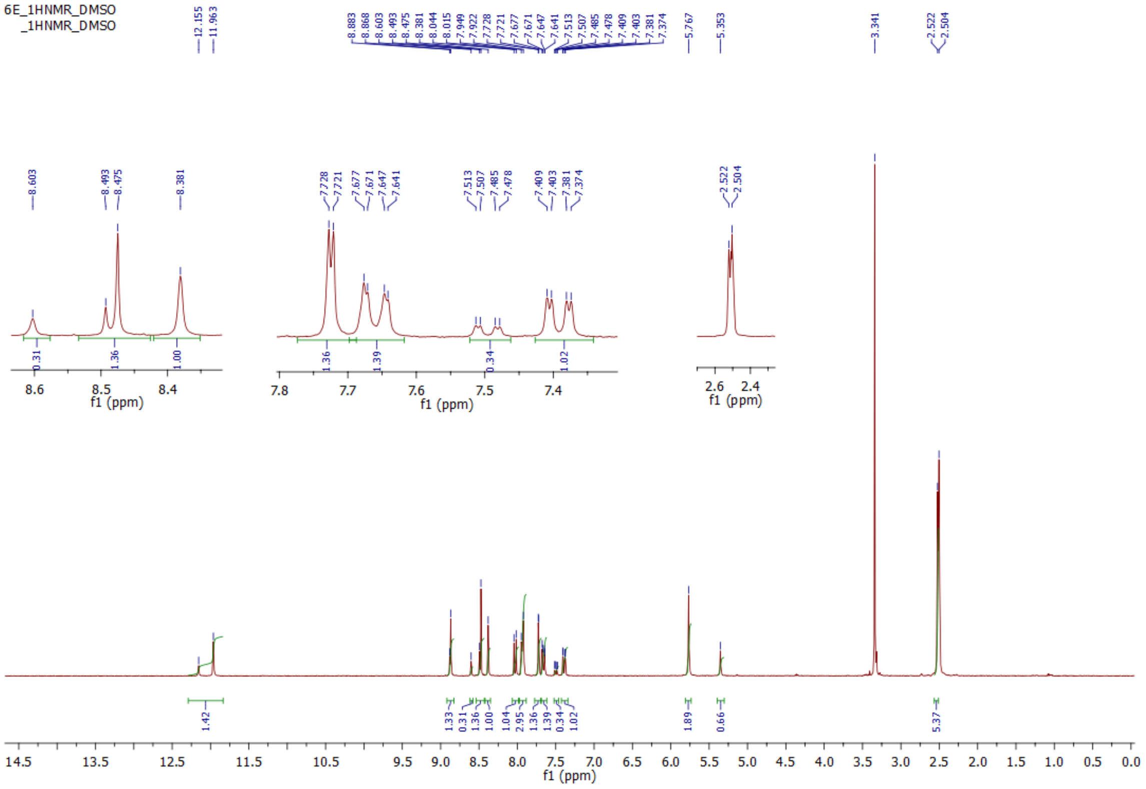

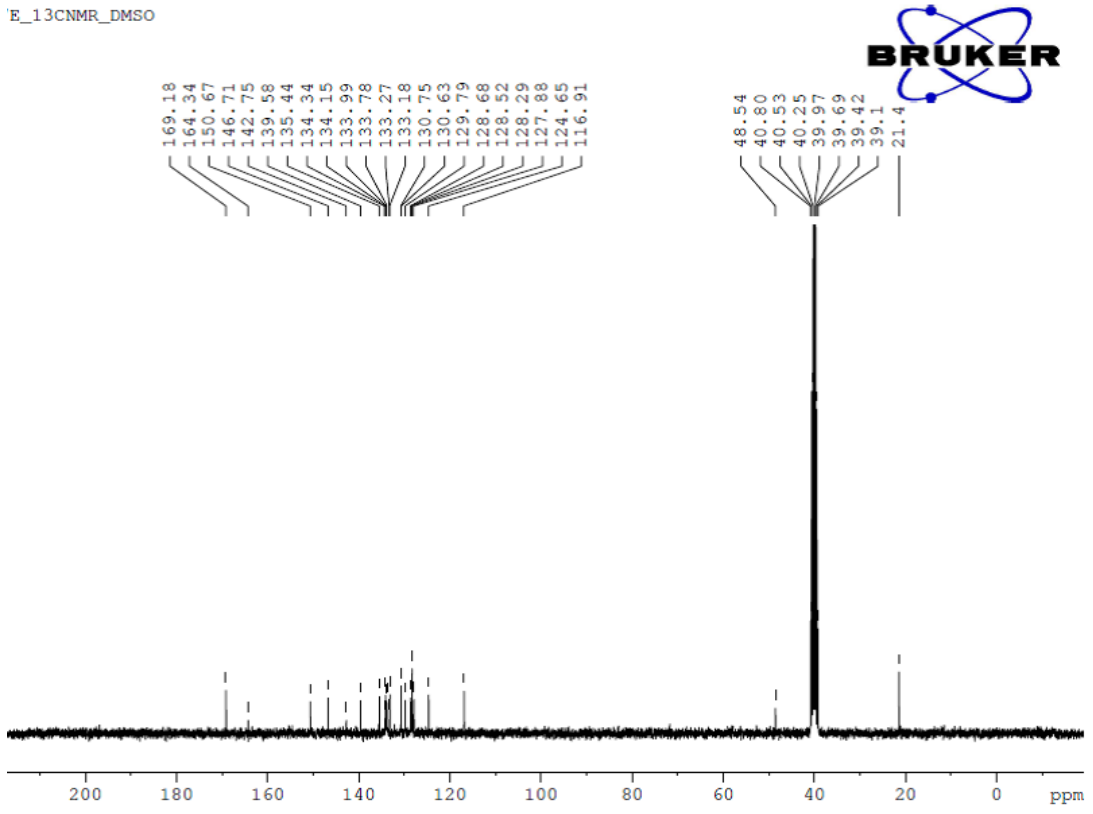

Supplementary Information

Spectra of 6f:

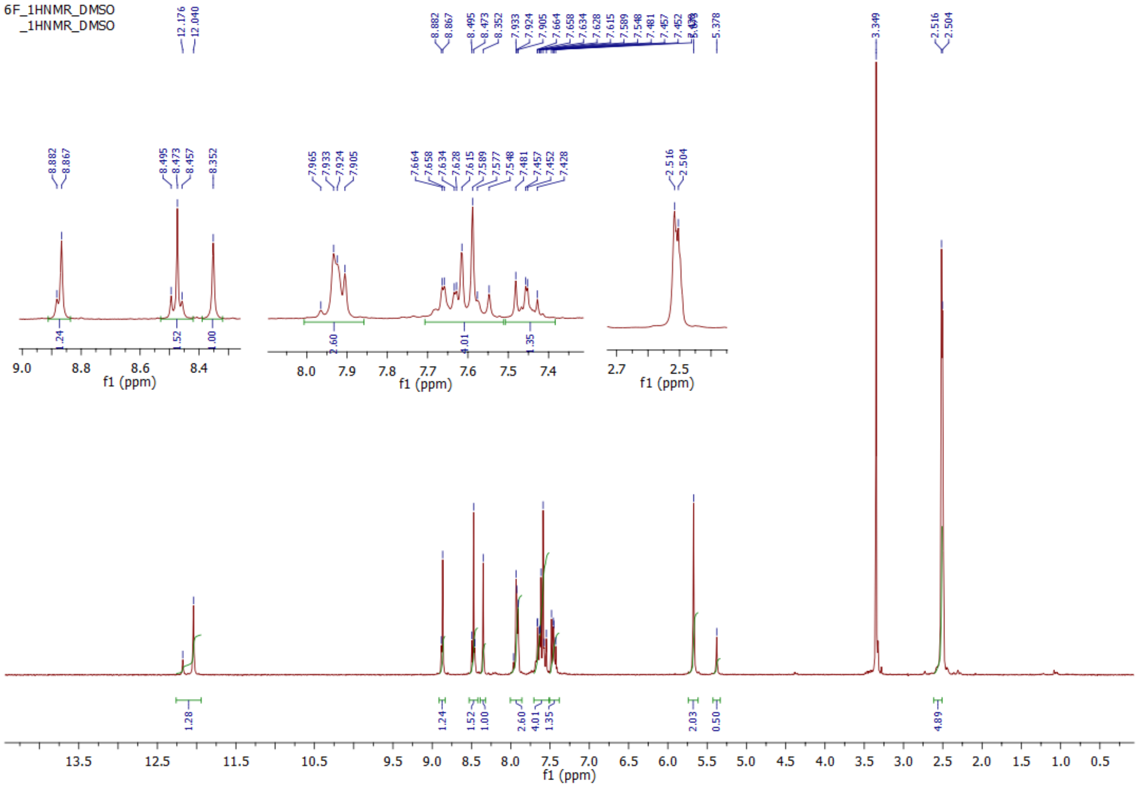

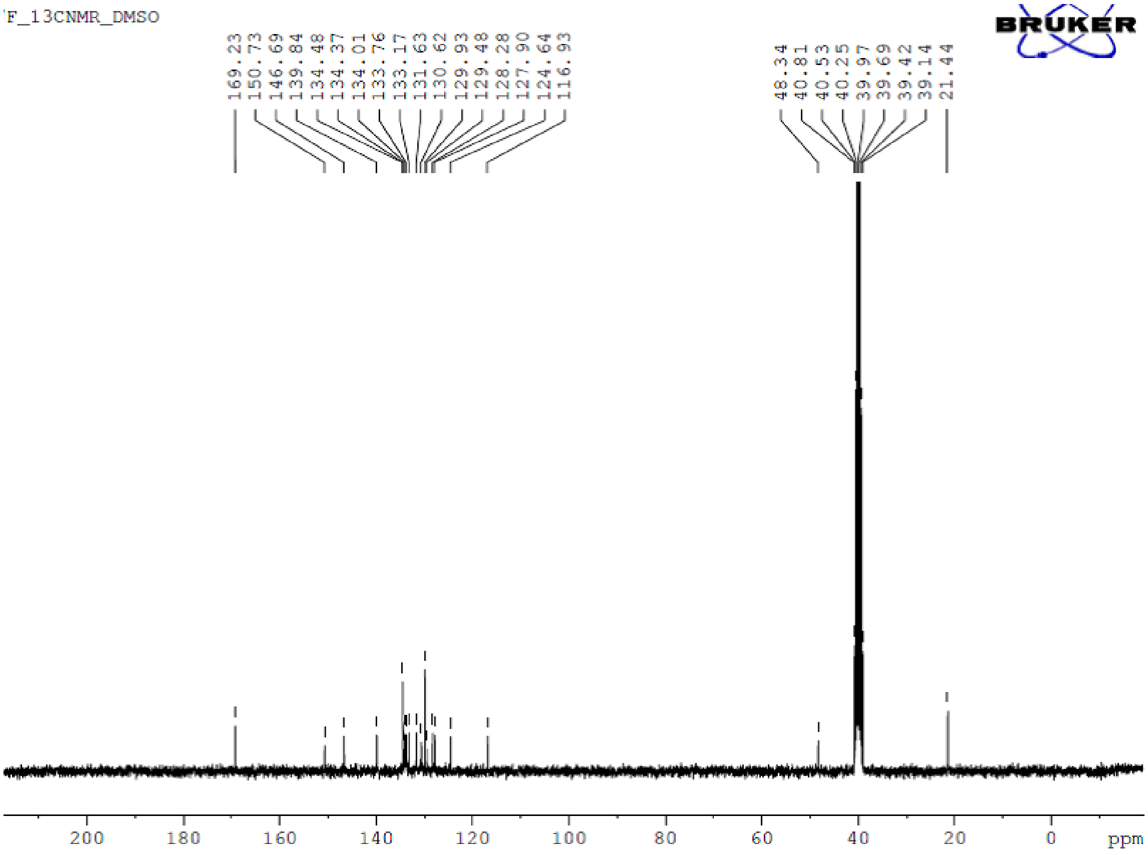

Supplementary Information

Spectra of 6g:

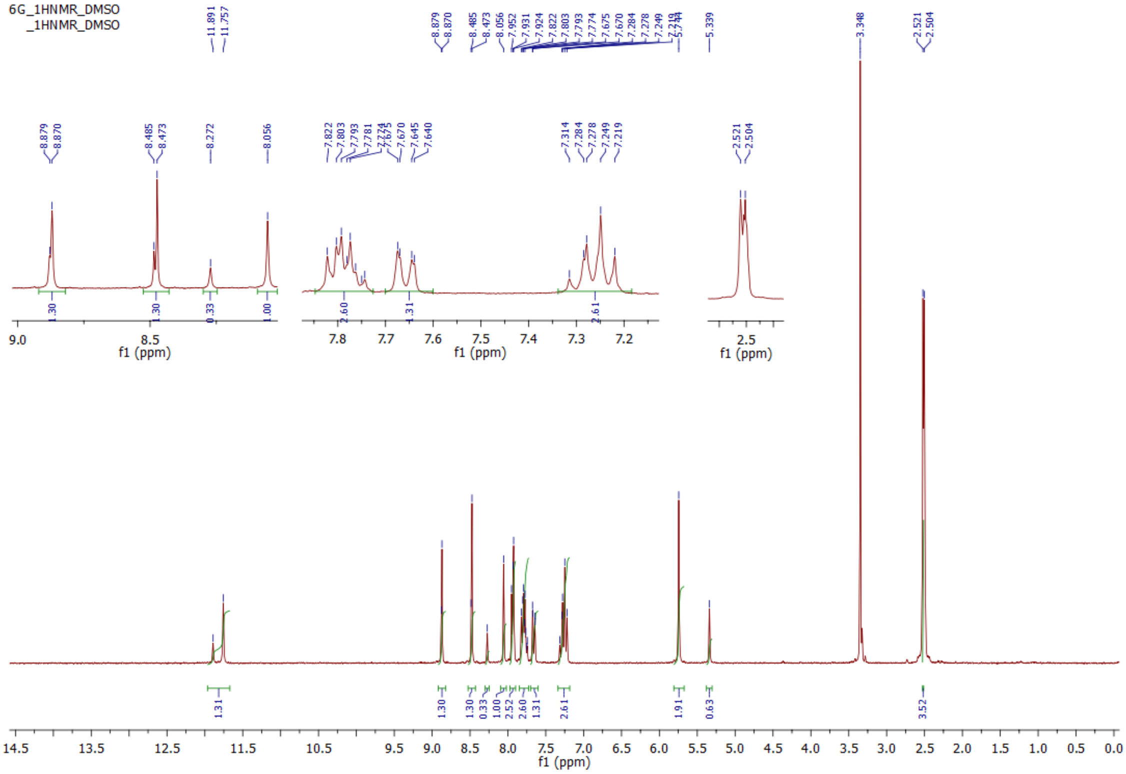

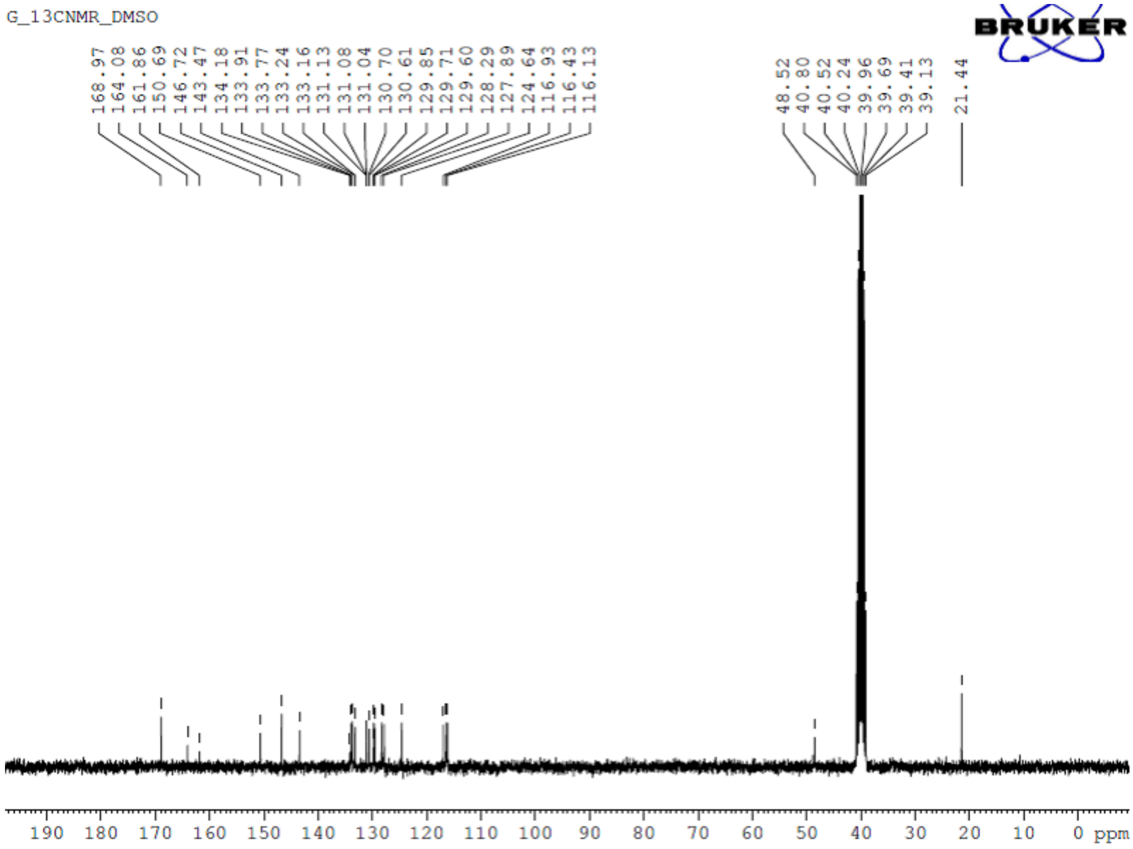

Supplementary Information

Spectra of 6h:

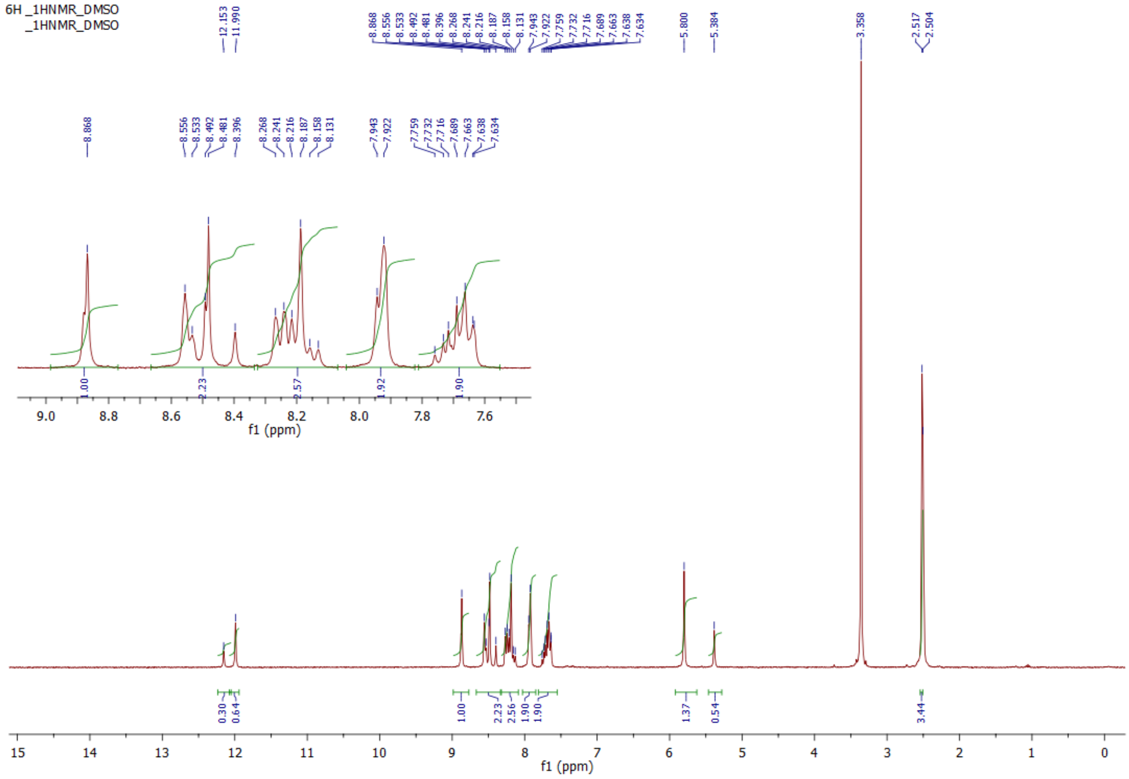

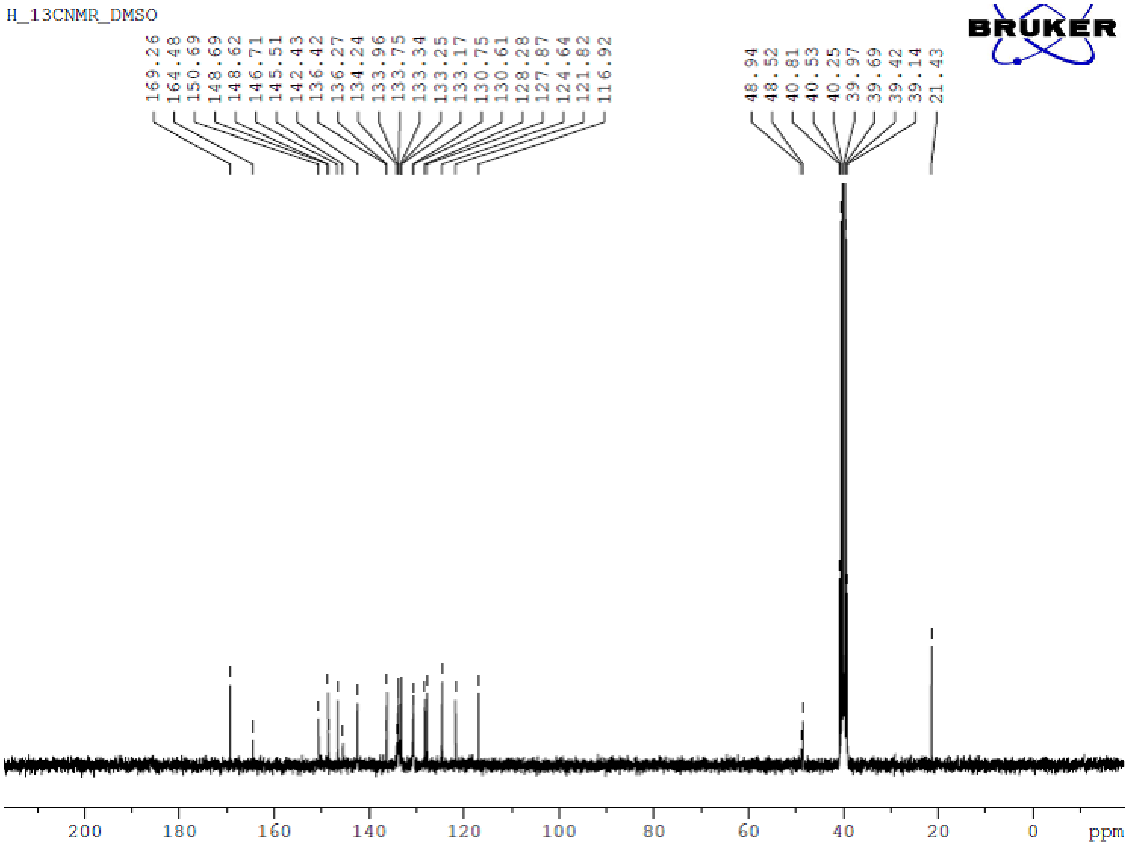

Spectra of 6i:

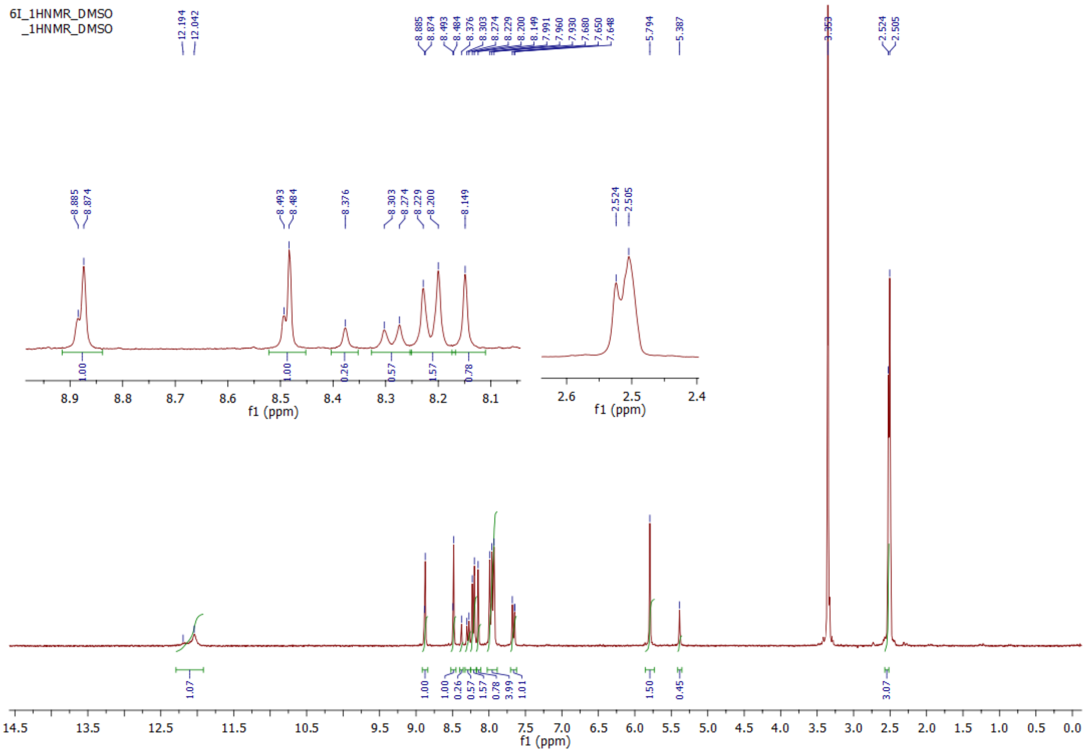

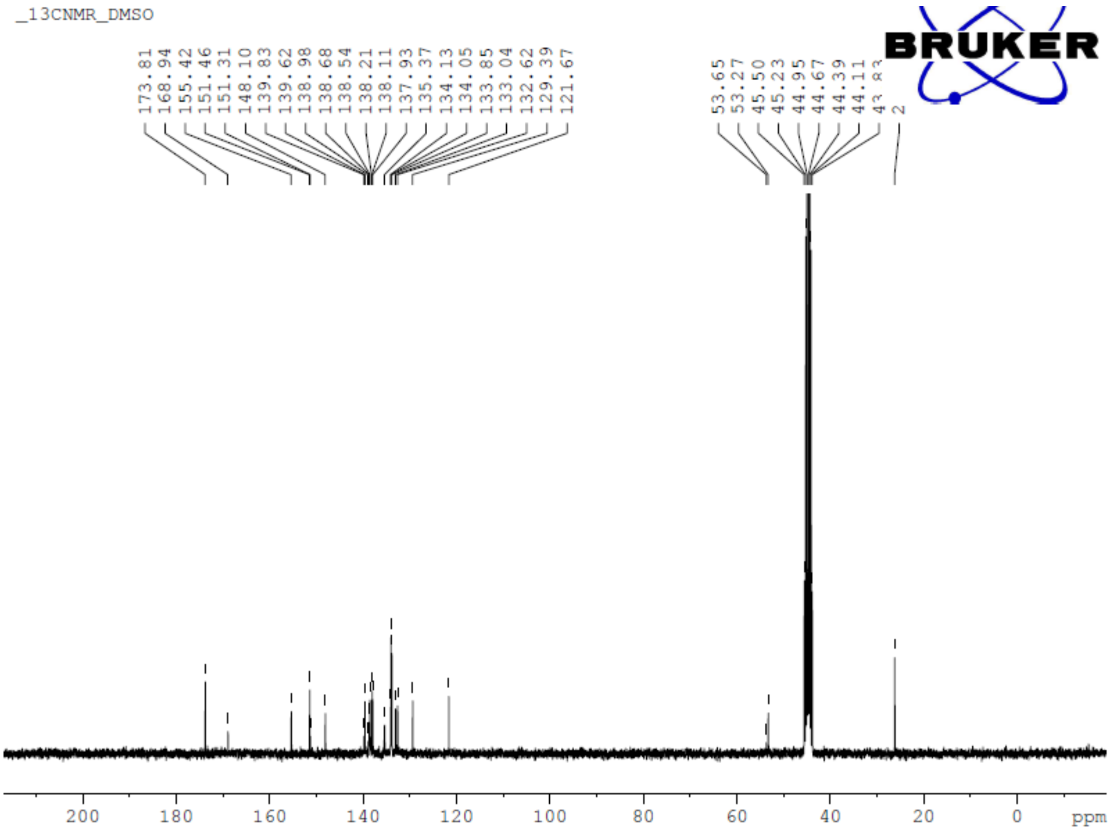

Supplementary Information

Spectra of 6j:

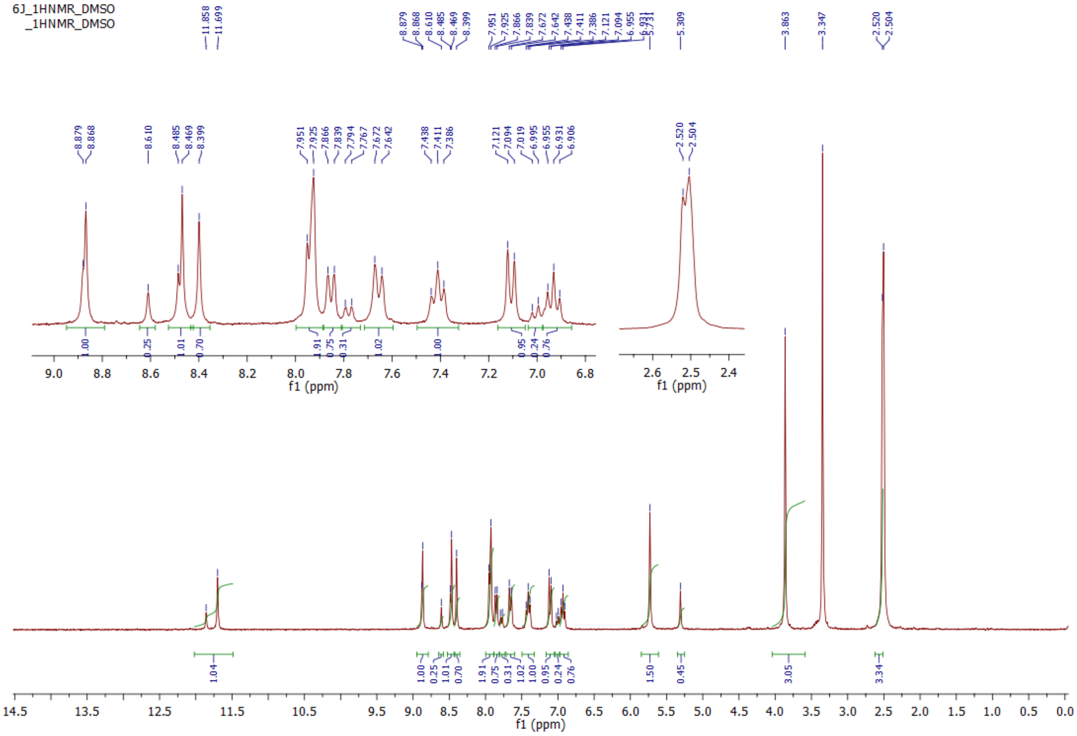

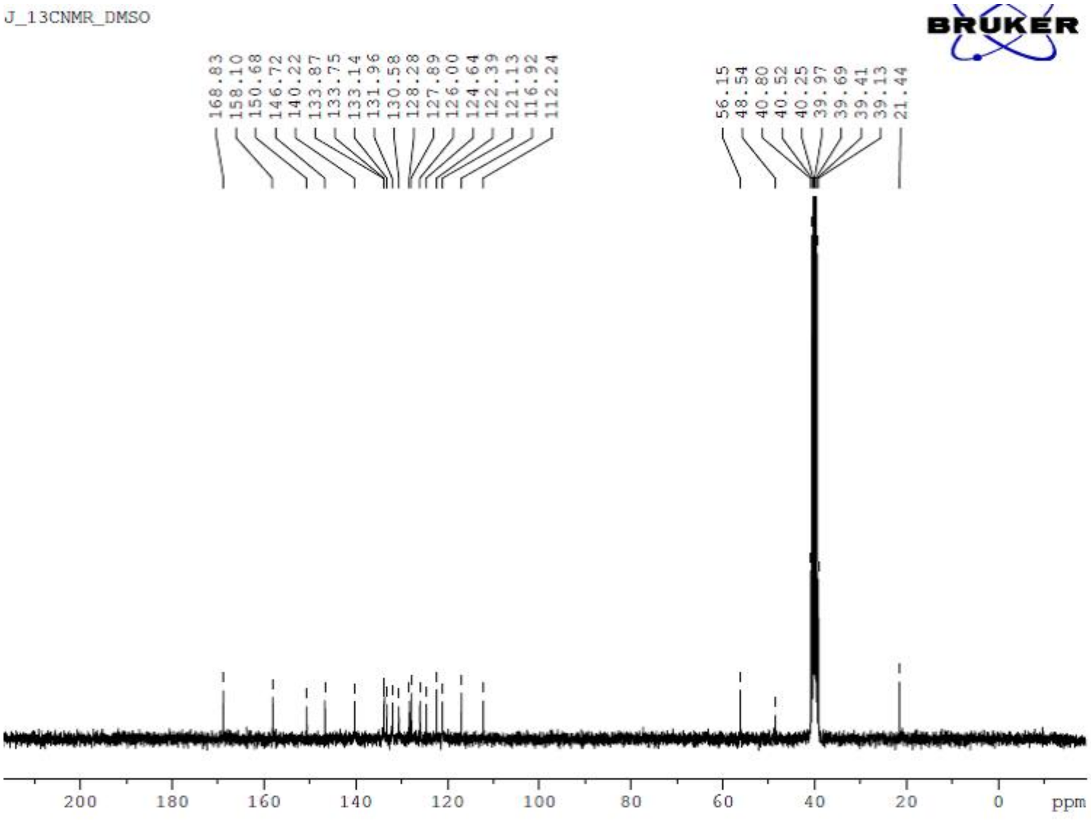

Supplementary Information

Spectra of 6k

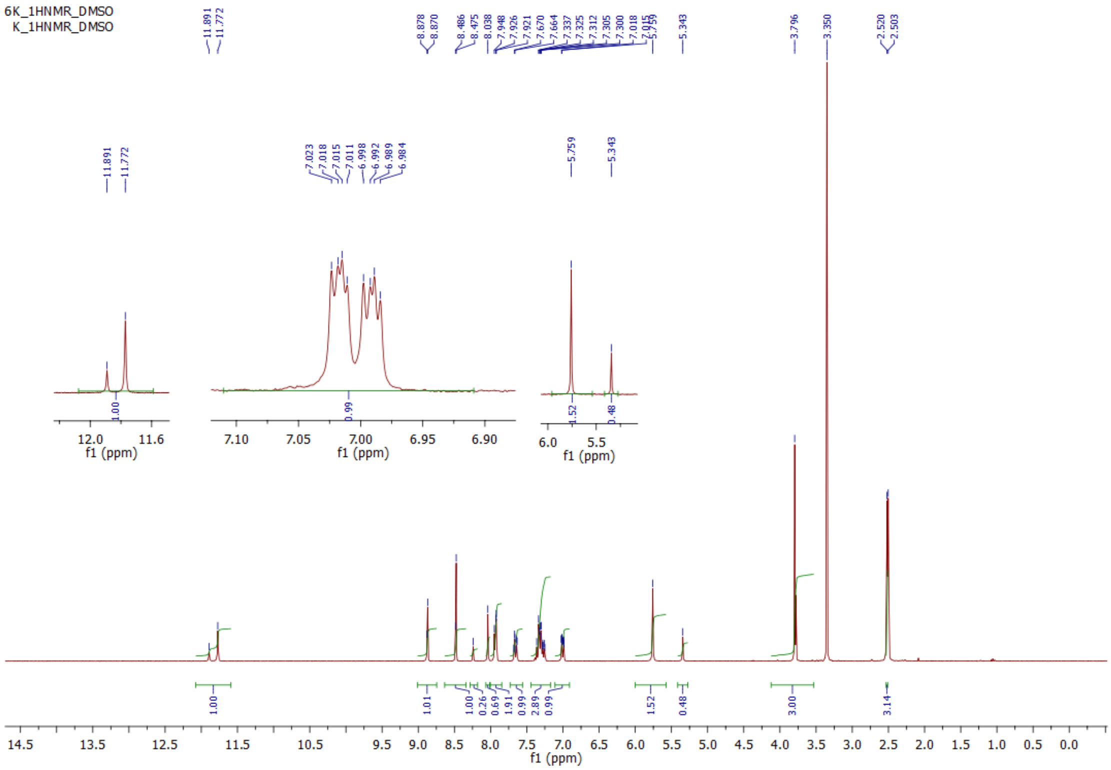

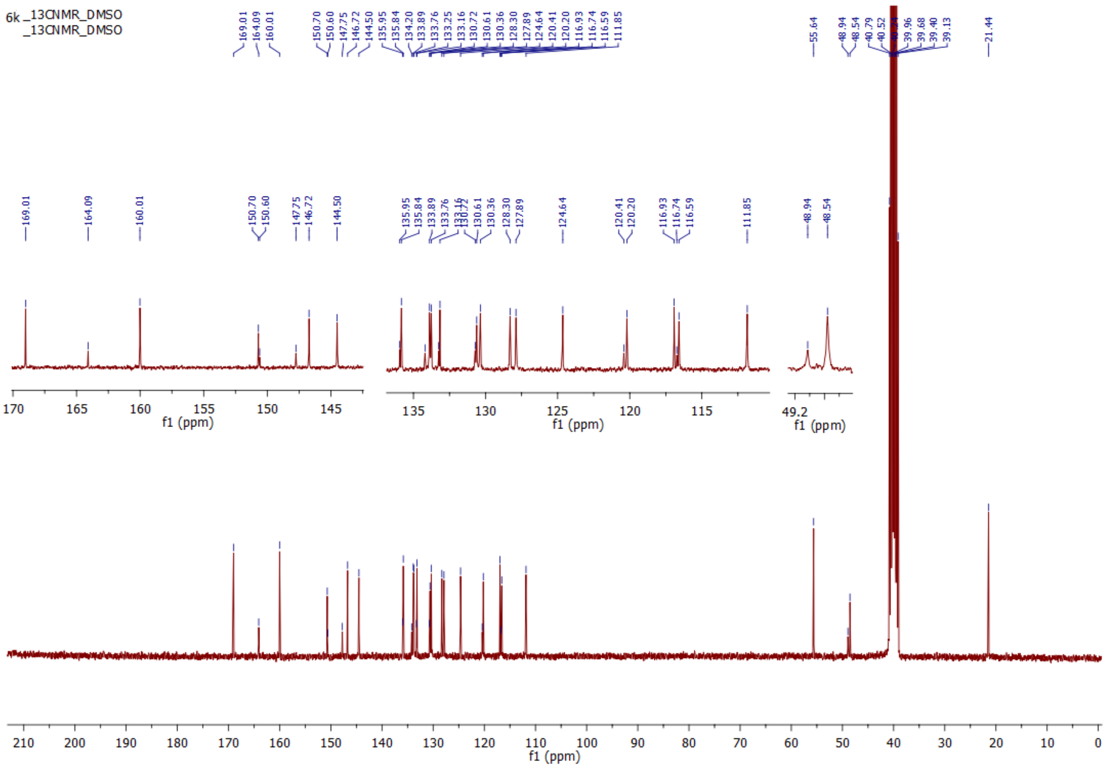

Spectra of 6l

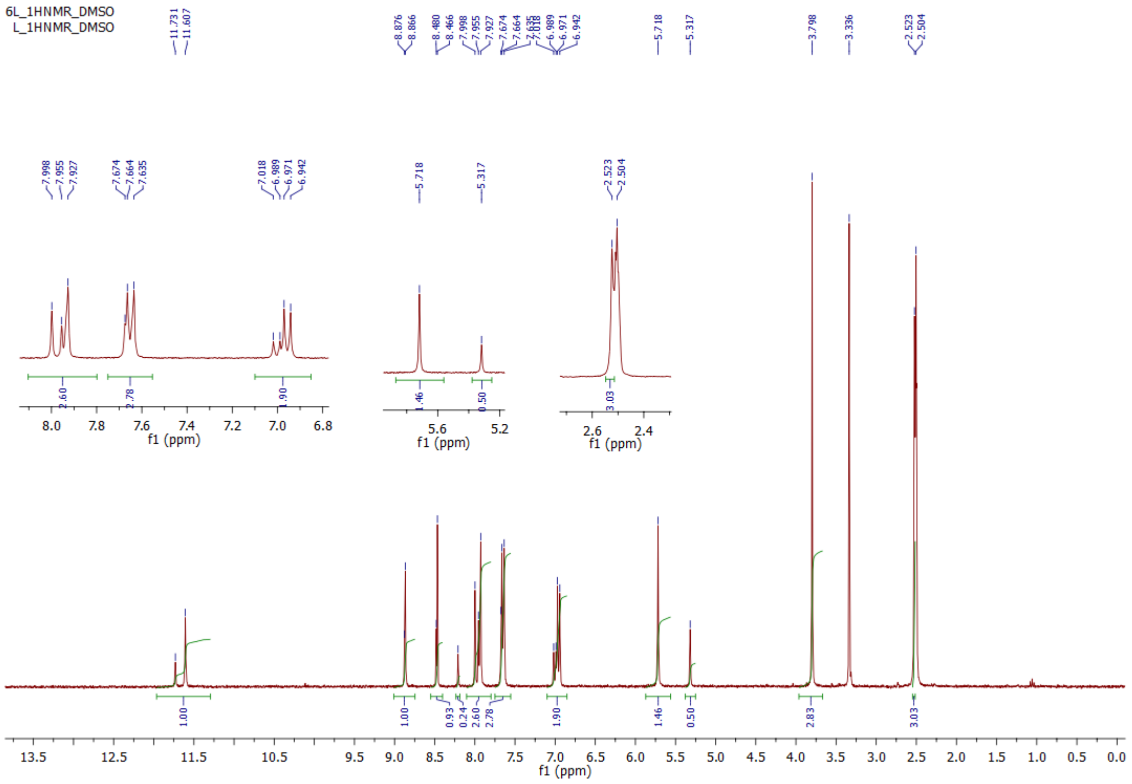

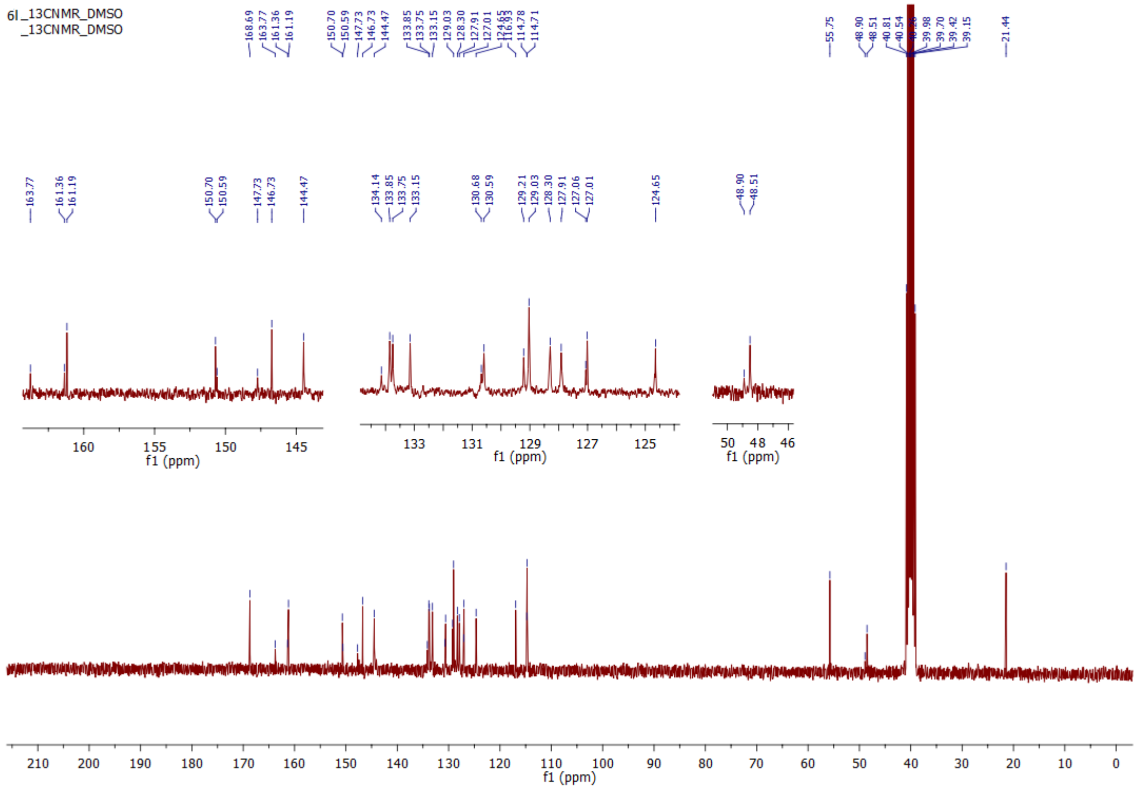

Supplementary Information

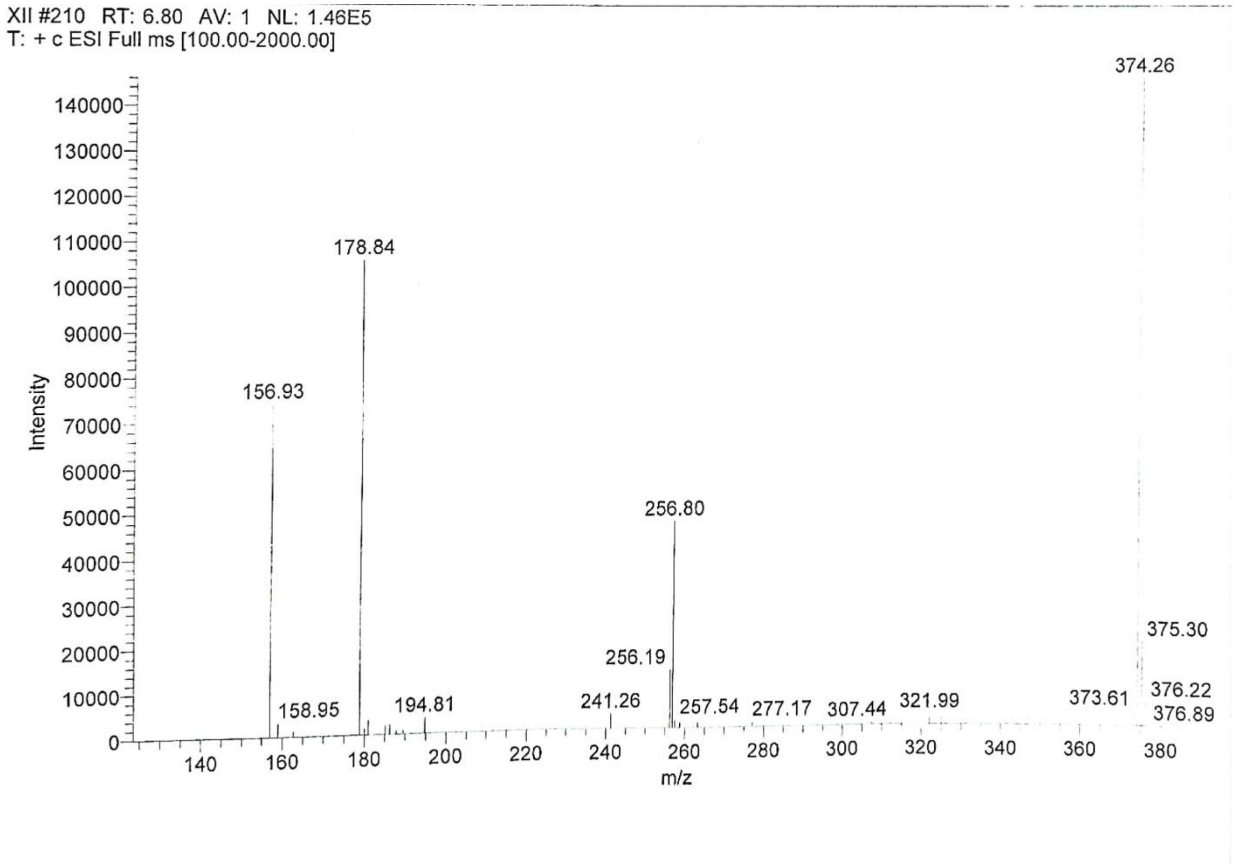

Supplementary Information

Spectra of 6m

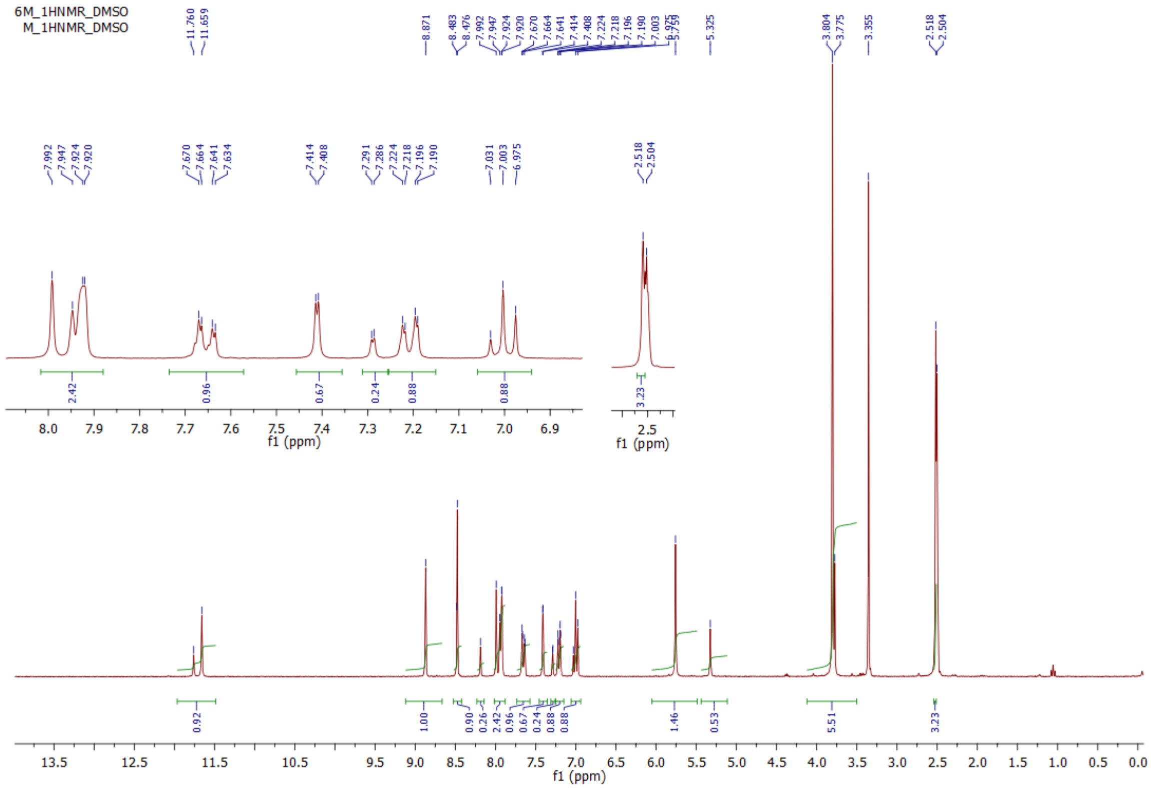

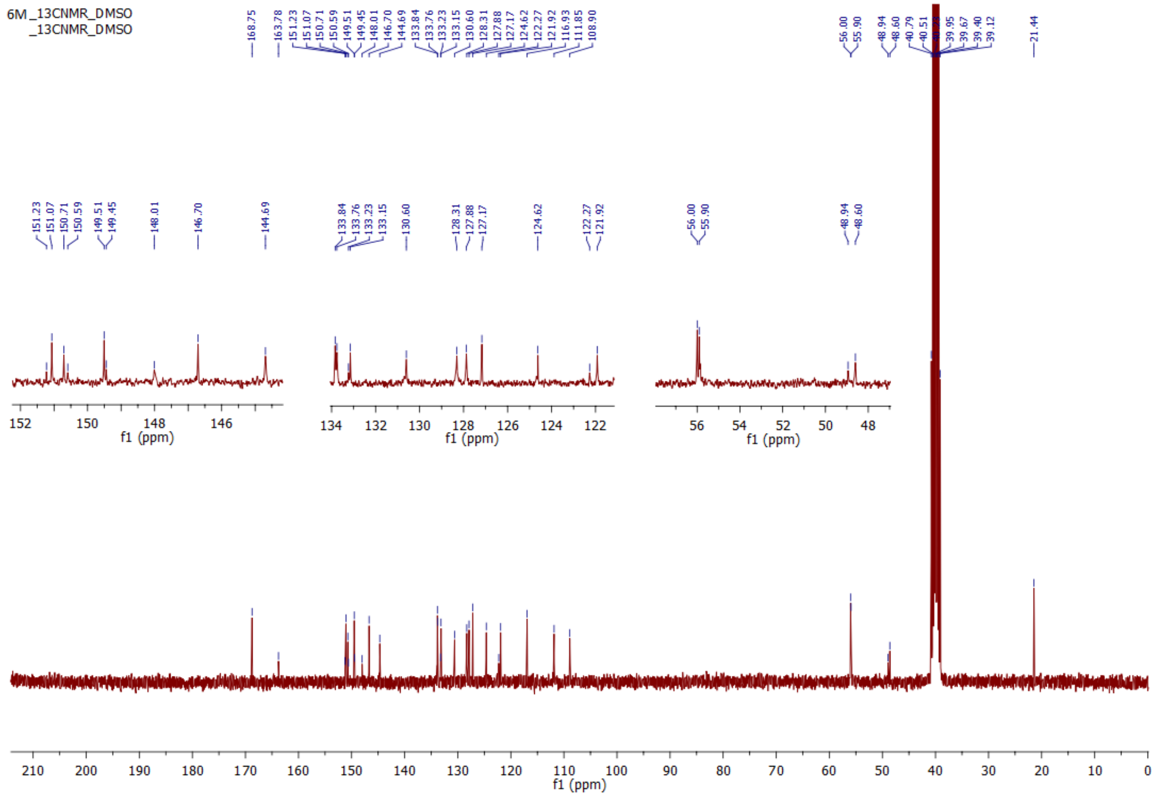

Supplementary Information

Spectra of 6n

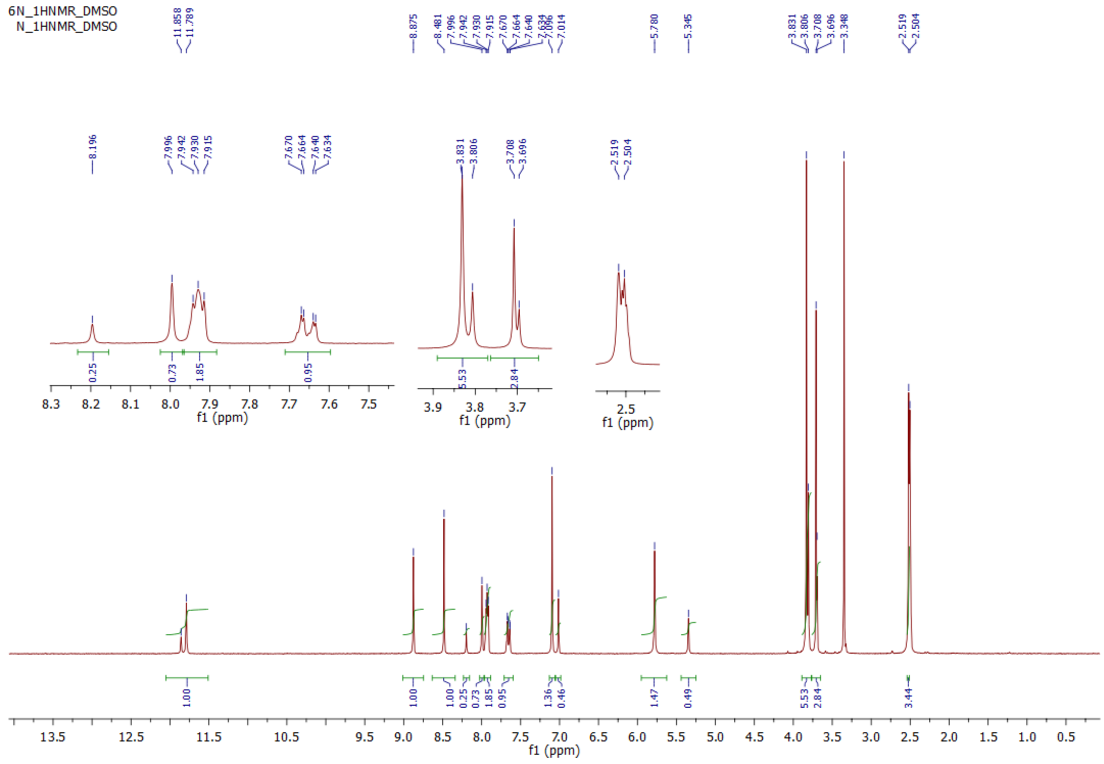

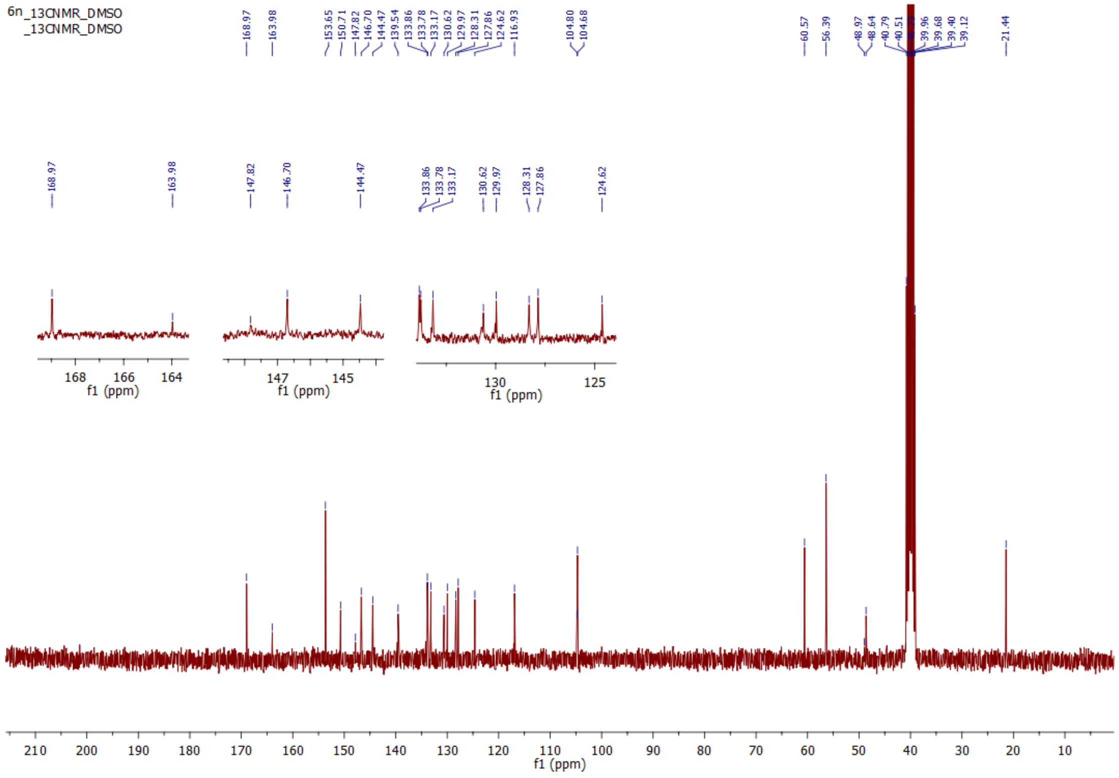

Supplementary Information

Spectra of 6o

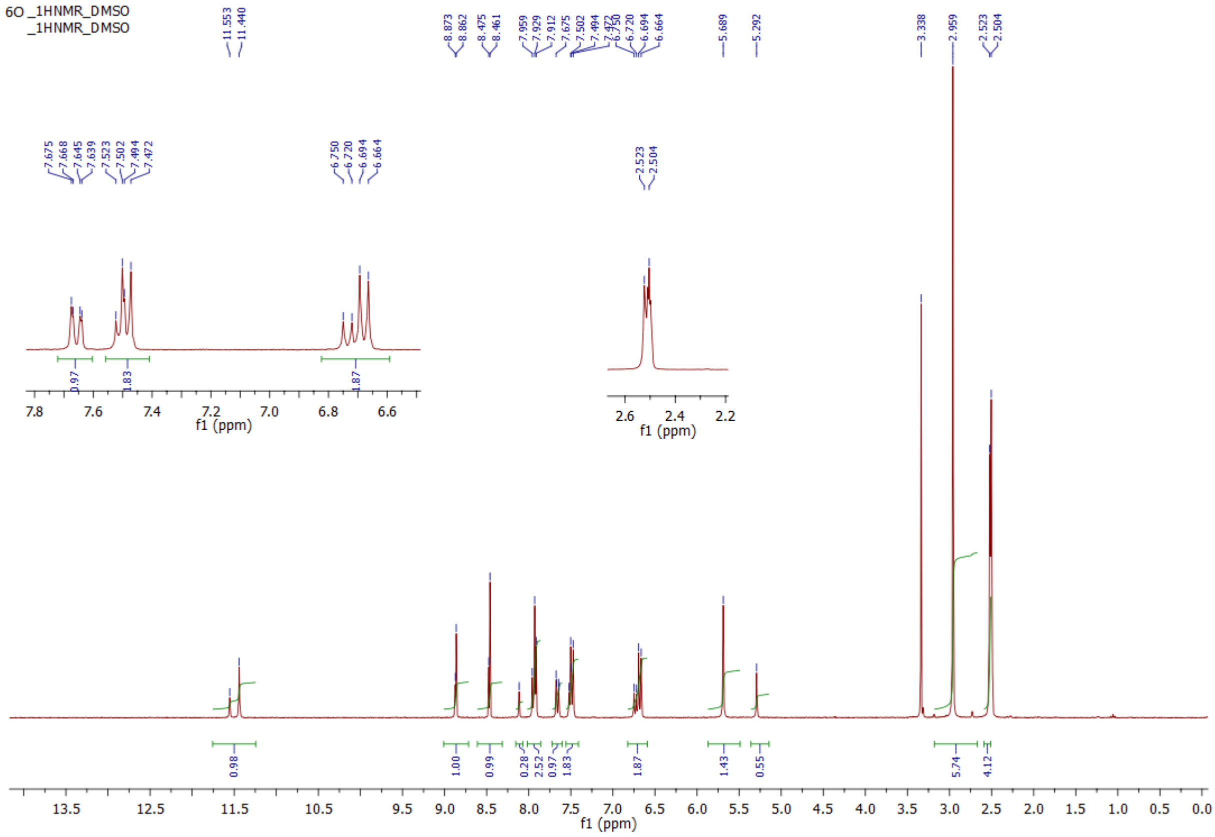

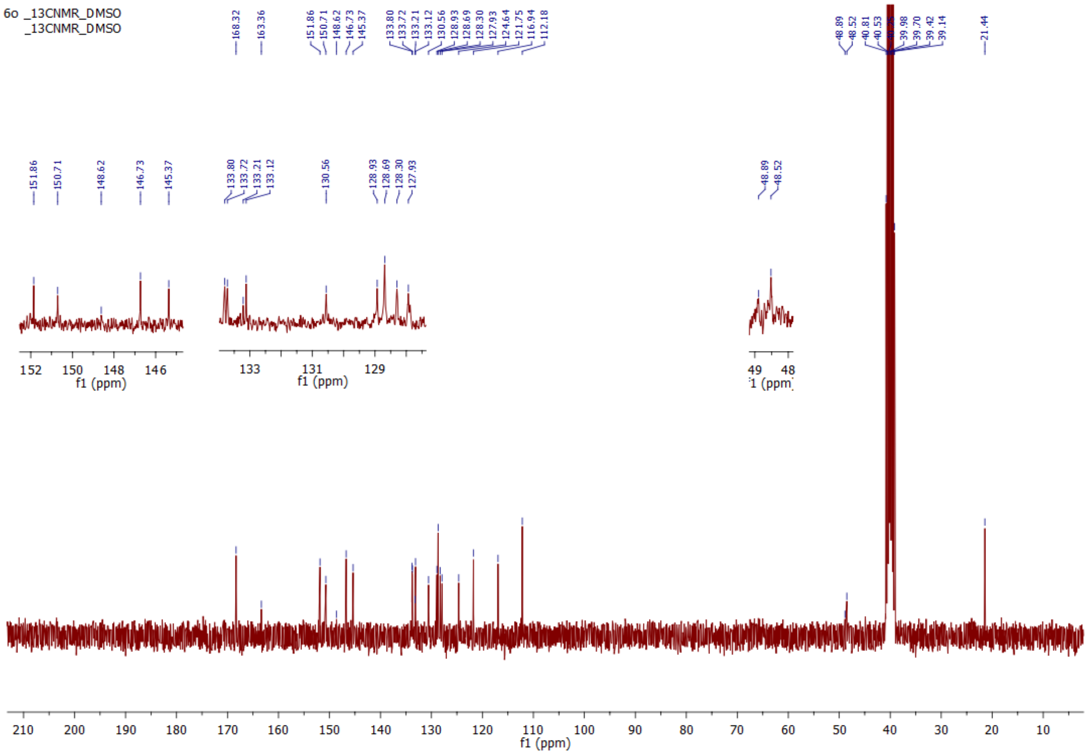

Supplementary Information

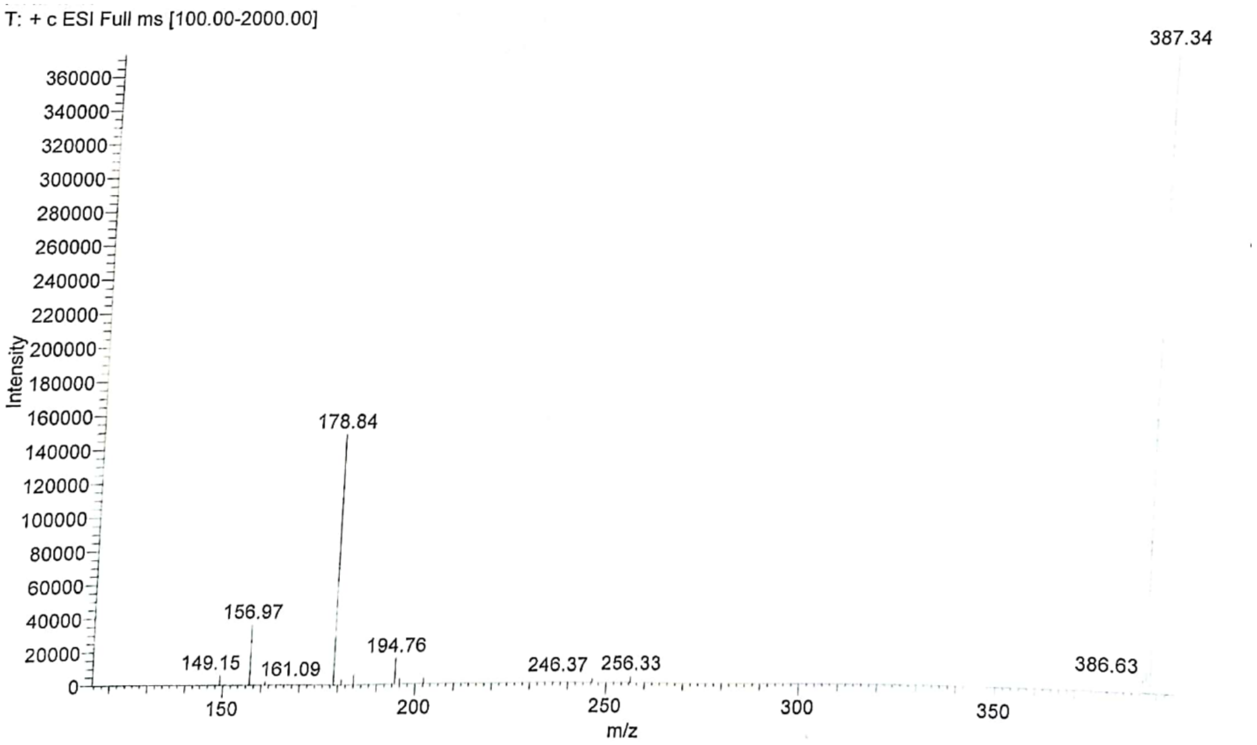

Supplementary Information

Spectra of 6p

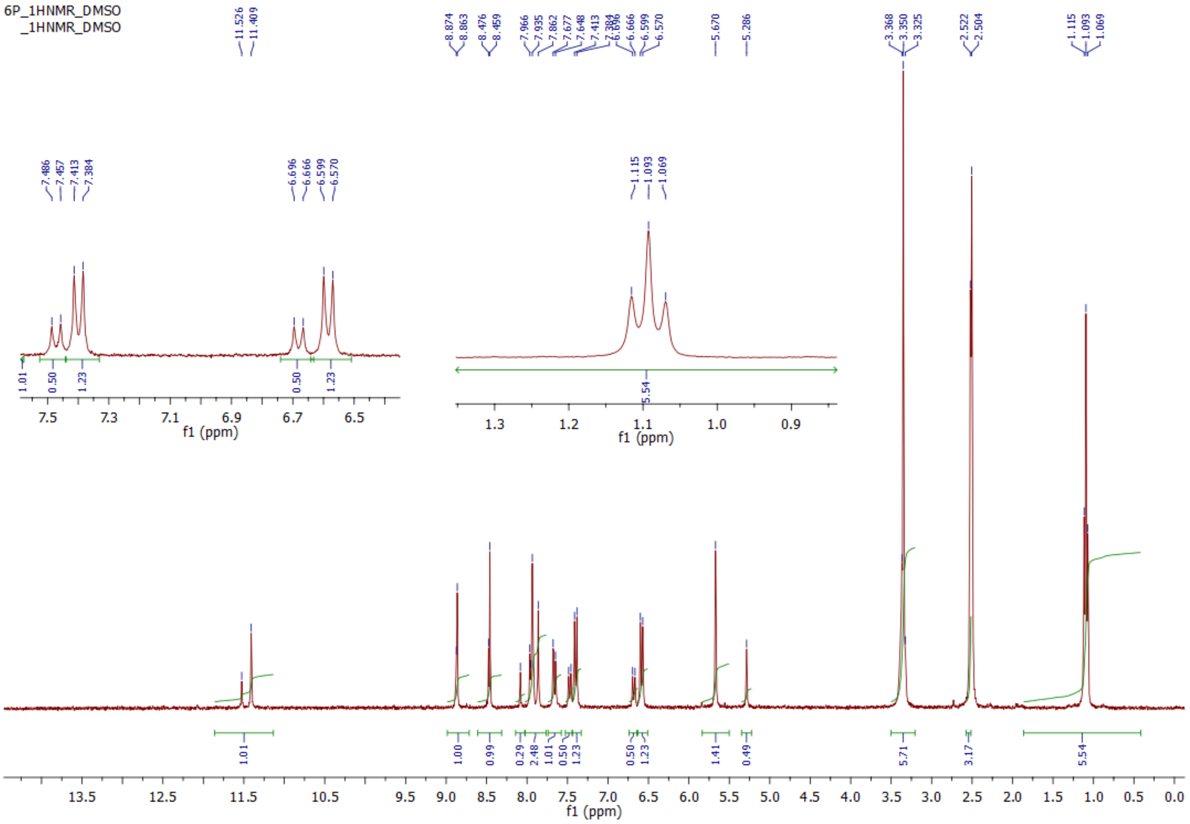

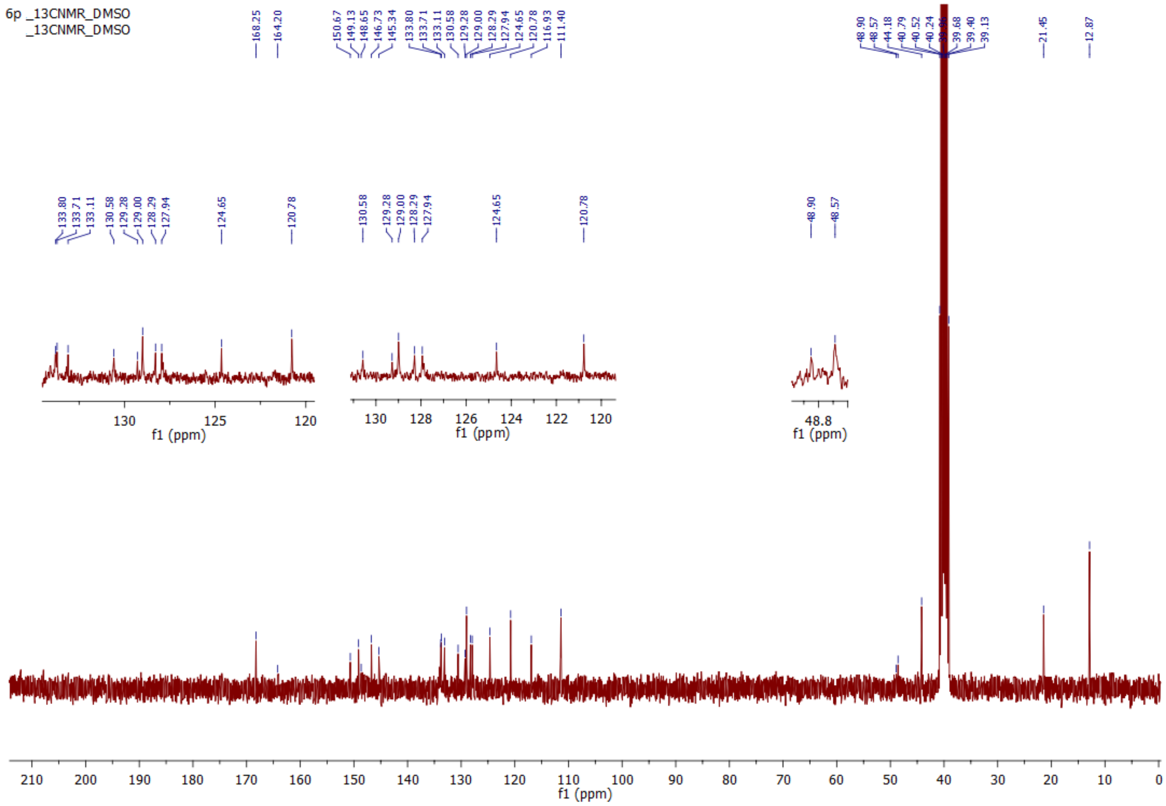

Spectra of 6q

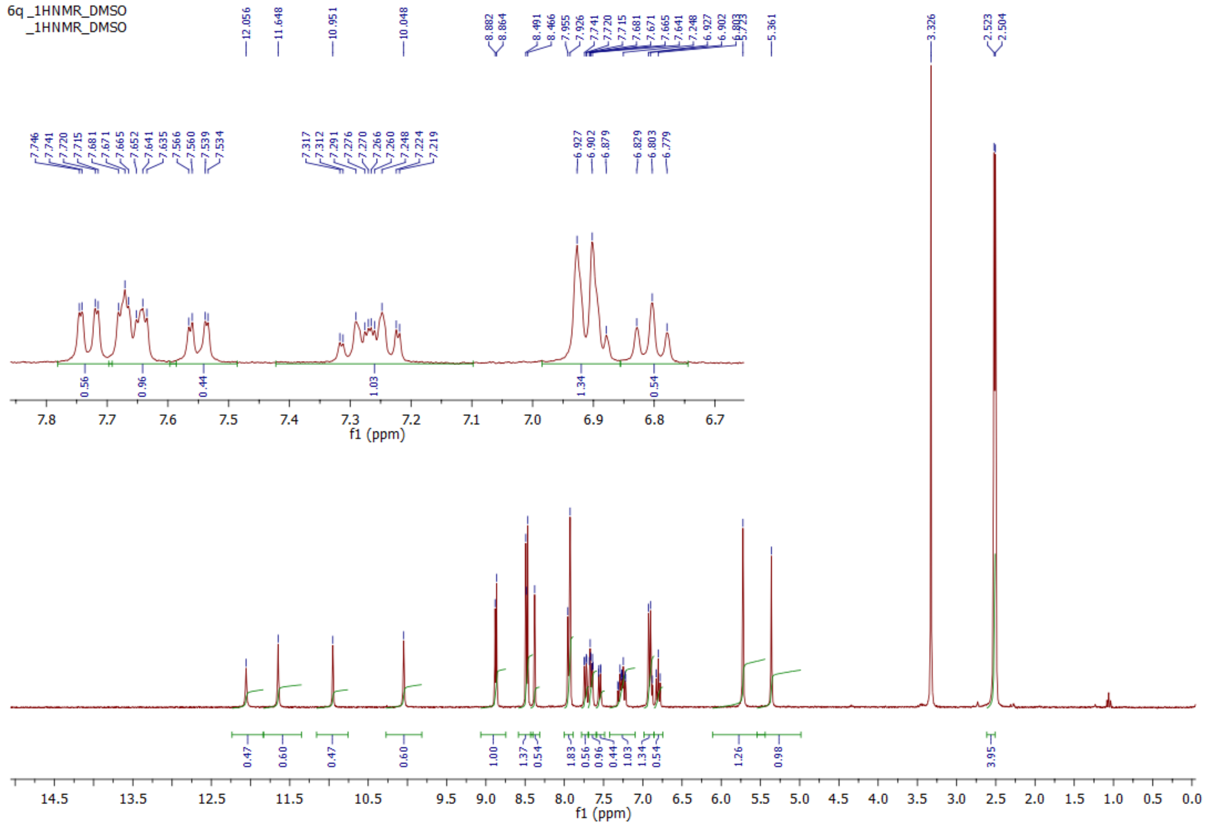

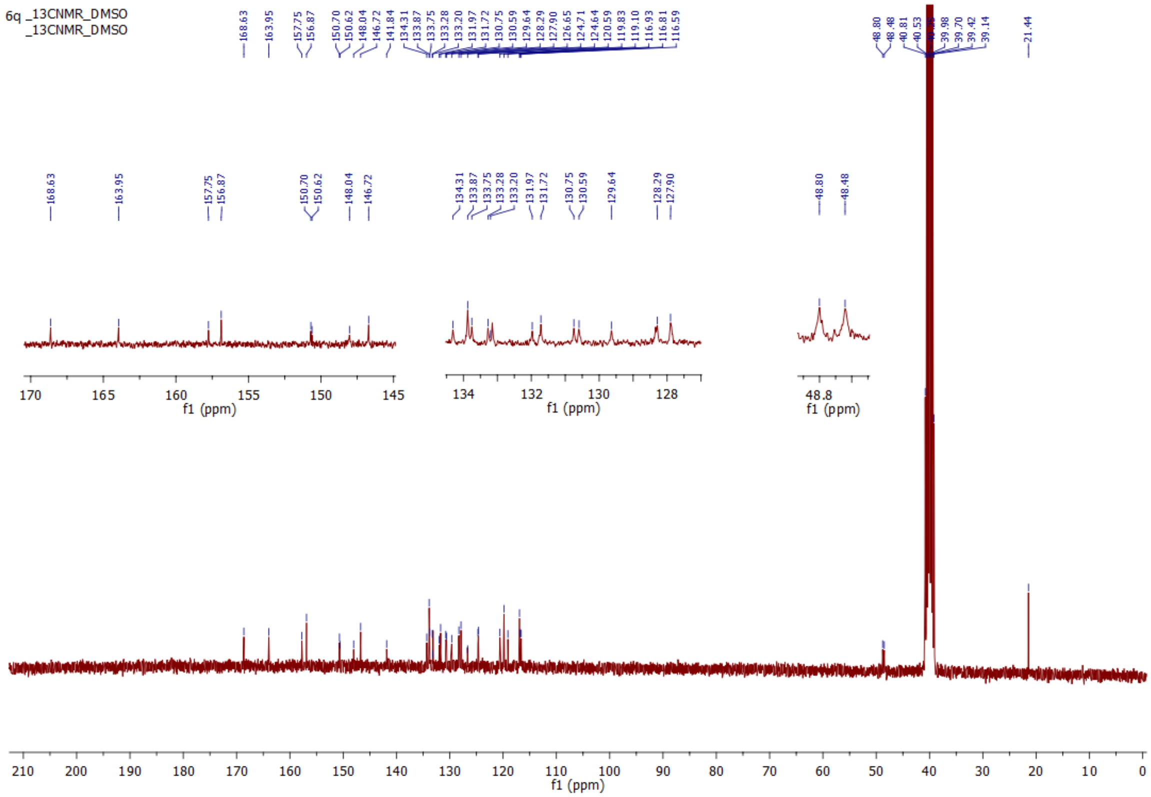

Spectra of 6r

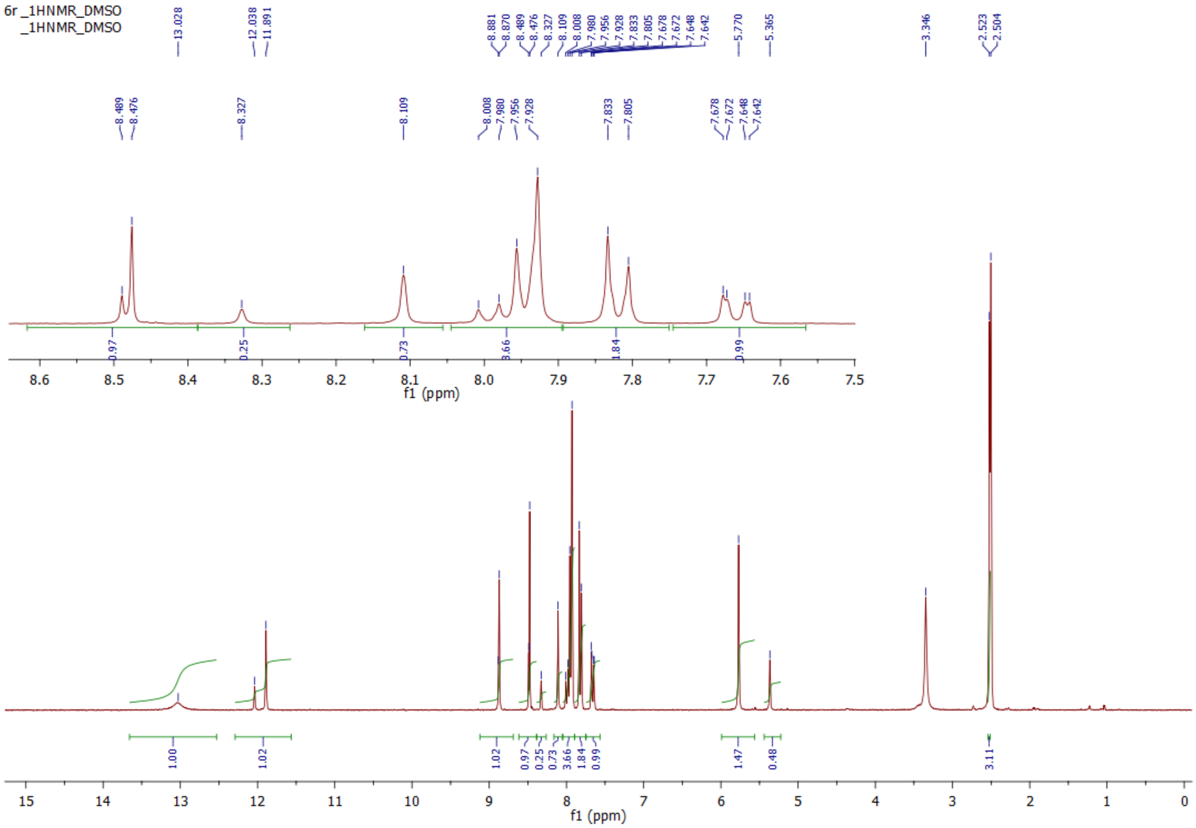

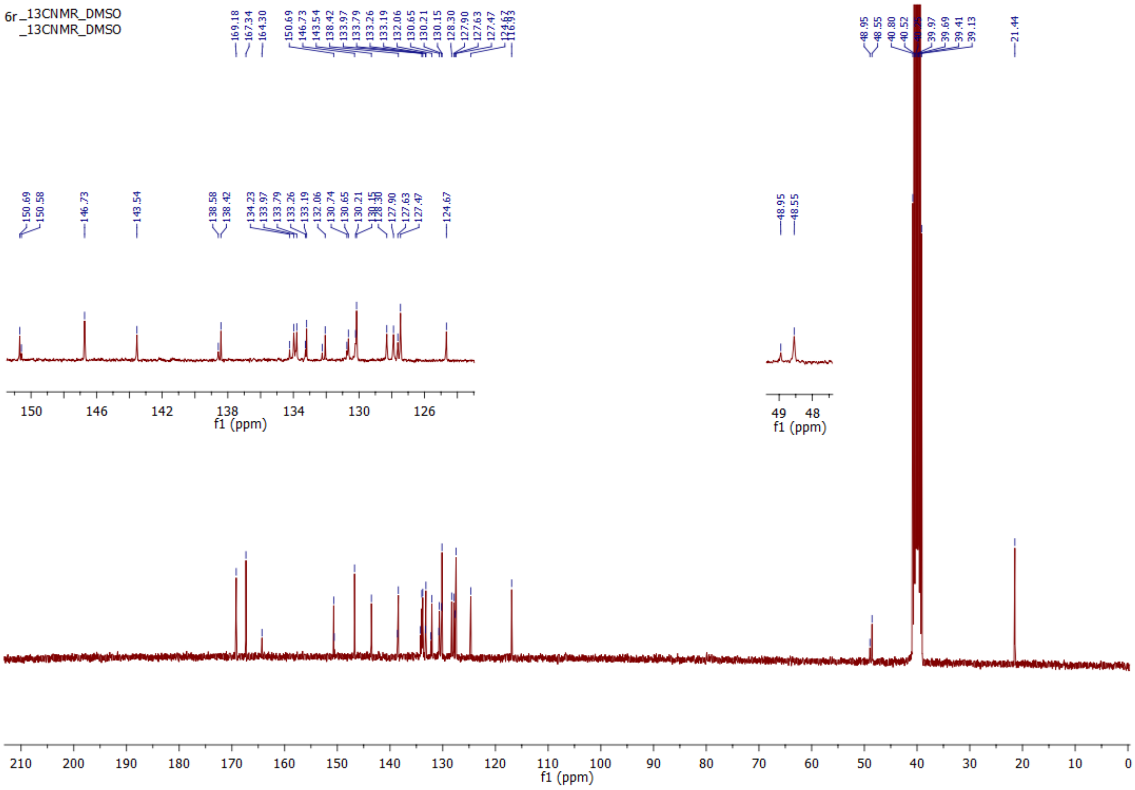

Supplementary Information

Spectra of 6s

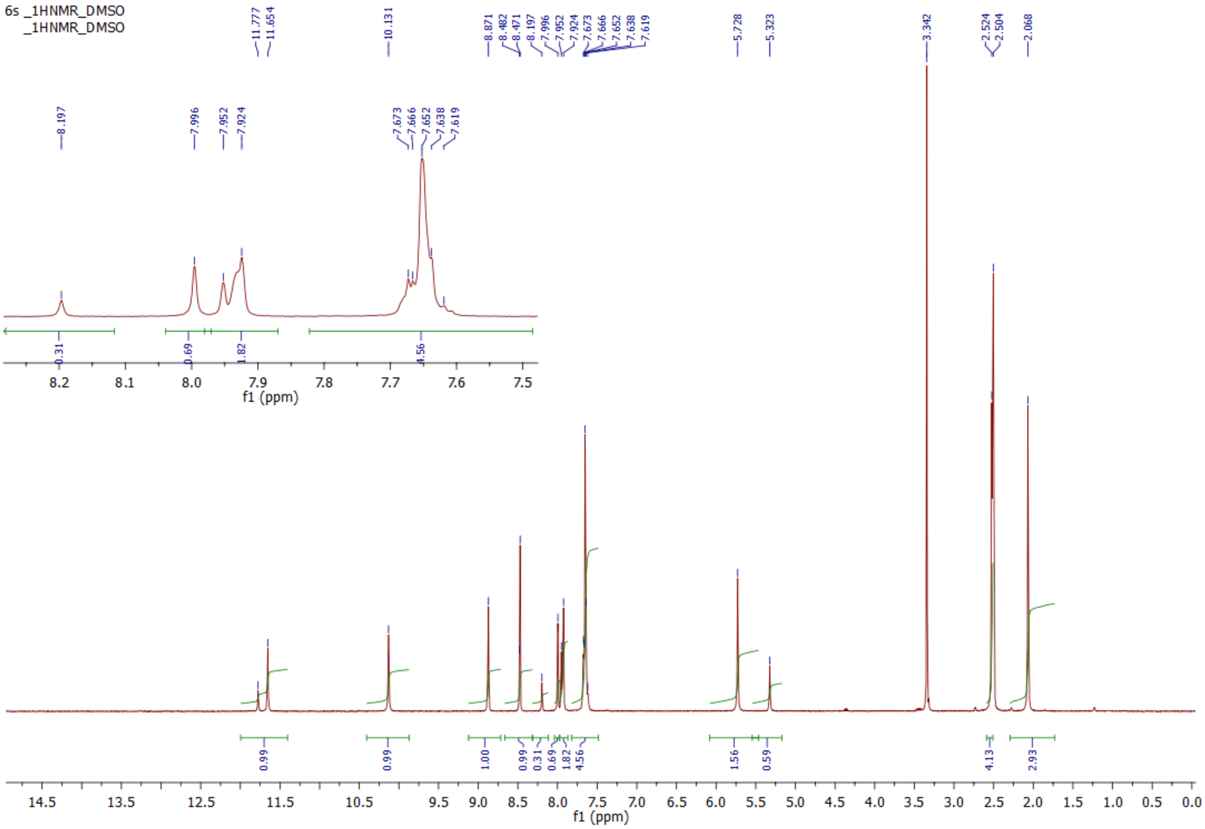

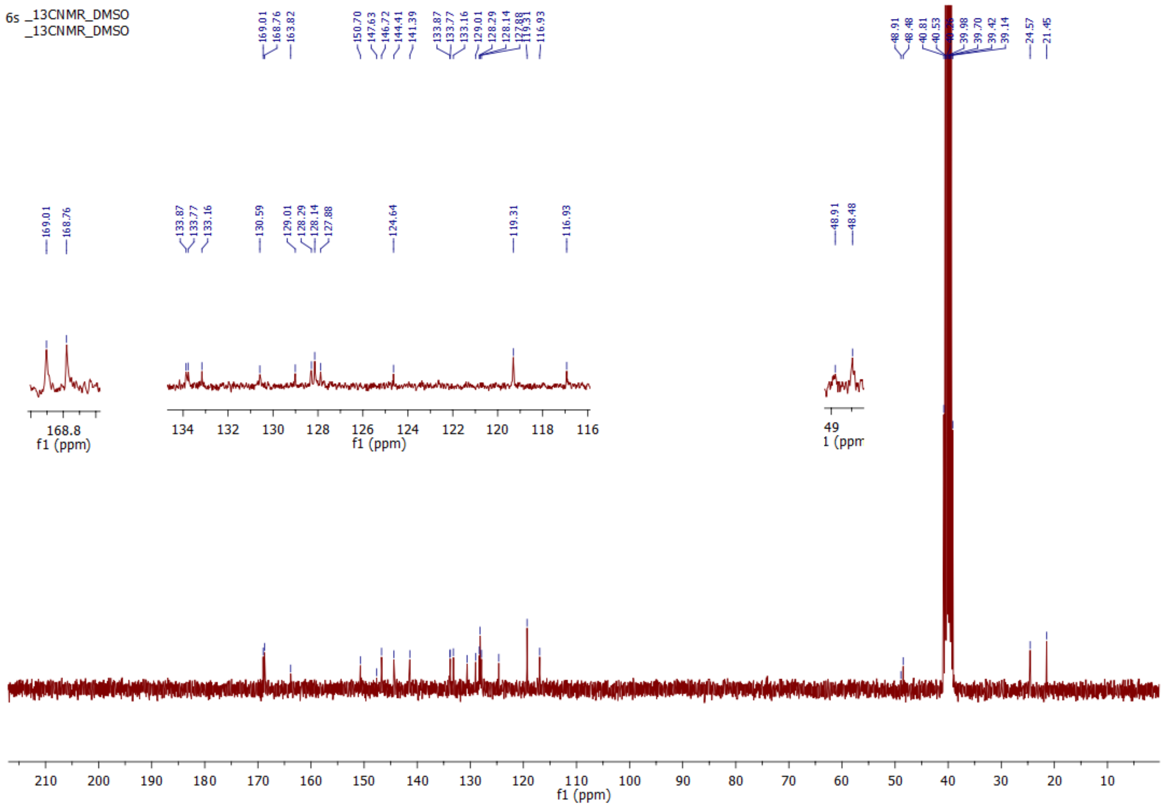

Spectra of 6t:

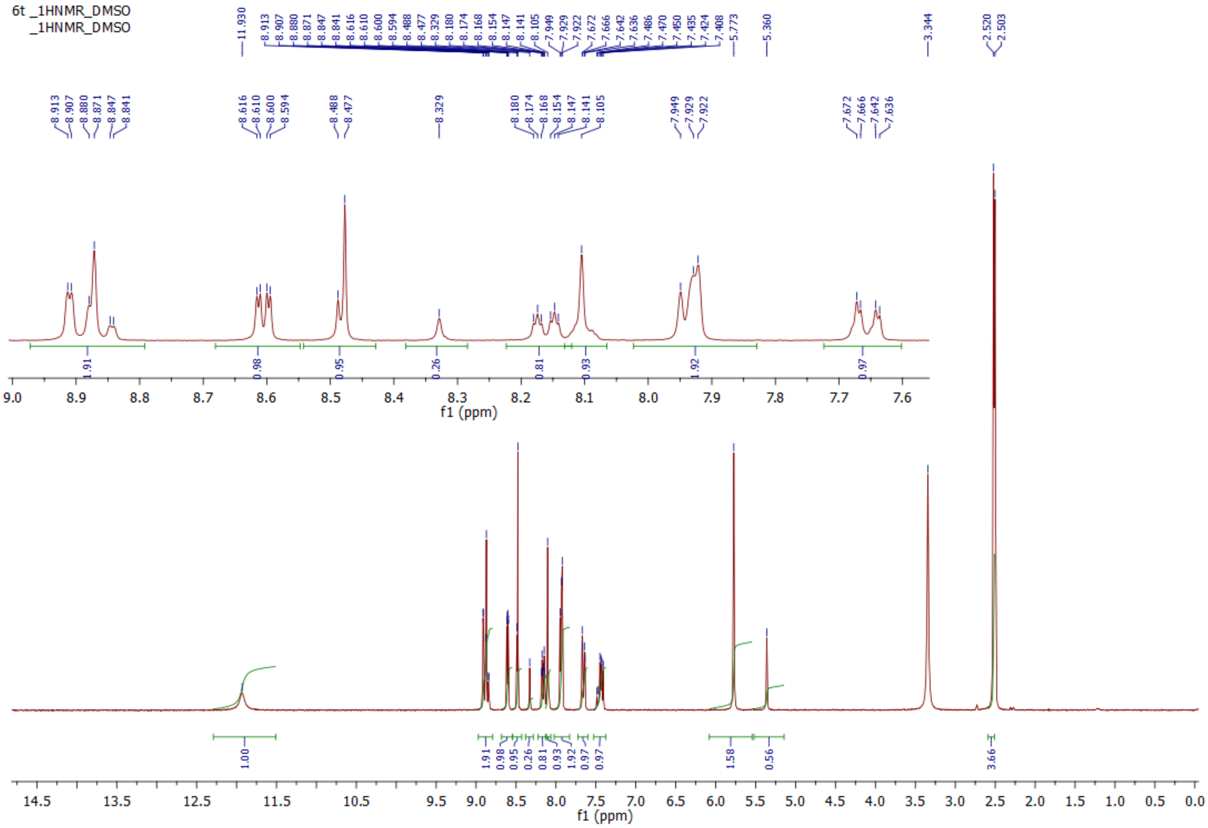

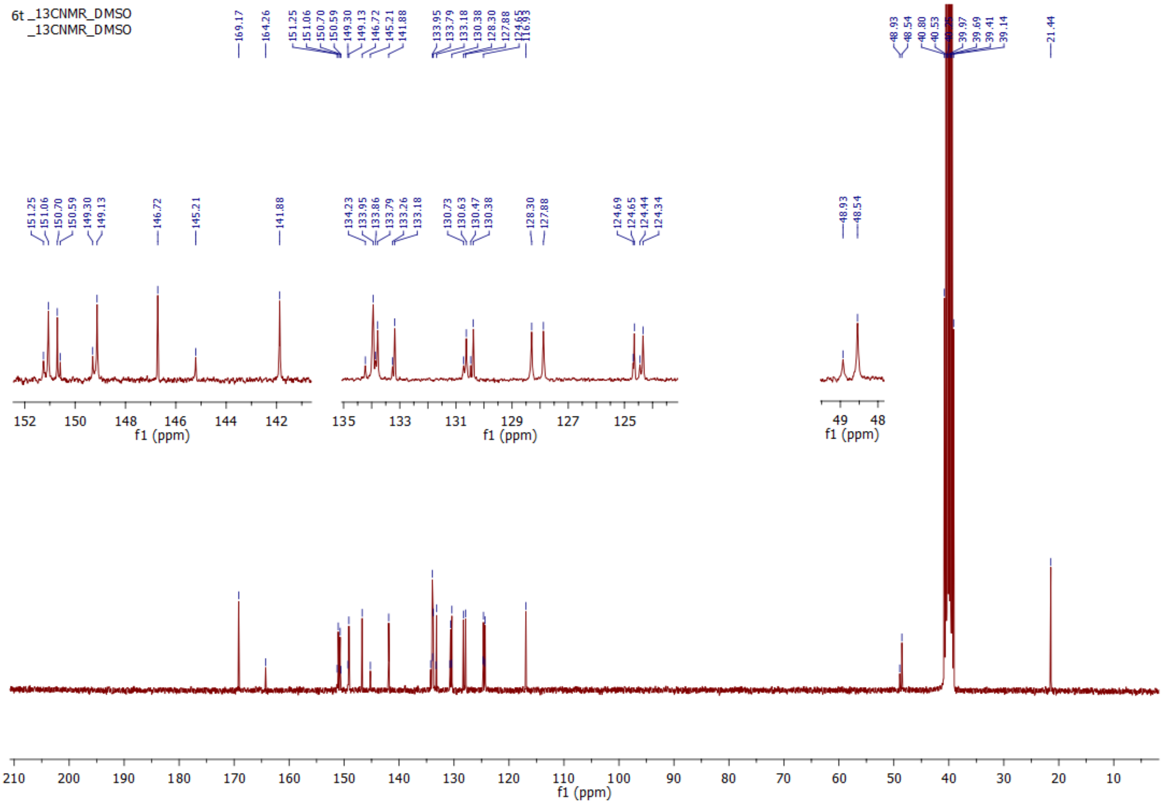

Supplementary Information

References

[1] Meth-Cohn, O.; Narine, B.; Tarnowski, B., *Journal of the Chemical Society, Perkin Transactions I*, **1981**, 1520-1530.

[2] Mali, J. R.; Pratap, U. R.; Jawale, D. V.; Mane, R. A., *Tetrahedron Letters*, **2010**, 51, 3980-3982.
